# Complexes of resorcin[4]arene with secondary amines: synthesis, solvent influence on *“in-out”* structure, and theoretical calculations of non-covalent interactions

**DOI:** 10.3762/bjoc.19.109

**Published:** 2023-09-29

**Authors:** Waldemar Iwanek

**Affiliations:** 1 Bydgoszcz University of Science and Technology, Faculty of Chemical Technology and Engineering, Seminaryjna 3, 85-326 Bydgoszcz, Poland

**Keywords:** complexes, DFT calculations, hydrogen bond, resorcin[4]arene, supramolecular chemistry

## Abstract

Resorcin[4]arenes (R[4]A) are macrocyclic compounds with a cavity structure. Despite a relatively small cavity, these compounds are capable of forming complexes with small organic molecules. The current paper focuses on the synthesis of complexes between R[4]A and secondary aliphatic amines (sec-amines). Through NMR spectroscopy, it was observed that “*in-out*” complexes are formed depending on the solvent. It was also found that the stoichiometry of the formed complexes depends on the size of the amine molecule. The automated interaction sites screening (aISS) made it possible to generate molecular ensembles of complexes. The geometry of the ensembles was first optimized with the r2scan-3c functional and, finally, the structure with the lowest energy, with the functional PBE0-D4/mTZVPP/CPCM. The Hartree–Fock plus London dispersion (HFLD) method was used for the study of non-covalent interactions (NCI). The calculations lead to the conclusion that a reduction in electrostatic interactions and an increase in exchange and dispersion interactions in CHCl_3_ in relation to DMSO are the driving forces behind the placement of sec-amine molecules into the R[4]A cavity and the formation of “*in*” type complexes.

## Introduction

Resorcin[4]arenes are macrocyclic compounds with a cavity structure formed from resorcinol and aldehydes [1]. Of particular significance is their crown conformation, which stems from the presence of 8 hydroxy groups on the upper rim and a hydrophobic cavity [2]. This property plays an important role in their self-association and the formation of larger supramolecular structures such as dimers [3], hexamers [4–5], octamers [6], and nano-aggregates [7]. The stability of these structures is highly reliant on the type of solvent used. Most of the works are studies of non-polar environments such as chloroform and toluene, in which the forming structures are stabilized by hydrogen bonds. The last few years have seen an increase in works showing the course of various types of reactions in the cavities of hexameric systems (closed spaces) created by R[4]A [8].

Complexes of R[4]A with amines [9], amino alcohols [10], and amino acids [11] have been studied due to their potential applications in supramolecular chemistry, drug delivery, and molecular recognition. The complexation of R[4]A with amines is based on the formation of hydrogen bonds between the hydroxy groups of the resorcinol units and the amine nitrogen of the guest molecule. In addition to hydrogen bonding, other interactions such as π–π stacking and electrostatic interactions also play a role in the complexation process. These interactions can be modulated by changing the pH, solvent, and temperature of the solution. The binding affinity and selectivity of the R[4]A–amine complexes depend on several factors, including the size, shape, and functional groups of both the host and the guest molecules. For example, R[4]A derivatives with different substituents on the aromatic rings [12] have been synthesized to enhance the binding affinity and selectivity towards specific amines. In addition to their potential applications in sensing and molecular recognition, R[4]A have also been studied for their potential pharmaceutical [13] and biochemical [14] applications.

R[4]A typically exhibit complex structures containing over a hundred atoms, presenting a challenging and time-consuming task for geometry optimization calculations and screening via DFT methods. Recent advancements have introduced fast semi-empirical DFT methods such as xTB [15], which enable their use in preliminary screening for molecular dynamics or searching for reactive sites (aISS), for example, in the formation of supramolecular complexes [16]. The particularly fast GFN-xTB methods work well in geometry optimization, which is the most time-consuming step of the DFT calculation. There have also been developments in DFT methods concerning the energy distribution of non-covalent interactions within various types of supramolecular complexes [17]. One such method is HFLD [18], which can be considered a dispersion-corrected HF approach where the dispersion interaction between fragments is added at the DLPNO-CC level. The HFLD method demonstrates comparable performance to HF in terms of total interaction energies while maintaining the accuracy of DLPNO CCSD(T) [19]. This method proves very accurate in quantifying non-covalent interactions, such as those found in hydrogen-bonded systems, among others.

Despite the relatively small cavity in the structure of unsubstituted R[4]A, the current work shows that these molecules are capable of forming complexes with small organic molecules using their cavity. The synthesis of R[4]A complexes with secondary aliphatic amines is presented where the stoichiometry of the complexes was found to depend on the size of the bound amine, and the type of complex formed is influenced by the nature and polarity of the solvent. The stoichiometry and solvent-dependent structure of the “*in-out*” complexes formed were determined through NMR spectroscopy. Screening tests were carried out using the aISS-xTB2 method, while the final geometries of the complexes were calculated using DFT methods. To evaluate the energy stability of the formed complexes, the HFLD method was employed to quantify the contributions of different non-covalent interactions. As shown below, the combination of two types of interactions, one of which is hydrophobic (cavity) and the other directional due to hydrogen bonds (hydroxy groups), leads to the formation of these complexes.

## Results and Discussion

**Scheme 1:**
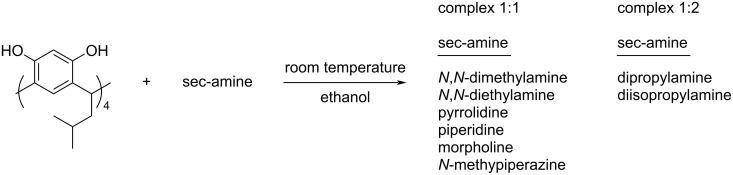
Scheme of the formation of R[4]A complexes with sec-amines in ethanol depending on the amine used.

The addition of two equivalents of sec-amines to an ethanol solution of R[4]A results in the formation of a crystalline precipitate with limited solubility in non-polar solvents. When using *N*,*N*-dimethylamine, *N*,*N*-diethylamine, pyrrolidine, piperidine, morpholine and *N*-methylpiperazine as sec-amine component, the ^1^H NMR spectra reveal the formation of complexes with a 1:1 stoichiometry. However, for aliphatic amines with longer hydrocarbon chains, like dipropylamine and diisopropylamine, complexes with a stoichiometry of 1:2 are formed (i.e., 1 molecule of R[4]A binding to 2 molecules of dipropylamine or diisopropylamine). Regardless of the quantity of amine added to the solution of R[4]A in ethanol (4 equiv, 2 equiv, and 1 equiv, respectively), a 1:2 stoichiometry complex is consistently formed for these amines. The stoichiometry of all complexes was calculated by integrating the amine signals relative to the methine proton in R[4]A and Scheme 1 shows the formation of the respective complexes depending on the amine used.

**Figure 1:**
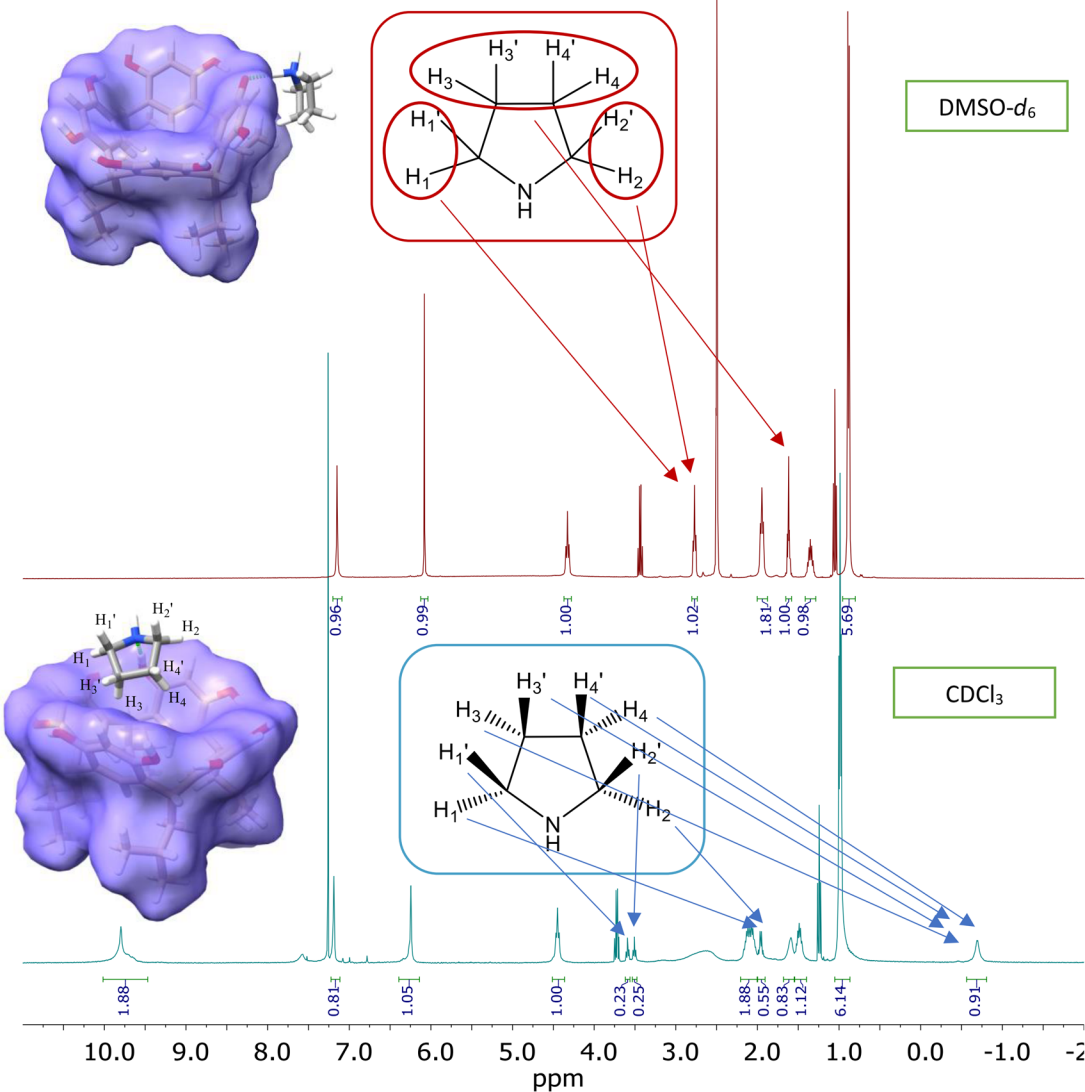
^1^H NMR spectra of the 1:1 complex of R[4]A with pyrrolidine in DMSO-*d*_6_ and CDCl_3_ solution. The integration values of the pyrrolidine molecule proton signals are highlighted in blue.

The ^1^H NMR spectra of the 1:1 stoichiometry complexes recorded in DMSO-*d*_6_ and CDCl_3_ solution are very different. On the other hand, complexes with a 1:2 stoichiometry are insoluble in CDCl_3_, hence their further analysis is limited to the solution in DMSO. Figure 1 shows the ^1^H NMR spectra of the 1:1 complex of R[4]A with pyrrolidine in DMSO-*d*_6_ and CDCl_3_, respectively.

In DMSO, the resonances of the triplet and multiplet protons of the pyrrolidine molecule are located at 2.77 ppm and 1.62 ppm, respectively. On the other hand, in CDCl_3_, the protons of the pyrrolidine molecule are located at the following ppm: 3.59 (t), 3.51(t), 1.96 (m), 1.59 (s), and −0.70 (s). This significant variation of the chemical shifts of these protons, including negative values, indicates that the protons of the pyrrolidine molecule become diastereotopic, and the factor differentiating their chemical shifts is the R[4]A cavity into which the pyrrolidine molecule is located. The assignment of the appropriate proton signals in the complex was achieved through the analysis of 1D and 2D NMR spectra (see Supporting Information File 1).

**Scheme 2:**
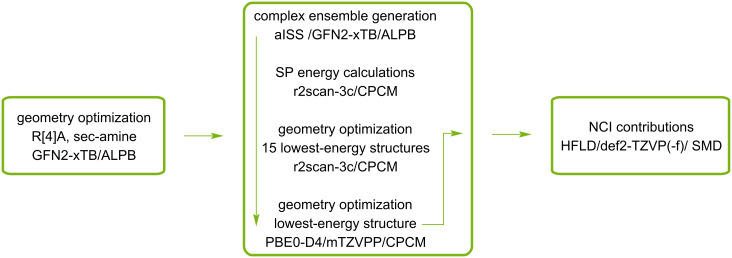
The procedure for finding the most energetically stable complex R[4]A with a sec-amine and calculating the contributions of intermolecular interactions in these complexes.

Recently, an automated interaction site screening (aISS) procedure was used to find the optimal geometry of the forming complexes. Following the initial optimization of the geometry of the R[4]A molecule and the corresponding amine using the GFN2-xTB method in DMSO and CHCl_3_ with the analytical linearized Poisson–Boltzmann (ALPB) [20] solvent model, it was subjected to the aISS procedure. This involved interaction site screening and genetic optimization with the xTB-IFF energy, followed by GFN2-xTB geometry optimizations of the generated complexes. Subsequently, the obtained complexes were sorted using the CREST program [21] with an energy threshold of 0.25 kcal/mol and an RMSD threshold of 0.25 Å. This yielded approximately 100 structures of complexes, depending on the amine used in the calculations. The one-point energies of these complexes (SP) were further refined using the r2scan-3c functional [22] with the conductor-like polarizable continuum model (CPCM) solvent model [23]. Geometric optimization using the r2scan-3c/CPCM functional in an appropriate solvent was applied to the 15 complexes with the lowest energy. The geometry of the lowest energy complex was then reoptimized using the PBE0 functional [24], the D4 dispersion correction [25], the def2-mTZVPP functional basis [21], and the CPCM solvent model. This final geometry was used to calculate the contributions of non-covalent interactions in complex formation using the HFLD method with the recommended def2-TZVP(-f) base set and the universal solvation model (SMD) [26]. The procedure outlined above for determining the most energetically stable R[4]A complex with amines described above is presented in Scheme 2.

For complexes with 1:2 stoichiometry, the procedure was similar, with the only difference being that the most stable 1:1 complex was generated using the aISS method. Subsequently, the second amine molecule was added, and the set of complexes with 1:2 stoichiometry was searched.

Theoretical calculations were performed to identify the most energetically stable complex with 1:1 stoichiometry between the R[4]A molecule and sec-amine molecules in DMSO and CHCl_3_. The calculations resulted in different geometries for these solvents. In DMSO, all the described amines formed a complex with R[4]A, in which the amine molecule is located outside the R[4]A molecule (“*out*” complex). In the case of small amine molecules such as *N*,*N*-dimethylamine, *N*,*N*-diethylamine, pyrrolidine, and piperidine, the calculations revealed a structure in which a proton is transferred from the hydroxy group of R[4]A to the amine molecule, forming a hydrogen bond between the proton of the positively charged amino group and the oxygen anion in the R[4]A molecule (ArO^−^···H^+^NHR_2_). For sec-amine molecules such as morpholine and *N*-methylpiperazine, an “*out”* complex is formed by hydrogen bonding between the proton of the hydroxy group of R[4]A and the nitrogen atom of the amine molecule (ArOH···NHR_2_).

**Figure 2:**
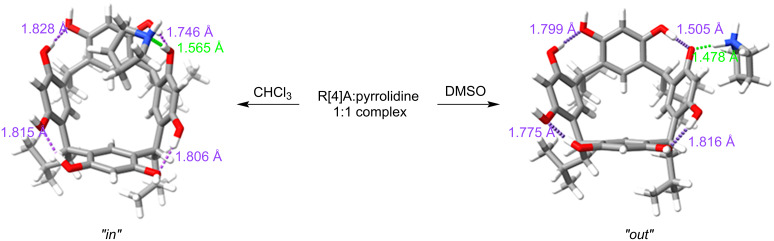
Theoretically calculated (PBE0-D4/def2-mTZVPP/CPCM) structures of the R[4]A:pyrrolidine complex in DMSO and CHCl_3_. The lengths of hydrogen bonds between the R[4]A molecule and the pyrrolidine molecule are marked in green, while the lengths of hydrogen bonds in the upper ring of R[4]A between hydroxy groups and oxygen atoms are marked in purple.

In CHCl_3_, the amine molecule partially resides within the R[4]A cavity and the formed complex is stabilized by a hydrogen bond between the hydroxy group of R[4]A and the nitrogen atom of the amine molecule (ArOH···NHR_2_). Figure 2 illustrates the theoretically calculated structures of the complexes in DMSO and CHCl_3_ using the example of the R[4]A:pyrrolidine complex, along with the corresponding hydrogen bond lengths.

The lengths of the hydrogen bonds between the R[4]A molecule and the pyrrolidine molecule are marked in green, 1.478 Å in DMSO and 1.565 Å in CHCl_3_. Additionally, the lengths of hydrogen bonds between the protons of the hydroxy groups and the oxygen atoms in the upper ring of R[4]A are marked in purple. It is worth noting the significantly shorter length of the hydrogen bond between the oxygen anion of the hydroxy group (which also forms a hydrogen bond with the amine molecule) and the adjacent hydroxy group in DMSO in the R[4]A (1.505 Å) molecule than the remaining intramolecular hydrogen bonds.

In CHCl_3_, both the type and length of the hydrogen bond undergo changes. The hydrogen bond is formed between the nitrogen atom of the amine molecule and the proton of the hydroxy group of the R[4]A molecule, with a length of 1.565 Å, which is longer than in DMSO. The calculations also reveal that the R[4]A cavity adapts slightly to the size of the amine, resulting in a slight change in its conformation towards the boat conformation. The length of the hydrogen bond between the oxygen atom of the hydroxy group forming the hydrogen bond with pyrrolidine and the adjacent hydroxy group is 1.746 Å and is only slightly shorter than the other intramolecular hydrogen bonds in R[4]A.

The above theoretical calculations of the geometry of the R[4]A:pyrrolidine complex are in good agreement with the ^1^H NMR spectra in DMSO and CDCl_3_ (Figure 1). In the case of DMSO, we observe only single sharp signals of aromatic protons (ArH) from the upper R[4]A rim of the complexes formed at δ = 7.16 ppm. This is probably due to the rapid exchange of protons between the hydroxy groups of R[4]A, while leading to even greater stabilization of the upper ring of R[4]A. In contrast, in CDCl_3_, the chemical shifts of the aromatic protons of the upper rim R[4]A in the complex are varied and slightly broadened. The three aromatic protons are located at δ = 7.19 ppm, while the more broadened signal of the aromatic ring proton whose hydroxy group is involved in the formation of a hydrogen bond with the amine is shifted towards the lower magnetic field and is located at δ = 7.59 ppm.

Based on calculations of the geometry of the complexes being formed with a 1:1 stoichiometry, Table 1 shows the lengths of the respective hydrogen bonds between the molecules of the tested amines and R[4]A. These hydrogen bonds include the hydrogen bond between the amine molecule and the R[4]A molecule, as well as the adjacent hydrogen bond between the hydroxy group of the neighboring resorcinol unit and the oxygen of the hydroxy group in the hydrogen bond with the amine molecule.

**Table 1:** Lengths of hydrogen bonds (Ångstrom) in complexes with 1:1 stoichiometry between amines and R[4]A molecules (shown in green in Figure 3), and intramolecular hydrogen bonds between the hydroxy group of the adjacent resorcinol unit and the oxygen of the hydroxy group involved in hydrogen bonding with the amine molecule (shown in purple in Figure 3). Geometry optimization was calculated using the PBE0-D4/def2-mTZVPP functional in CHCl_3_ and DMSO.

sec-amine	ArOH···NHR_2_/ArOH···HOAr^a^	ArO^−^···H^+^NHR_2_/ArO^−^···HOAr^b^

*N*,*N*-dimethylamine	1.559/1.704	1.457/1.517
*N*,*N*-diethylamine	1.562/1.720	1.497/1.502
pyrrolidine	1.565/1.746	1.478/1.505
piperidine	1.568/1.744	1.475/1.514

	ArOH···NHR_2_/ArOH···HOAr	ArOH···NHR_2_/ArOH···HOAr

morpholine	1.628/1.762	1.541/1.690
*N*-methylpiperazine	1.607/1.732	1.517/1.680

^a^in CHCl_3_; ^b^in DMSO.

**Figure 3:**
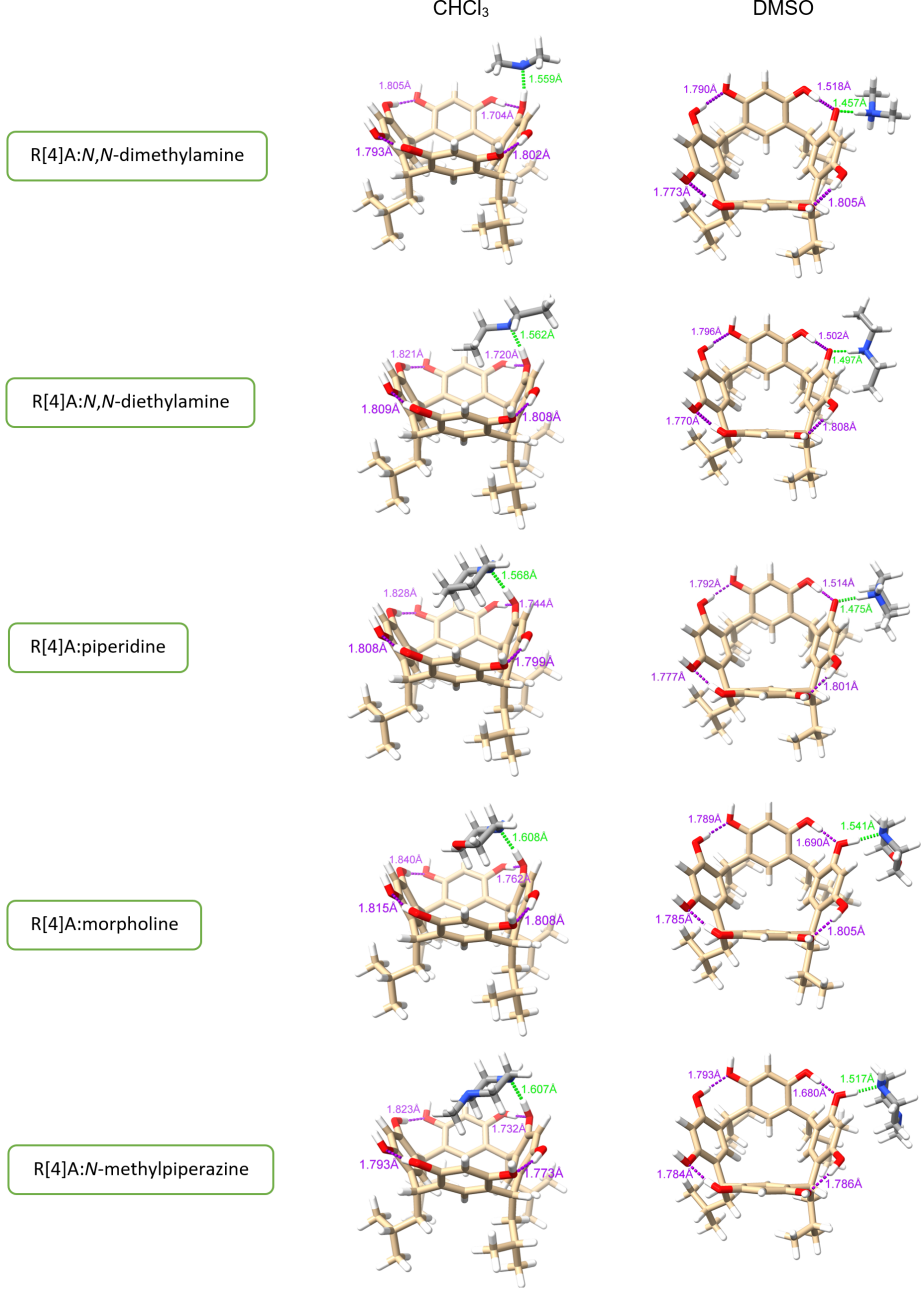
Structures of complexes with 1:1 stoichiometry between R[4]A and sec-amines in CHCl_3_ and DMSO, calculated using the PBE0-D4/def2-mTZVPP/CPCM functional.

Figure 3 shows the structures of R[4]A complexes with sec-amines with 1:1 stoichiometry in DMSO and CHCl_3_ calculated using the PBE0-D4/def2-mTZVPP/CPCM functional.

**Figure 4:**
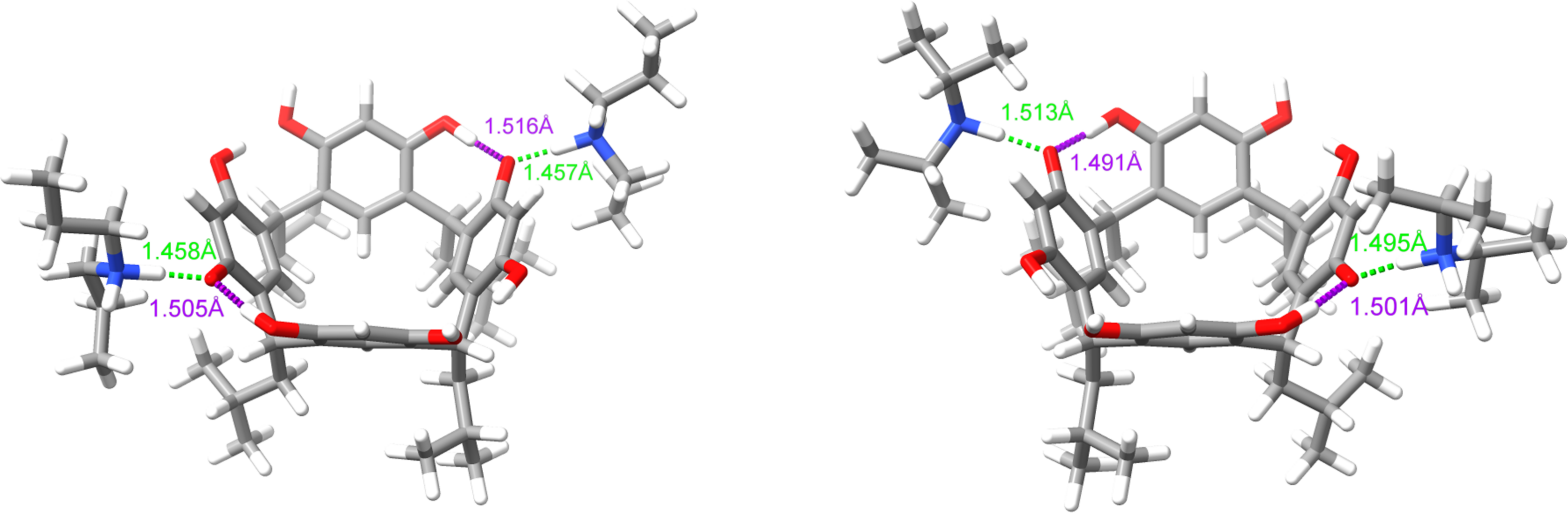
Geometrically optimized (PBE0-D4/def2-mTZVPP/CPCM(DMSO) structures of complexes with 1:2 stoichiometry between R[4]A and dipropylamine and diisopropylamine. The intermolecular hydrogen bonds between the amines and R[4]A molecules are marked in blue, while the intramolecular hydrogen bonds between the oxygen anion and the hydroxy group in R[4]A are shown in violet.

As already mentioned, complexes with 1:2 stoichiometry are formed between R[4]A and amines such as dipropylamine and diisopropylamine. The procedure of searching complex structures was carried out as outlined in Scheme 2. Geometry calculations using the r2scan-3c/CPCM(DMSO) functional revealed that the most energetically stable structure for these complexes in DMSO is an ionic complex in which two amine molecules are connected through a hydrogen bond (ArO^−^···H^+^NHR_2_) with two opposing resorcinol units in R[4]A. Figure 4 shows the geometrically optimized (PBE0-D4/def2-mTZVPP/CPCM(DMSO) structures of these complexes. The lengths of intermolecular hydrogen bonds between R[4]A and amines are marked in green, while the intramolecular hydrogen bonds between the oxygen anion and the hydrogen atom of the adjacent hydroxy group in R[4]A are marked in violet. The highly ionic structure of these complexes makes them insoluble in non-polar solvents such as CHCl_3_.

**Figure 5:**
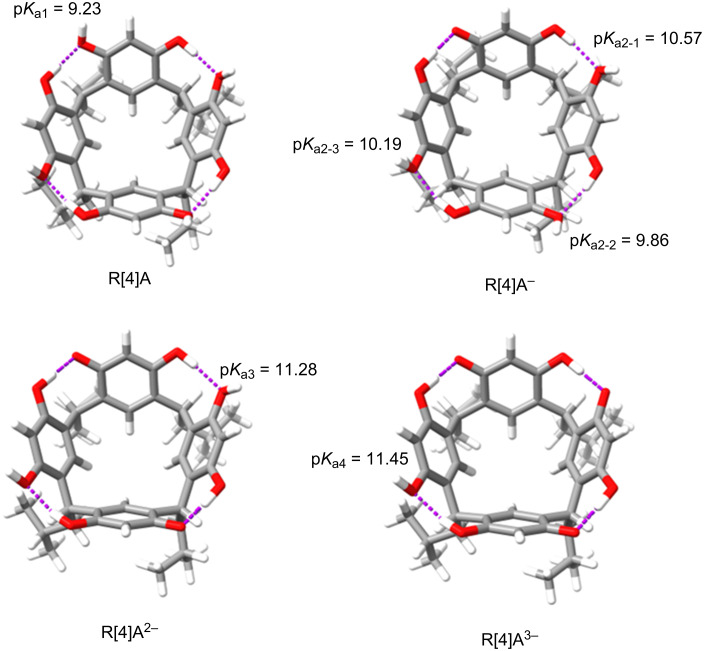
p*K*_a_ values of protons of hydroxy groups not involved in the formation of hydrogen bonds in the R[4]A molecule calculated using the GFN2-xTB/ALPB(H_2_O) method. The p*K*_a_ values are indicated at the corresponding protons of the hydroxy groups.

To explain the opposite arrangement of the amine molecules in the 1:2 complexes, the p*K*_a_ of the protons of the hydroxy groups in R[4]A not involved in the formation of hydrogen bonds was calculated. The calculations following Equation 1 were performed using the CREST/GFN2-xTB/ALBP(H_2_O) program [27], which automatically calculates the p*K*_a_ of the indicated protons. The obtained results are shown in Figure 5.


[1] $$\text{p}K_{a}=\text{c}1+\text{c}2*k_{\text{diss}}+\text{c}3*k_{\text{diss}}{}^{2}+\text{c}4*k_{\text{diss}}{}^{3}$$

with *k*_diss_ = Δ*G*_(aq)_/ln(10) *RT* and c1 = −1656.6999511, c2 = 23.18499947, c3 = −0.11103000, c4 = 0.00018350.

The calculated p*K*_a1_ value of dissociation of the first proton in R[4]A is the lowest and amounts to 9.23. It is in good agreement with the experimental data for the resorcinol molecule (experimental p*K*_a1_ value = 9.20 [28]). In the created anion R[4]A^−^ three consecutive protons can be detached, with the proton of the hydroxy group in the resorcinol unit of the anion R[4]A^−^ located opposite to the unit from which a proton had previously been detached exhibiting the highest acidity (lowest value p*K*_a2-2_ = 9.86). The next two protons of the hydroxy groups in the anion R[4]A^−^ are “less acidic”, which seems to explain the position of the amine molecules in the 1:2 complex. Interestingly, the “proton acidity” in the anions R[4]A^2−^ (p*K*_a3_ = 11.28) and R[4]A^3−^ (p*K*_a4_ = 11.45) is more than two orders of magnitude lower than the first proton of the hydroxy group in R[4]A (p*K*_a1_ = 9.23). This fact may justify the absence of the formation of complexes with 1:3 and 1:4 stoichiometry.

To evaluate the contribution of various types of intermolecular interactions in stabilizing the formation of R[4]A complexes with amines, calculations were performed using the HFLD method with the def2-TZVP(-f) basis and the SMD solvent model. This method is particularly accurate for the quantiﬁcation of non-covalent interactions found in hydrogen-bonded systems, dispersion-bound systems, and electrostatically bound systems. Table 2 presents the calculated results of non-covalent interactions (NCI) in the studied complexes, categorized as electrostatic, exchangeable, dispersion, and charge-transfer interactions.

**Table 2:** Non-covalent interactions calculated using the HFLD/def2-TZVP(-f)/SMD method in R[4]A complexes with different sec-amines, with 1:1 stoichiometry in DMSO and CHCl_3_ and 1:2 stoichiometry in DMSO. The columns show the share of individual types of NCI in electron volts (eV) and their percentage (%).

R[4]A:amine 1:1 complex	solvent	electrostatics eV (%)	exchange eV (%)	dispersion eV (%)	charge transfer eV (%)

*N*,*N*-dimethylamine	DMSO CHCl_3_	−0.35517 (88.7) −0.19544 (80.2)	−0.02840 (7.1) −0.02936 (12.1)	−0.01121 (2.8) −0.01122 (4.6)	−0.00578 (1.4) −0.00646 (2.6)
*N*,*N*-diethylamine	DMSO CHCl_3_	−0.34138 (86.4) −0.20960 (76.7)	−0.03042 (7.7) −0.03498 (12.8)	−0.01651 (4.2) −0.02103 (7.7)	−0.00653 (1.7) −0.00754 (2.8)
pyrrolidine	DMSO CHCl_3_	−0.35000 (86.0) −0.21280 (75.4)	−0.03110 (7.6) −0.03690 (13.0)	−0.01709 (4.2) −0.02469 (8.8)	−0.00888 (2.2) −0.00794 (2.8)
piperidine	DMSO CHCl_3_	−0.35497 (87.2) −0.21758 (74.0)	−0.03136 (7.7) −0.03947 (13.4)	−0.01444 (3.5) −0.02841 (9.7)	−0.00626 (1.5) −0.00844 (2.9)
morpholine	DMSO CHCl_3_	−0.20819 (79.4) −0.18540 (74.9)	−0.03233 (12.3) −0.03393 (13.7)	−0.01477 (5.6) −0.02291 (9.2)	−0.00679 (2.6) −0.00545 (2.2)
*N*-methylpiperazine	DMSO CHCl_3_	−0.22712 (79.0) −0.20600 (72.0)	−0.03537 (12.3) −0.04008 (14.0)	−0.01766 (6.2) −0.03191 (11.1)	−0.00721 (2.5) −0.00830 (2.9)

R[4]A:amine 1:2 complex					

dipropylamine	DMSO	−0.77799 (88.0)	−0.062197 (7.0)	−0.030919 (3.5)	−0.012603 (1.4)
diisopropylamine	DMSO	−0.74717 (87.6)	−0.060762 (7.1)	−0.031915 (3.7)	−0.012621 (1.5)

From the data presented in Table 2, it is evident that electrostatic interactions in the form of hydrogen bonds primarily contribute to the formation of R[4]:sec-amine complexes with a 1:1 stoichiometry. In DMSO, they account for about 79–89% of all NCI interactions, with the highest share (88.7%) observed for small molecules of amines (*N*,*N*-dimethylamine) and decreasing as the amine size increases (for *N*-methylpiperazine = 79.0%). In CHCl_3_, the share of electrostatic interactions is lower by about 8% compared to DMSO and also decreases with increasing amine size. Exchange interactions between the components of the complex are generally greater in CHCl_3_ than in DMSO and slightly increase with the increasing amine particle size: ranging from 12.1% to 14% in CHCl_3_ and from 7.1% to 12.3% in DMSO. On the other hand, the dispersion interactions are about twice as high in CHCl_3_ compared to DMSO and increase with the size of the amine molecule. Their percentage value, however, is slightly lower than that of the exchange interactions and ranges from 2.8% to 11.1%. The smallest contribution to the non-covalent interactions stabilizing the formation of 1:1 stoichiometry complexes was noted for the charge-transfer interactions. They are only slightly higher in CHCl_3_ than in DMSO, ranging from 1.4 to 2.9%. In general, it can be concluded from these calculations that the reduction of electrostatic interactions and the increase in exchange and dispersion interactions in CHCl_3_ compared to DMSO are the driving forces behind the placement of sec-amine molecules into the R[4]A cavity and the formation of “*in”*-type complexes.

In the case of complexes with 1:2 stoichiometry, the shares of individual types of NCI in DMSO are as follows: approx. 88% electrostatic, 7% exchangeable, 3.6% dispersion, and 1.5% charge transfer. It is noteworthy that despite the presence of relatively large aliphatic chains in these amine molecules, electrostatic interactions strongly dominate in these complexes. This dominance leads to the formation of ionic complexes, which, as already mentioned, are insoluble in CHCl_3_. Therefore, calculations of non-covalent interactions using the HFLD method were not performed for this solvent.

## Conclusion

Complexes of aliphatic sec-amines with variable size alkyl substituents with R[4]A in ethanol were synthesized. The composition of the complexes was determined based on the integration of amine proton signals in the ^1^H NMR spectra. For small molecule sec-amines such as *N*,*N*-dimethylamine, *N*,*N*-diethylamine, and cyclic amines (pyrrolidine, piperidine, morpholine, *N*-methylpiperazine), the complexes with 1:1 stoichiometry were formed. For sec-amines with larger alkyl substituents (dipropylamine, diisopropylamine), complexes with 1:2 stoichiometry were formed. The structure of the complexes with 1:1 stoichiometry varies depending on the type and polarity of the solvent. In DMSO, “*out*” complexes are formed, while in CHCl_3_, “*in*” complexes are formed. With the fast semi-empirical DFT (xTB), ensembles of complexes were searched using the aISS docking module. The generated complex ensembles were segregated with the CREST program, and then their structures were optimized using the r2scan-3c/CPCM(solvent) method in a suitable solvent. The geometry of the lowest energy structure was further optimized using the PBE0 functional with the def2-mTZVPP functional basis and the CPCM solvent model. On the basis of the as such optimized structures, the participation of various types of non-covalent interactions in the appropriate solvent was assessed using the HFLD method. The calculations show that the reduction of electrostatic interactions and the increase in exchange and dispersion interactions in CHCl_3_ compared to DMSO are the driving forces for the placement of the sec-amine molecules in the R[4]A cavity and the formation of “*in*” complexes in complexes with 1:1 stoichiometry.

The discussed results show that the most suitable sec-amines for the formation of 1:1 stoichiometry complexes are those amines whose alkyl groups are small enough to be located in the R[4]A cavity and form “*in*” complexes. These are amines in which the size of the alkyl groups does not exceed the size of the ethyl group and cyclic sec-amines with five and six-membered rings. Pyrrolidine is the sec-amine that is most “structurally matched” to the size of the R[4]A cavity. For amines with longer alkyl chains, complexes with 1:2 stoichiometry are formed. This stoichiometry is justified by calculations of the acidity of protons of hydroxy groups in the R[4]A molecule. These complexes are highly ionic because of their very low solubility in non-polar solvents. Complexes with higher stoichiometry are not formed because the acidity of protons of the third and fourth hydroxy groups in the R[4]A molecule is much lower compared to the first and second hydroxy groups.

## Experimental

The NMR spectra were recorded using an Avance 400 ultra-shield spectrometer (Bruker, Karlsruhe, Germany). Reagents and solvents were obtained from Sigma-Aldrich, Fluka, and Merck with purity class of 99% and were used without further purification. R[4]A (2,4,6,8-tetraisobutyl-1,3,5,7(1,3)-tetrabenzenacyclooctaphan-1^4^,1^6^,3^4^,3^6^,5^4^,5^6^,7^4^,7^6^-octaol) was synthesized according to the procedure described in the paper [32].

**Calculation procedure:** Optimization of the R[4]A and sec-amine geometries was performed with the GFN2-xTB method using the ALPB solvent model with the “veryTight” accuracy using the xTB software package [29]. Complex assembly search (aISS) was performed using the “dock” function with “tight” optimization of the geometry of the complex ensembles (GFN2-xTB) being searched. The set of complexes was sorted using the CREST program’s “cregen” command with sorting parameters as follows: RMSD threshold = 0.25 Å and energy threshold Δ*E* = 0.25 kcal/mol. One-point energy calculations of the sorted set were refined using the r2scan-3c functional and the CPCM solvent model using the ORCA 5.03 program [30]. The r2scan-3c/CPCM functional and the ORCA 5.0.3 program were also used to optimize the geometry of the 15 lowest energy complexes using the default geometry optimization values. The geometries of the lowest-energy complexes were refined using the PBE0 functional with D4 dispersion correction and the CPCM solvent model (ORCA5.0.3). HFLD calculations with the def2-TZVP(-f) basis and the SMD solvent model were performed using the ORCA5.03 program with RIJCOSX approach [31] and NormalPNO settings. The acidity of hydroxy groups’ protons not involved in the formation of hydrogen bonds in the R[4]A molecule was calculated with the CREST program using the “pKa” command.

**General method for the synthesis of R[4]A complexes with sec-amines:** 0.2 g (0.225 mmol) of R[4]A was weighed into a round-bottomed flask and dissolved in 20 mL of ethanol. Then, 2 equivalents of sec-amine were added and left at room temperature for 24 hours. Depending on the amine, the complex quickly precipitated or, as in the case of, e.g., morpholine or 1-methylpiperazine, slowly. The precipitate was then filtered, washed with cold ethanol, and dried. The yields of complex formation ranged from 45 to 78%.

**1:1 Complex of R[4]A with dimethylamine:** 45% yield; white solid; ^1^H NMR (400 MHz, DMSO-*d*_6_, *T* = 298 K) δ 7.15 (s, 4H, PhCH), 6.75–4.67 (br s, 8H, OH), 6.10 (s, 4H, PhCH), 4.34 (t, *J* = 7.70 Hz, 4H, CH), 2.30 (s, 6H, N(CH_3_)_2_), 1.94 (t, *J* = 6.97 Hz, 8H, CH_2_), 1.35 (m, 4H, CH), 0.88 (d, *J* = 6.60 Hz, 24H, CH_3_) ppm; ^1^H NMR (400 MHz, CDCl_3_, *T* = 298 K) δ 9.68 (s, 4H, OH), 9.58 (s, 4H, OH), 7.19 (s, 4H, PhH), 6.25 (s, 4H, PhH), 4.47 (t, *J* = 7.70 Hz*,* 4H, CH), 3.38 (br s, 6H, N(CH_3_)_2_), 2.09 (m, 8H, CH_2_), 1.50 (m, 4H, CH), 0.99 (t, *J* = 6.60 Hz, 24H, CH_3_) ppm; ^13^C NMR (100 MHz, DMSO-*d*_6_, *T* = 298 K) δ 152.0, 124.6, 123.3, 102.6, 43.7, 37.1, 30.7, 25.8, 22.8 ppm.

**1:1 Complex of R[4]A with diethylamine:** 68% yield; white solid; ^1^H NMR (400 MHz, DMSO-*d*_6_, *T* = 298 K) δ 7.16 (s, 4H, PhCH), 6.10 (s, 4H, PhCH), 5.6–3.6 (br s, 8H, OH), 4.34 (t, *J* = 7.70 Hz, 4H, CH), 2.58 (q, *J* = 6.97 Hz, 4H, N*CH_2_*CH_3_), 1.94 (t, CH_2_, *J* = 6.97 Hz, 8H, CH_2_), 1.36 (m, 4H, CH), 1.02 (t, *J* = 6.97 Hz, 6H, NCH_2_*CH_3_*), 0.88 (d, CH_3_, *J* = 6.60 Hz, 24H, CH_3_) ppm; ^1^H NMR (400 MHz, CDCl_3_, *T* = 298 K) δ 9.59 (br s, 9.59, 4H, OH), 9.18 (br s, 4H, OH), 7.36 (br s, 1H, PhCH), 7.17 (s, 3H, PhH), 6.27 (s, 4H, PhH), 4.43 (m, 4H, CH), 3.42 (q, *J* = 6.97 Hz, 2H, N*CH_2_*CH_3_), 3.41 (q, *J* = 6.97 Hz, 2H, N*CH_2_*CH_3_), 2.08 (m, 8H, CH_2_), 1.47 (m, 4H, CH), 1.24 (m, 3H, NCH_2_*CH_3_*), 0.97 (t, *J* = 6.60 Hz, 24H, CH_3_), 0.22 (br t, 3H, NCH_2_*CH_3_*) ppm; ^13^C NMR (100 MHz, DMSO-*d*_6_, *T* = 298 K) δ 151.8, 124.9, 123.2, 102.5, 42.9, 42.7, 30.7, 25.7, 22.7, 13.7 ppm.

**1:1 Complex of R[4]A with pyrrolidine:** 72% yield; white solid; ^1^H NMR (400 MHz, DMSO-*d*_6_, *T* = 298 K) δ 7.15 (s, 4H, PhCH), 6.09 (s, 4H, PhCH), 5.6–3.8 (br s, 8H, OH), 4.33 (t, *J* = 7.70 Hz, 4H, CH), 2.77 (m, 4H, N(*CH_2_*)_2_(CH_2_)_2_), 1.95 (t, *J* = 6.97 Hz, 8H, CH_2_), 1.62 (m, 4H, N(CH_2_)_2_(*CH_2_*)_2_), 1.35 (m, 4H, CH), 0.89 (d, *J* = 6.60 Hz, 24H, CH_3_) ppm; ^1^H NMR (400 MHz, CDCl_3_, *T* = 298 K) δ 9.80 (br s, 8H, OH), 7.57 (br s, 1H, PhH), 7.19 (s, 3H, PhH), 6.24 (s, 4H, PhH), 4.45 (t, *J* = 7.70 Hz, 4H, CH), 3.59 (m, 1H, N(*CH_2_*)_2_(CH_2_)_2_), 3.51 (m, 1H, N(*CH_2_*)_2_(CH_2_)_2_), 2.09 (m, 8H, CH_2_), 1.96 (m, 2H, N(*CH_2_*)_2_(CH_2_)_2_), 1.49 (m, 4H, CH), 0.99 (t, *J* = 6.24 Hz, 24H, CH_3_), −0.7 (m, 4H, N(CH_2_)_2_(*CH_2_*)_2_) ppm; ^13^C NMR (100 MHz, DMSO-*d*_6_, *T* = 298 K) δ 152.0, 124.3 123.3, 102.7, 45.7, 42.5, 31.6, 25.7, 24.6, 22.7 ppm.

**1:1 Complex of R[4]A with piperidine:** 78% yield; white solid; ^1^H NMR (400 MHz, DMSO-*d*_6_, *T* = 298 K) δ 7.8–5.8 (br s, 8H, OH), 7.16 (s, 4H, PhCH), 6.07 (s, 4H, PhCH), 4.32 (t, *J* = 7.70 Hz, 4H, CH), 2.72 (m, 4H, N(*CH_2_*)_2_(CH_2_)_3_), 1.97 (t, CH_2_, *J* = 6.97 Hz, 8H, CH_2_), 1.47 (m, 6H, N(CH_2_)_2_(*CH_2_*)_3_), 1.37 (m, 4H, CH), 0.89 (d, *J* = 6.60 Hz, 24H, CH_3_) ppm; ^1^H NMR (400 MHz, CDCl_3_, *T* = 298 K) δ 9.68 (br s, 8H, OH), 7.20 (s, 4H, PhH), 6.24 (s, 4H, PhH), 4.45 (*J* = 7.70 Hz, 4H, CH), 3.66 (m, 1H, N(*CH_2_*)_2_(CH_2_)_3_), 3.58 (m, 1H, N(*CH_2_*)_2_(CH_2_)_3_), 2.10 (m, 8H, CH_2_), 1.65 (m, 2H, N(CH_2_)_2_(*CH_2_*)_3_), 1.49 (m, 4H, CH), 0.99 (t, *J* = 6.24 Hz, 24H, CH_3_), 0.19 (s, 1H, N(CH_2_)_2_(*CH_2_*)_3_), 0.05 (s, 1H, N(CH_2_)_2_(*CH_2_*)_3_), −0.04 (s, 1H, N(CH_2_)_2_(*CH_2_*)_3_), −0.11 (s, 1H, N(CH_2_)_2_(*CH_2_*)_3_), −0.79 (m, 2H, N(CH_2_)_2_(*CH_2_*)_3_) ppm; ^13^C NMR (100 MHz, DMSO-*d*_6_, *T* = 298 K) δ 152.3, 124.1, 123.4 102.9, 45.5, 42.4, 25.8, 25.0, 23.7, 22.8 ppm.

**1:1 Complex of R[4]A with morpholine:** 62% yield; white solid; ^1^H NMR (400 MHz, DMSO-*d*_6_, *T* = 298 K) δ 8.6–6.6 (br s, 8H, OH), 7.14 (s, 4H, PhCH), 6.14 (s, 4H, PhCH), 4.36 (t, *J* = 7.70 Hz, 4H, CH), 3.51 (t, *J* = 4.77 Hz, 4H, N(CH_2_)_2_(*CH_2_*)_2_O,), 2.68 (t, *J* = 4.77 Hz, 4H, N(*CH_2_*)_2_(CH_2_)_2_O)), 1.91 (t, *J* = 6.97 Hz, 8H, CH_2_), 1.34 (m, 4H, CH), 0.88 (d, CH_3_, *J* = 6.60 Hz, 24H, CH_3_) ppm; ^1^H NMR (400 MHz, CDCl_3_, *T* = 298 K) δ 9.64 (m, 8H, OH), 7.20 (s, 4H, PhH), 6.25 (s, 4H, PhH), 4.45 (t, *J* = 7.70 Hz, 4H, CH), 3.73 (m, 3H, N(CH_2_)_4_O), 3.65 (m 1H, N(CH_2_)_4_O), 2.26 (br s, 1H, N(CH_2_)_4_O), 1.86 (br s, 1H, N(CH_2_)_4_O), 2.56 (br s, 1H, N(CH_2_)_4_O), 1.31 (br s, 1H, N(CH_2_)_4_O), 0.99 (t, *J* = 6.24 Hz, 24H, CH_3_) ppm; ^13^C NMR (100 MHz, DMSO-*d*_6_, *T* = 298 K) δ 151.8, 124.9, 123.2, 122.5, 67.0, 45.7, 43.0, 30.7, 25.8, 22.7 ppm.

**1:1 Complex of R[4]A with *N*-methylpiperazine:** 64% yield; white solid; ^1^H NMR (400 MHz, DMSO-*d*_6_, *T* = 298 K) δ 7.13 (s, 4H, PhCH), 6.14 (s, 4H, PhCH), 4.36 (t, *J* = 7.70 Hz, 4H, CH), 4.0–3.0 (br s, 8H, OH), 2.69 (t, *J* = 5.14 Hz, 4H, N(*CH_2_*)_2_(CH_2_)_2_NCH_3_), 2.21 (br s, 4H, N(CH_2_)_2_(*CH_2_*)_2_NCH_3_), 2.11 (s, 3H, N(CH_2_)_2_(CH_2_)_2_N*CH_3_*), 1.92 (t, *J* = 6.97 Hz, 8H, CH_2_), 1.34 (m, 4H, CH), 0.88 (d, *J* = 6.60 Hz, 24H, CH_3_) ppm; ^1^H NMR (400 MHz, CDCl_3_, *T* = 298 K, sparingly soluble) δ 9.66 (br s, 8H, OH), 7.19 (br s, 4H, PhH), 6.41 (br s, 4H, PhH), 4.47 (m, 4H, CH), 3.59 (br s, 2H, N(*CH*_2_)_2_(CH_2_)_2_NCH3), 2.83 (br s, 2H, N(*CH_2_*)_2_(CH_2_)_2_NCH_3_), 1.97 (br s, 13H, CH_2_, N(CH_2_)_2_(*CH_2_*)_2_N*CH_3_*), 1.48 (m. 4H, CH), 0.99 (m, 24H, CH_3_), −0.27 (br m, 2H, N(CH_2_)_2_(*CH_2_*)_2_NCH_3_) ppm; ^13^C NMR (100 MHz, DMSO-*d*_6_, *T* = 298 K) δ 151.7, 125.0, 123.1, 102.4, 55.6, 46.4, 45.2, 43.0, 30.7, 25.7, 22.7 ppm.

**1:2 Complex of R[4]A with dipropylamine:** 69% yield; white solid; ^1^H NMR (400 MHz, DMSO-*d*_6_, *T* = 298 K) δ 7.16 (s, 4H, PhCH), 6.60–4.60 (br s, 8H, OH), 6.10 (s, 4H, PhCH), 4.34 (t, *J* = 7.70 Hz, 4H, CH), 2.48 (t, *J* = 7.34 Hz, 8H, N(*CH_2_*)_2_(CH_2_)_2_(CH_3_)_2_), 1.95 (m, 8H, CH_2_), 1.41 (m, 8H, N(CH_2_)_2_(*CH_2_*)_2_(CH_3_)_2_), 1.37 (m, 4H, CH), 0.89 (d, *J* = 6.60 Hz, CH_3_), 0.86 (t, *J* = 7.34 Hz, 12H, N(CH_2_)_2_(CH_2_)_2_(*CH_3_*)_2_) ppm; ^13^C NMR (100 MHz, DMSO-*d*_6_, *T* = 298 K) δ 151.8, 124.4, 123.3, 102.6, 56.0, 50.9, 42.6, 30.7, 25.7, 22.7, 22.1, 11.6 ppm.

**1:2 Complex of R[4]A with diisopropylamine:** 71% yield; white solid; ^1^H NMR (400 MHz, DMSO-*d*_6_, *T* = 298 K) δ 7.16 (s, 4H, PhCH), 6.09 (s, 4H, PhCH), 5.00–3.60 (br s, 8H, OH), 4.33 (t, *J* = 7,70 Hz, 4H, CH), 2.87 (m, 4H, N(*CH*)_2_((CH_3_)_2_)_2_), 1.95 (m, *J* = 6.97 Hz, 8H, CH_2_), 1.35 (m, 4H, CH), 0.97 (d, *J* = 6.24 Hz, 24H, N(CH)_2_((*CH_3_*)_2_)_2_), 0.89 (d, *J* = 6.60 Hz*,* CH_3_) ppm; ^13^C NMR (100 MHz, DMSO-*d*_6_, *T* = 298 K) δ 151.9, 124.3, 123.3, 102.6, 56.0, 44.8, 42.6, 30.7, 25.7, 22.7, 22.4 ppm.

## Supporting Information

File 1 NMR spectra of complexes and coordinates of the optimized complex structures.
