# Supplementary material for: Complexes of resorcin[4]arene with secondary amines: synthesis, solvent influence on “in-out” structure, and theoretical calculations of non-covalent interactions
Source: Beilstein J Org Chem. 2023 Sep 29;19:1525–36. doi: 10.3762/bjoc.19.109 (PMC10548251; doi:10.3762/bjoc.19.109)
Supplement: File 1 — NMR spectra of complexes and coordinates of the optimized complex structures. [file Beilstein_J_Org_Chem-19-1525-s001.pdf]

BEILSTEIN JOURNAL OF ORGANIC CHEMISTRY

Supporting Information

for

Complexes of resorcin[4]arene with secondary amines: synthesis, solvent influence on *"in-out"* structure, and theoretical calculations of non-covalent interactions

Waldemar Iwanek

Beilstein J. Org. Chem. **2023,** *19,* 1525–1536. doi:10.3762/bjoc.19.109

NMR spectra of complexes and coordinates of the optimized complex structures

License and Terms: This is a supporting information file under the terms of the Creative Commons Attribution License (https://creativecommons.org/licenses/by/4.0). Please note that the reuse, redistribution and reproduction in particular requires that the author(s) and source are credited and that individual graphics may be subject to special legal provisions.
The license is subject to the *Beilstein Journal of Organic Chemistry* terms and conditions: (https://www.beilstein-journals.org/bjoc/terms)

Table of contents

1. NMR spectra of R[4]A complexes with sec-amines.
2. The xyz coordinates of the optimized complex structures calculated by the PBE0-D4/mTZVPP method in $CHCl_3$ and DMSO.

1. NMR spectra of R[4]A complexes with sec-amines

R[4]A:dimethylamine

^{1}H NMR in DMSO

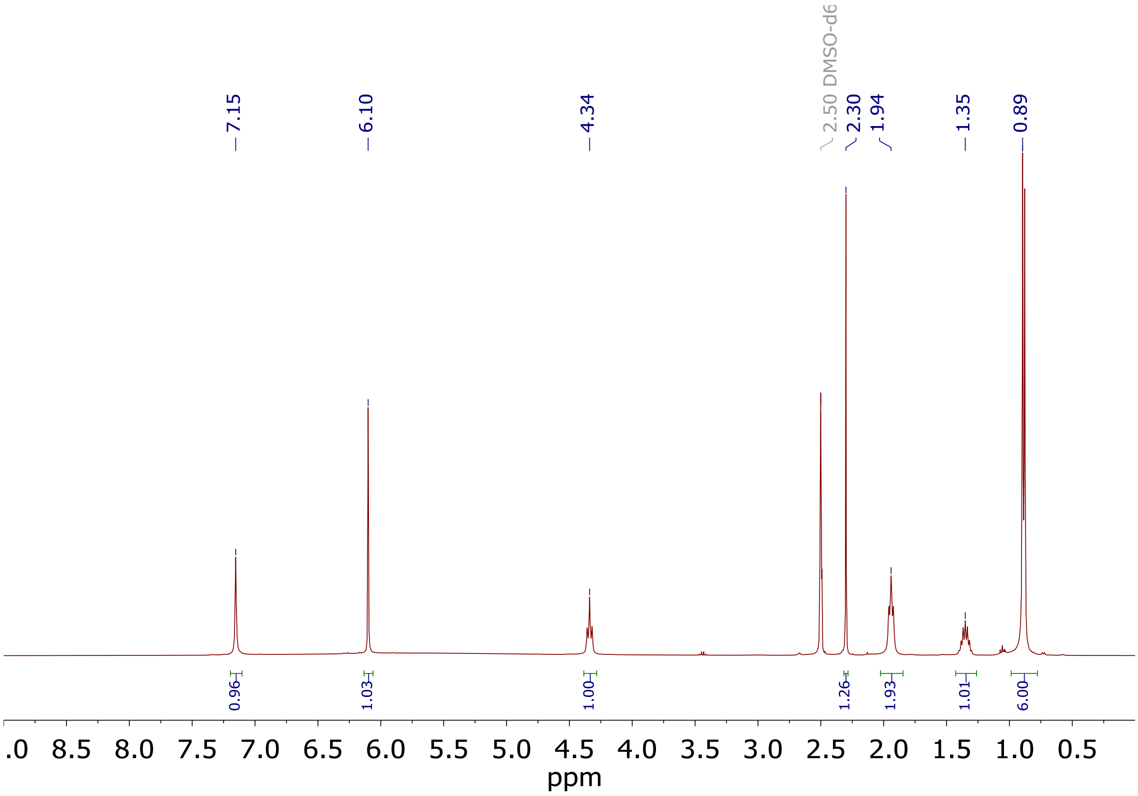

^{1}H NMR in $CDCl_3$

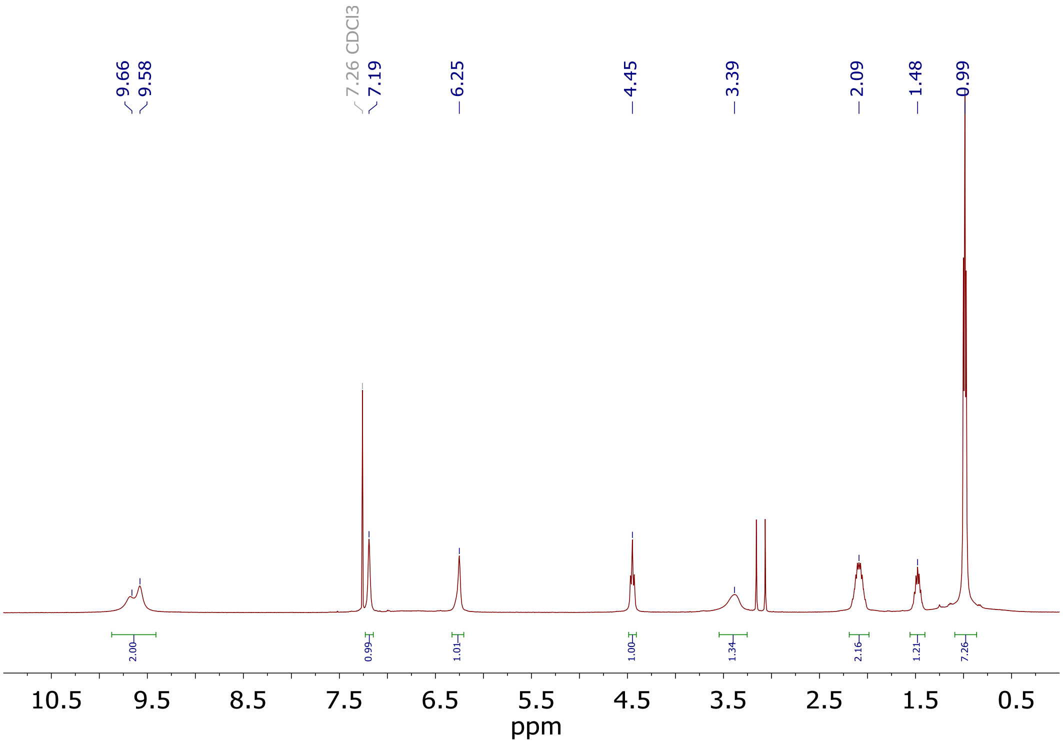

^{13}C NMR in DMSO-d_6

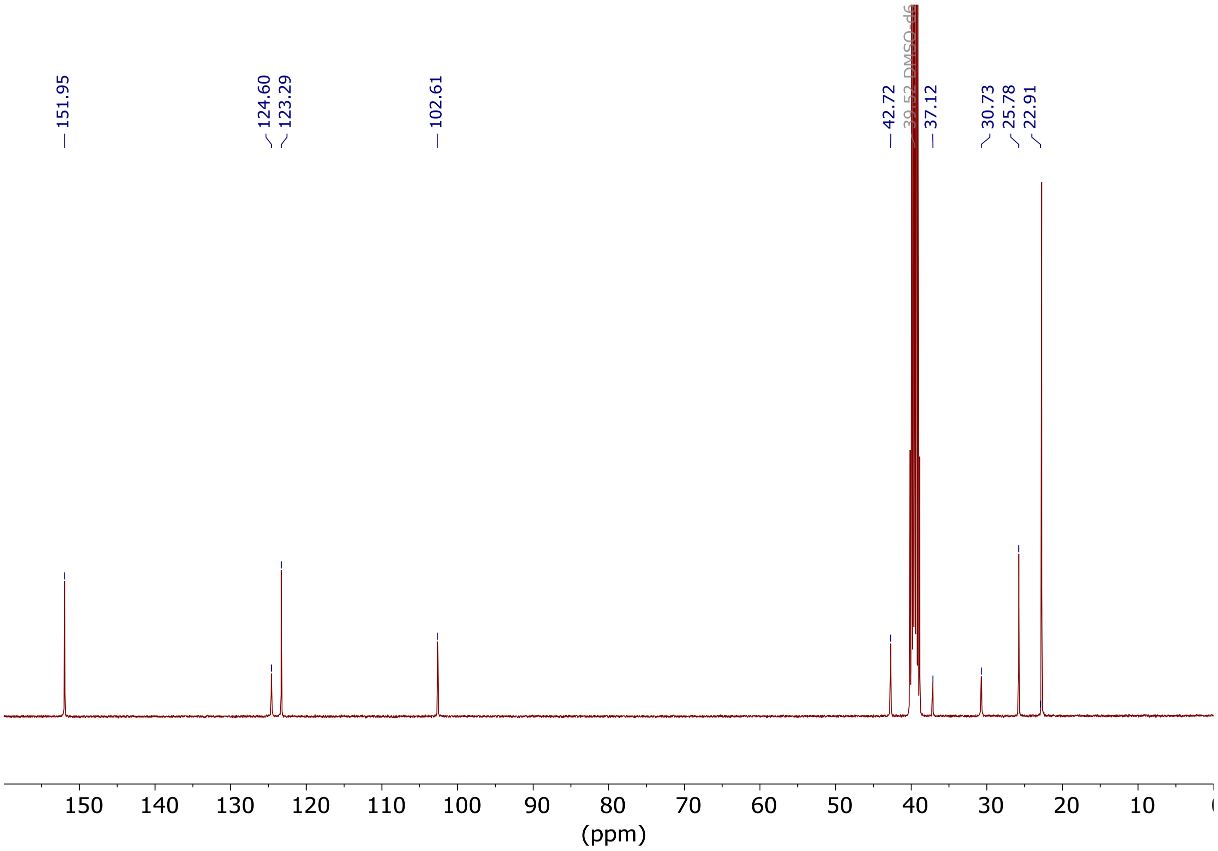

HSQC ^{13}C DMSO-d_6

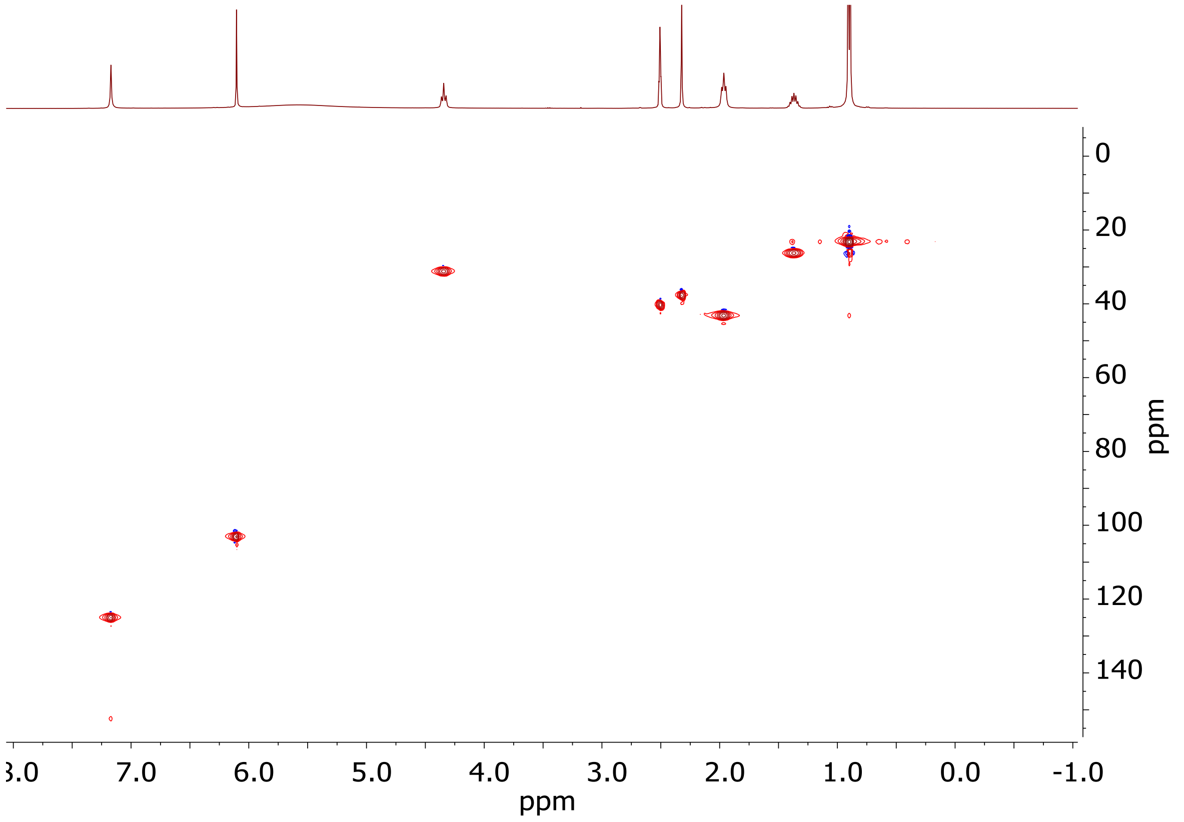

R[4]A:diethylamine

^{1}H NMR in DMSO

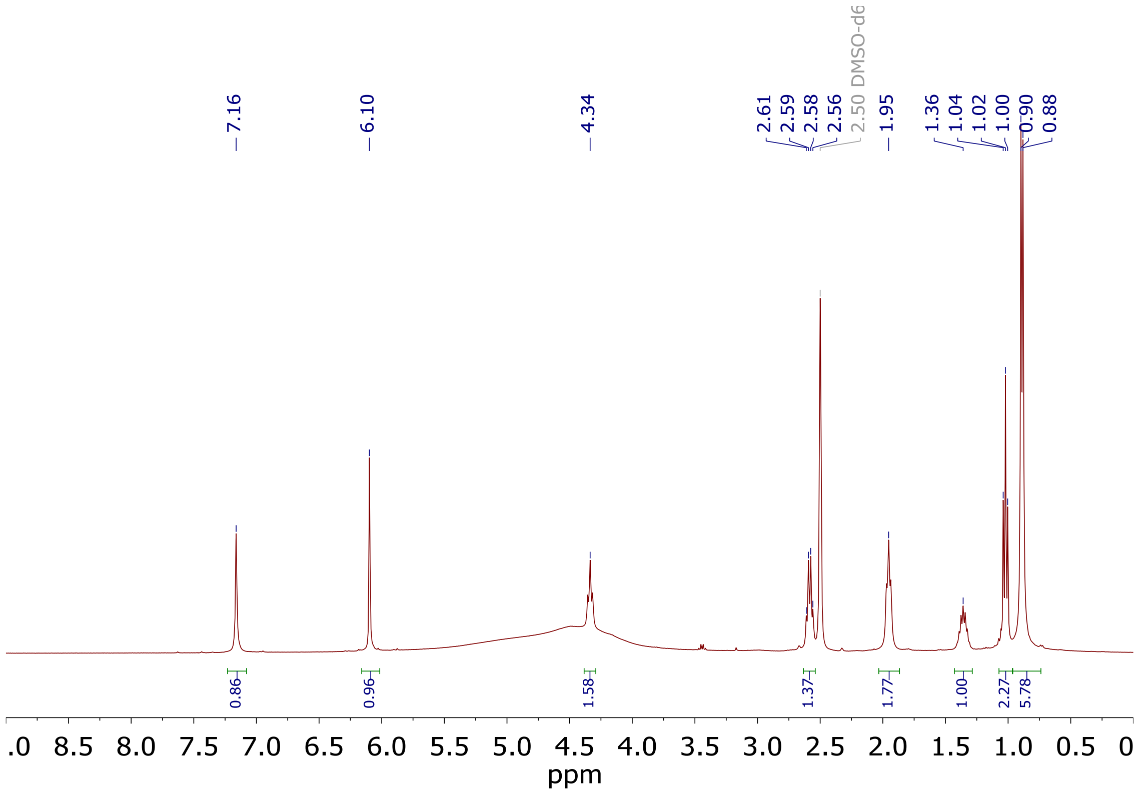

^{1}H NMR in $CDCl_3$

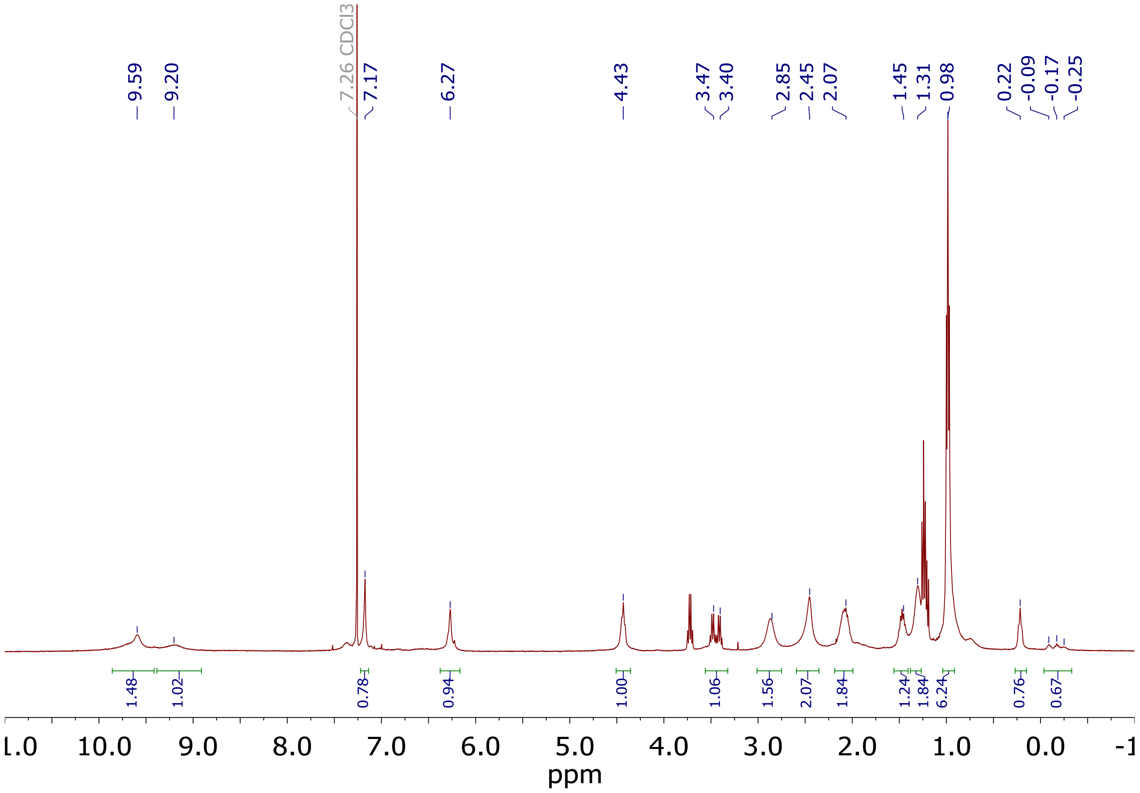

^{13}C NMR in DMSO-d_6

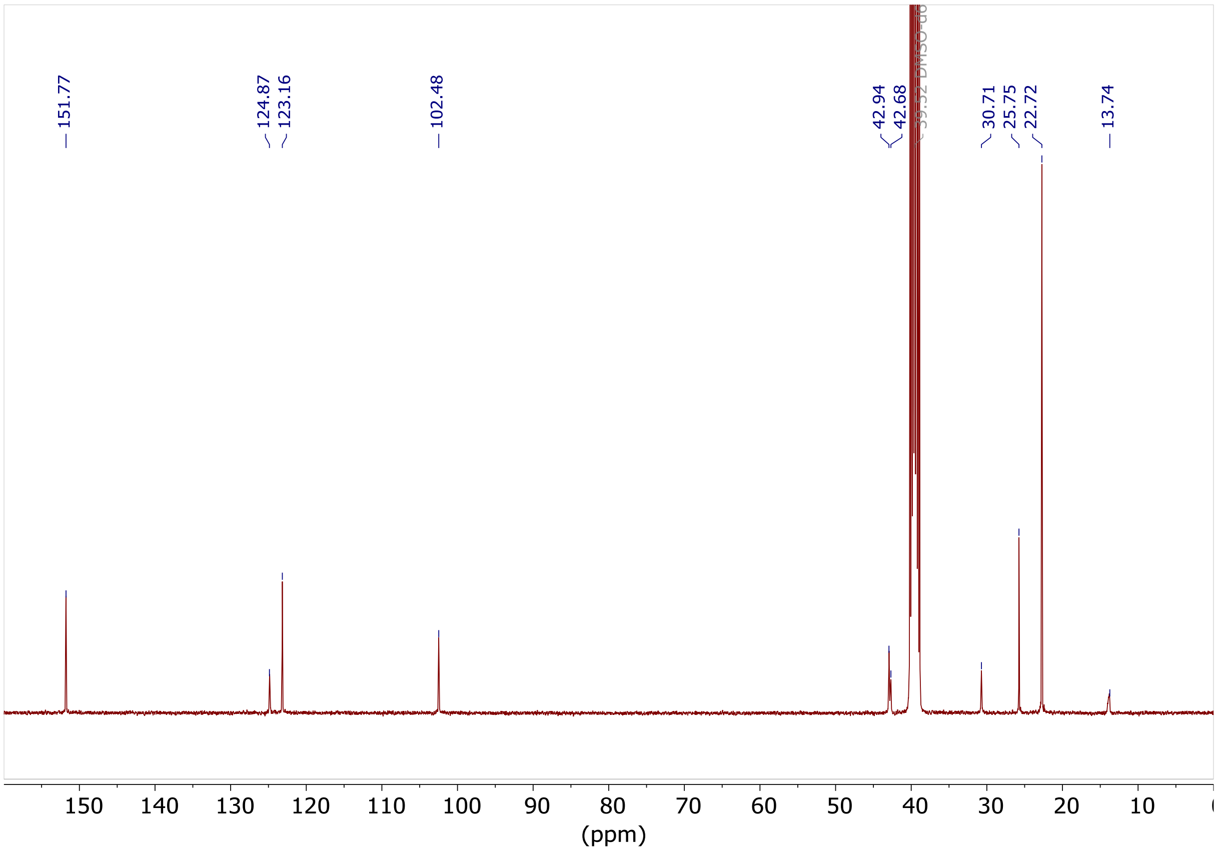

HSQC ^{13}C DMSO-d_6

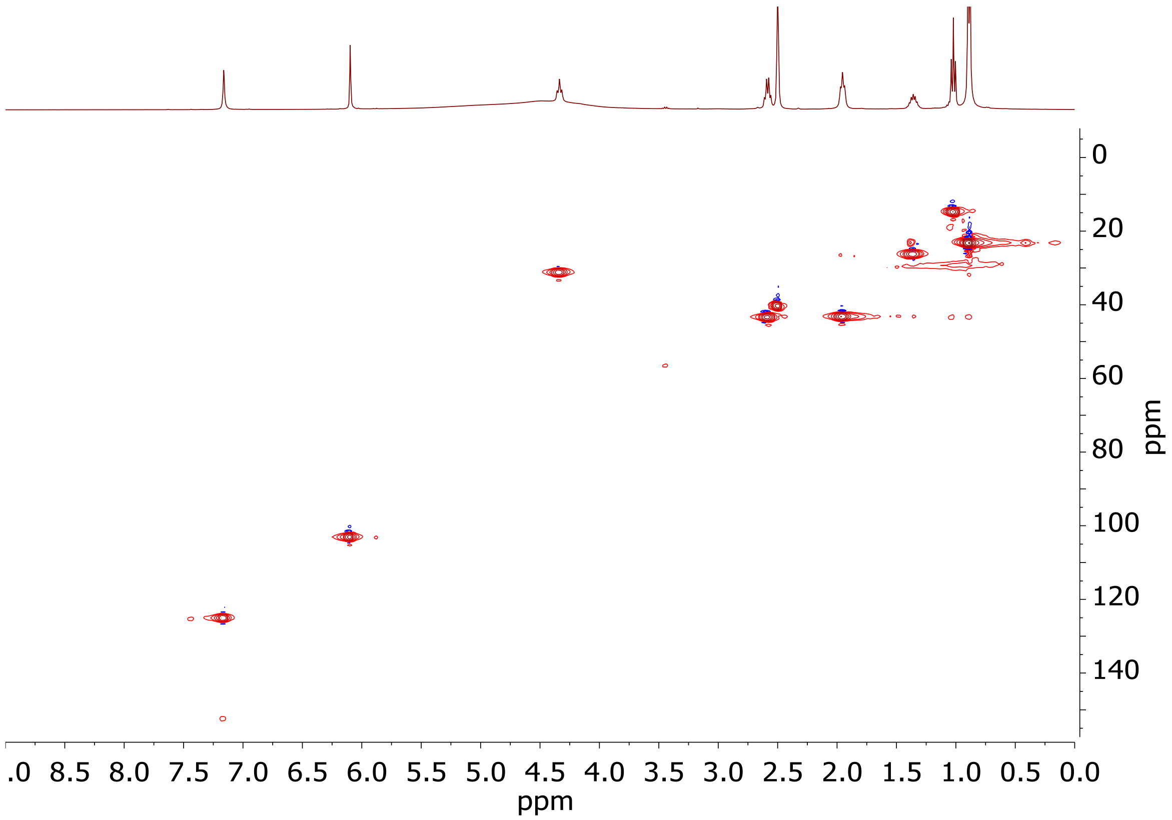

R[4]A:pyrrolidine

^{1}H NMR in DMSO

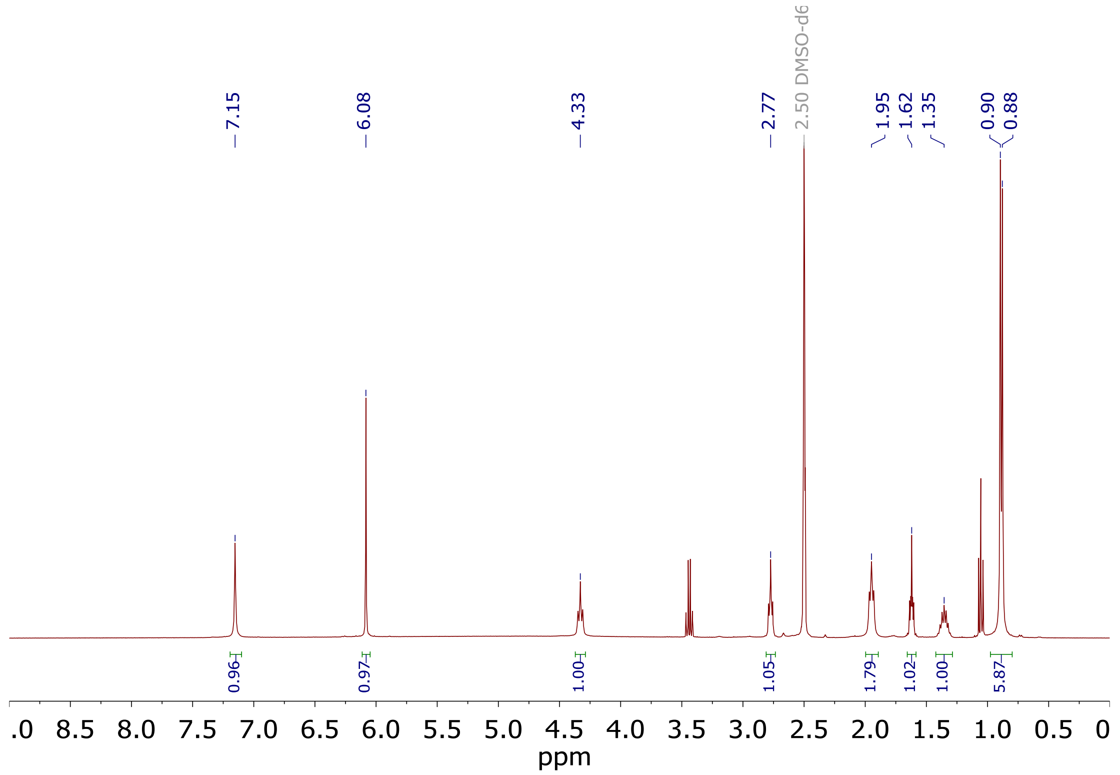

^{13}C NMR DMSO-d_6

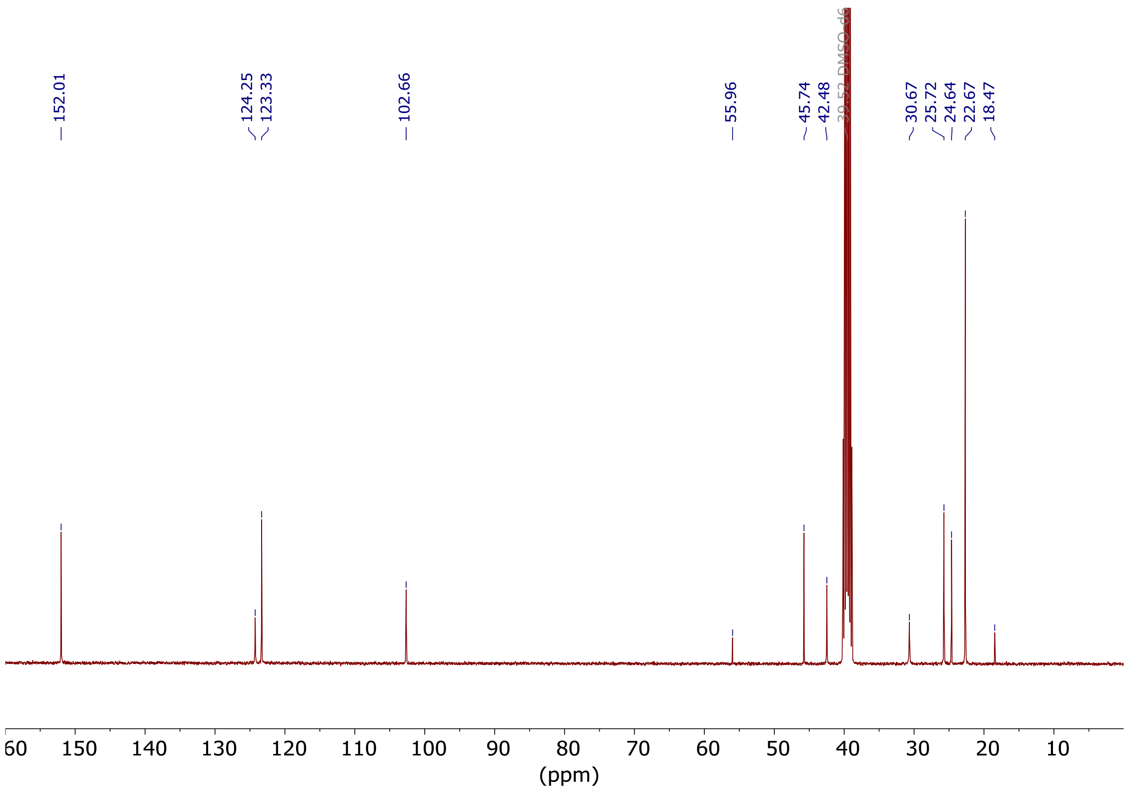

HSQC ^{13}C DMSO-d_6

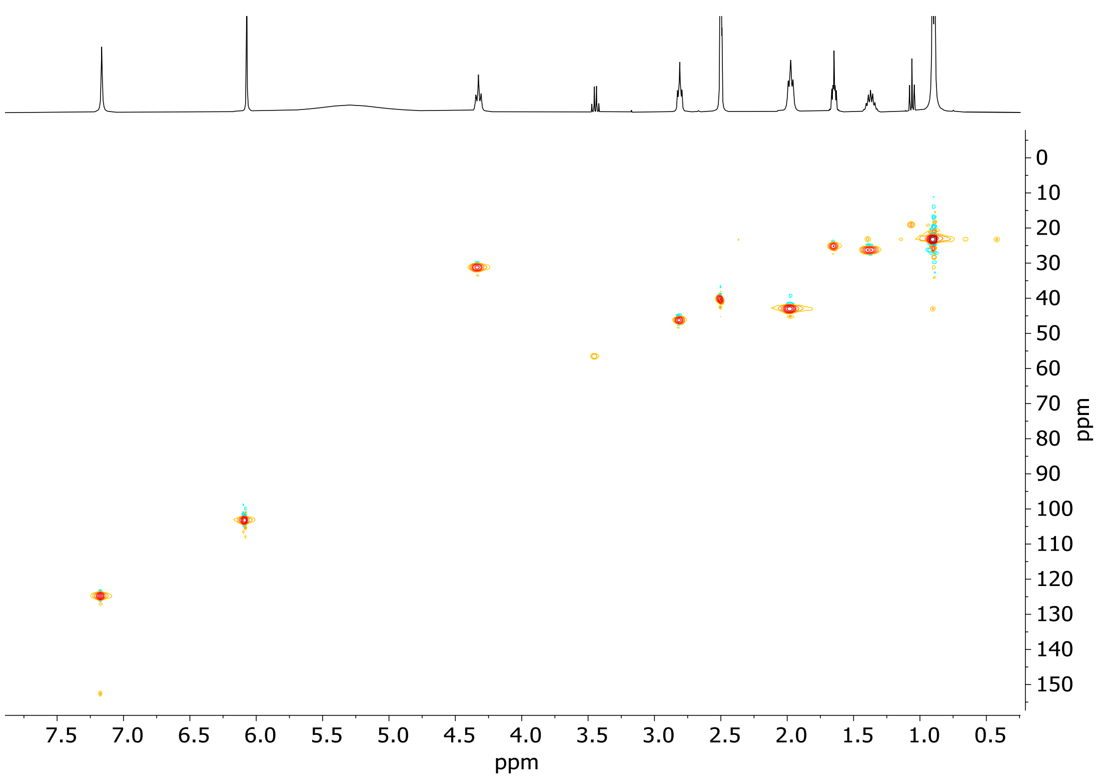

^{1}H NMR in $CDCl_3$

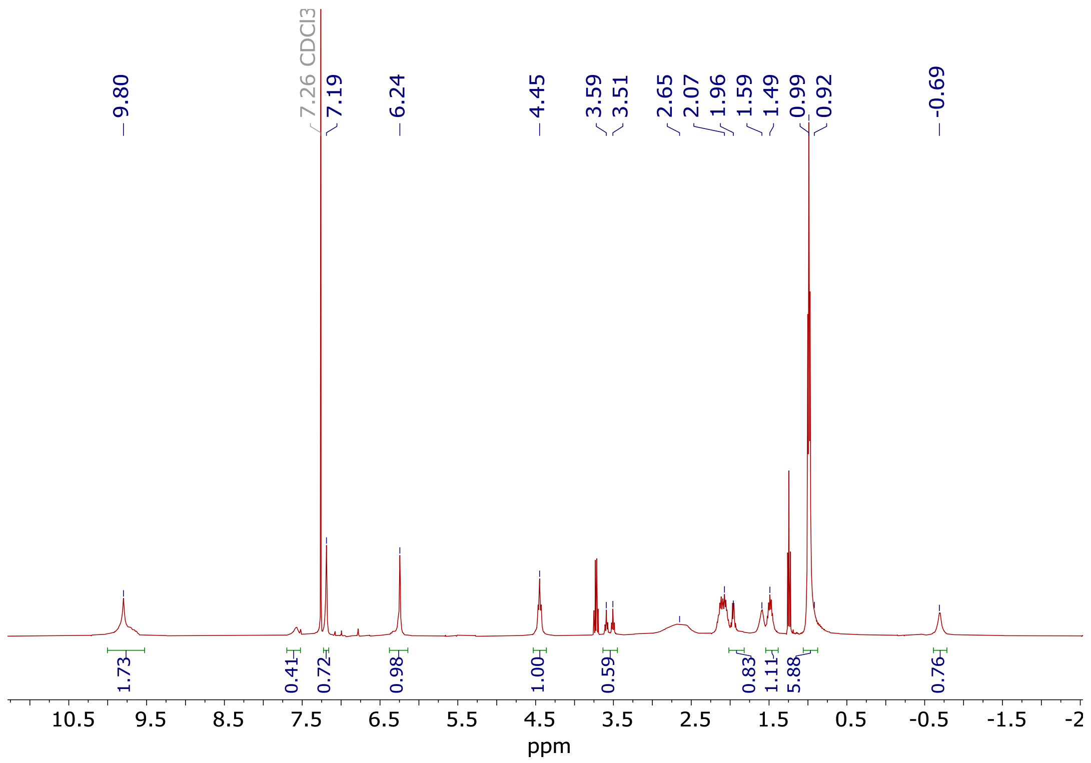

COSY $^{1}H,^{1}H$ – $CDCl_3$

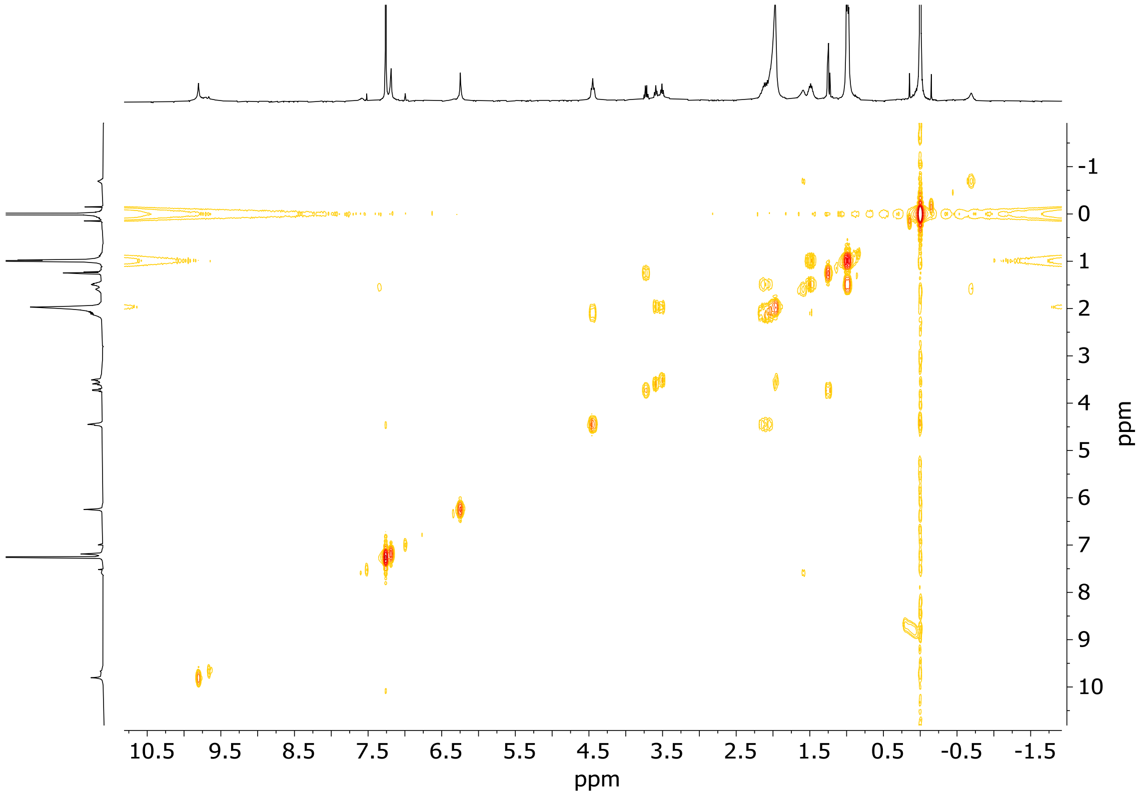

HSQC ^{13}C – $CDCl_3$

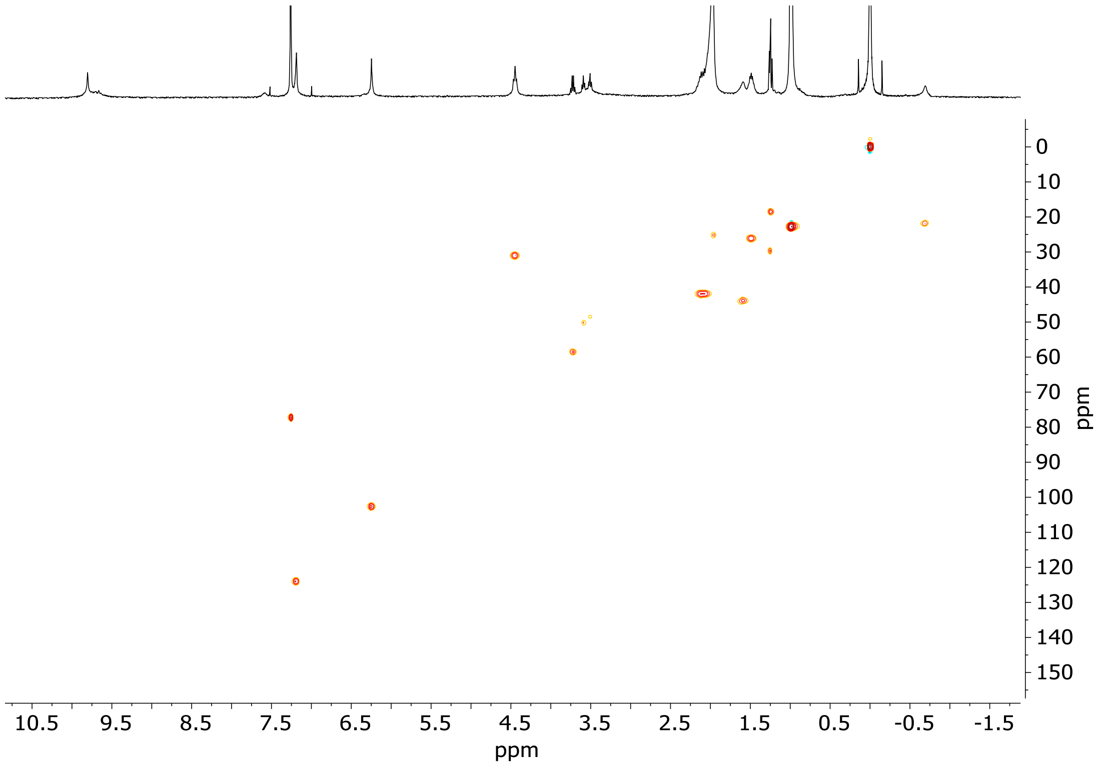

R[4]A:piperidine

^{1}H NMR in DMSO

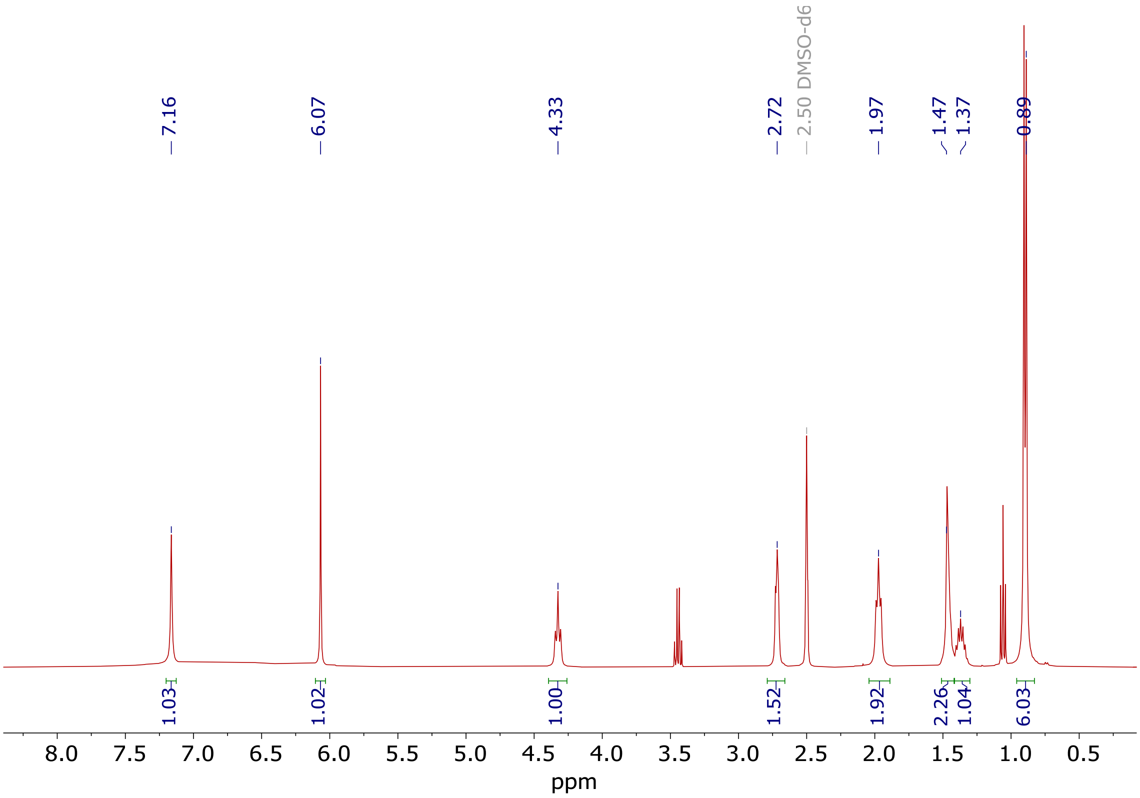

^{1}H NMR in $CDCl_3$

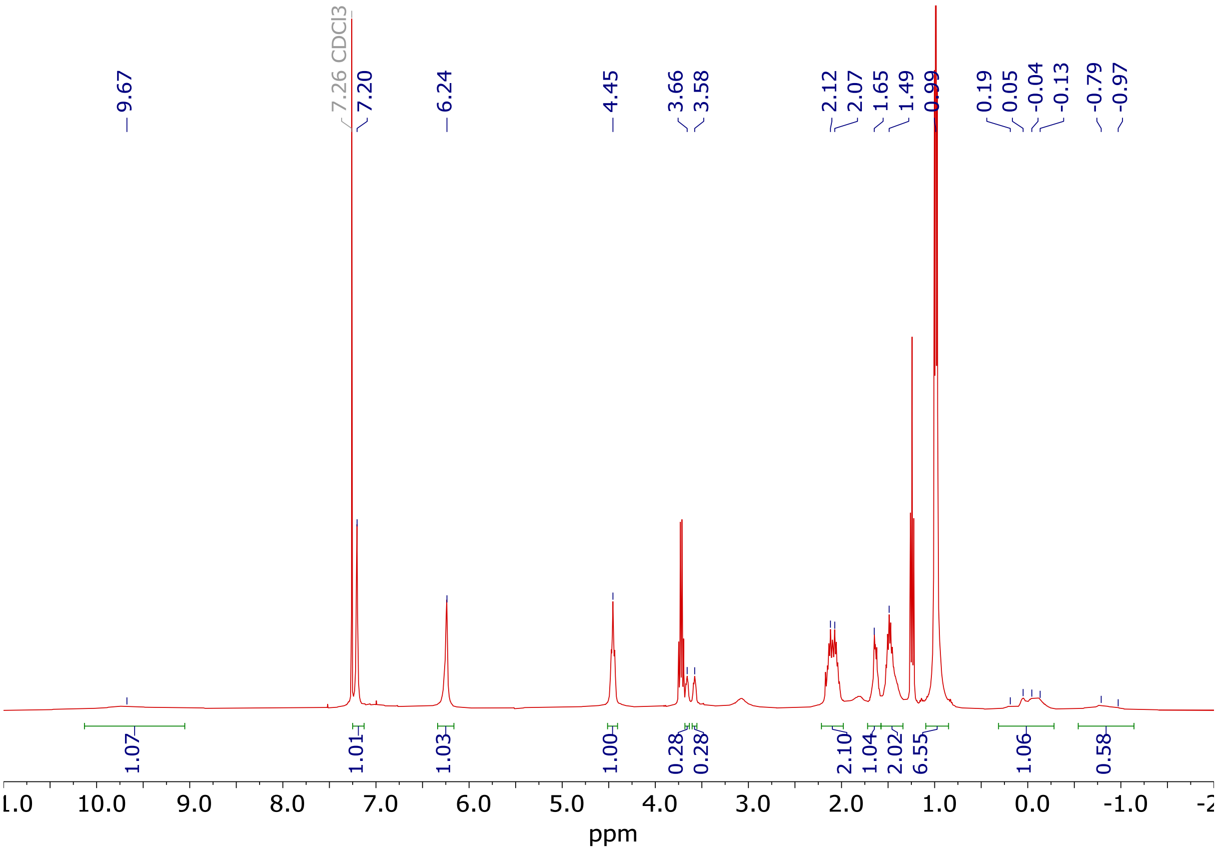

^{13}C NMR in DMSO-d_6

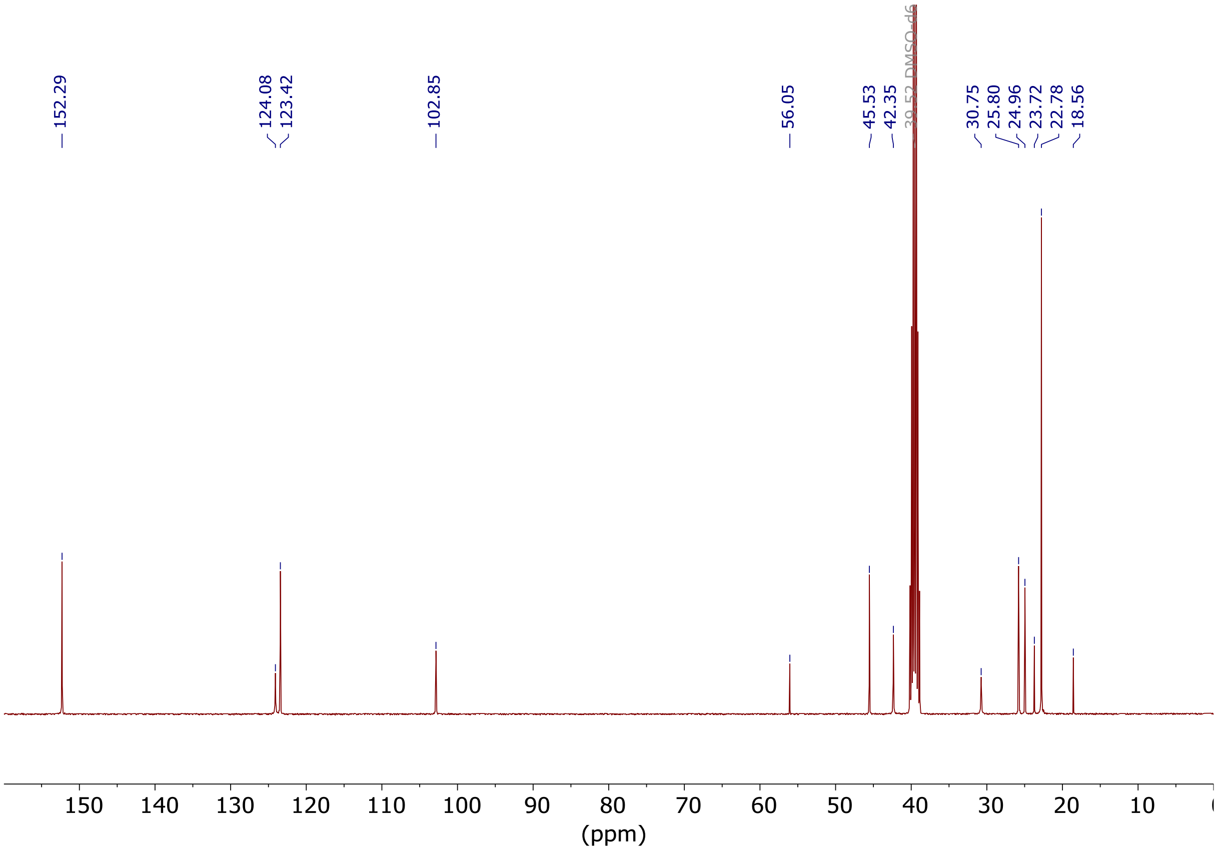

HSQC ^{13}C DMSO-d_6

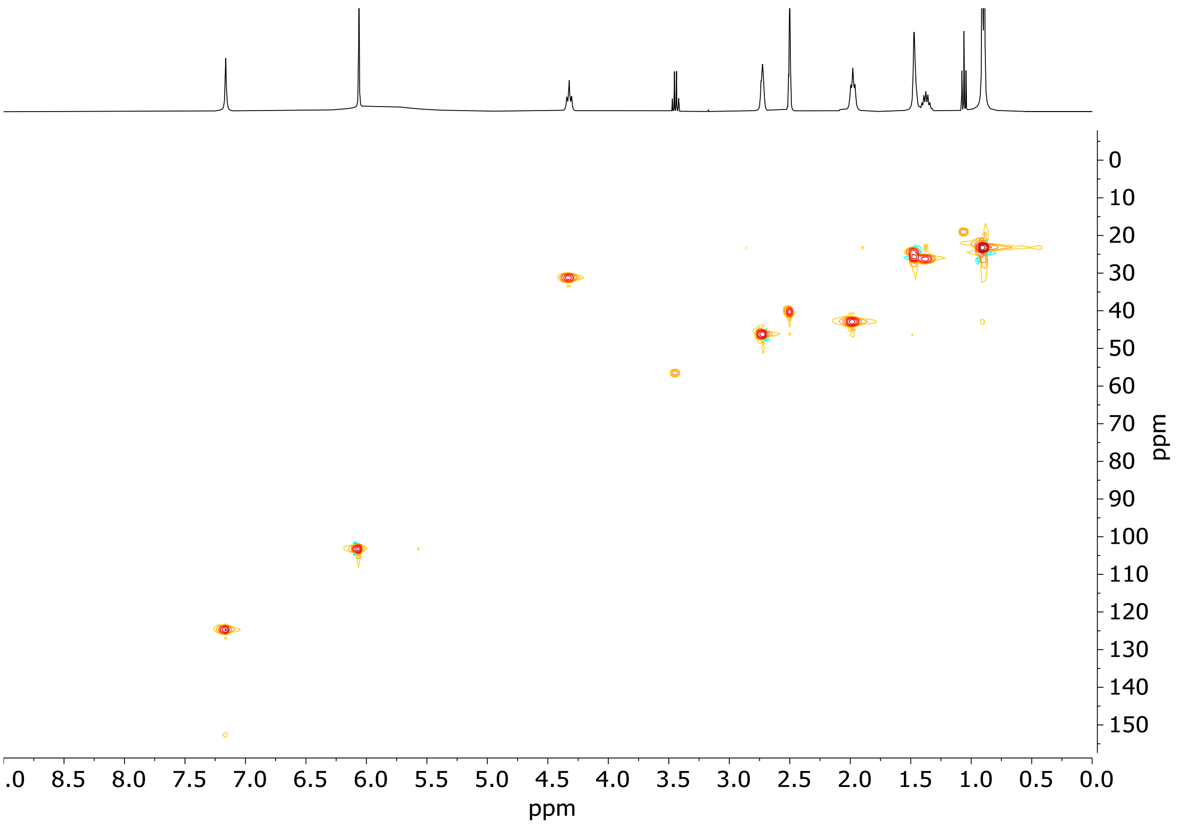

R[4]A:morpholine

^{1}H NMR in DMSO

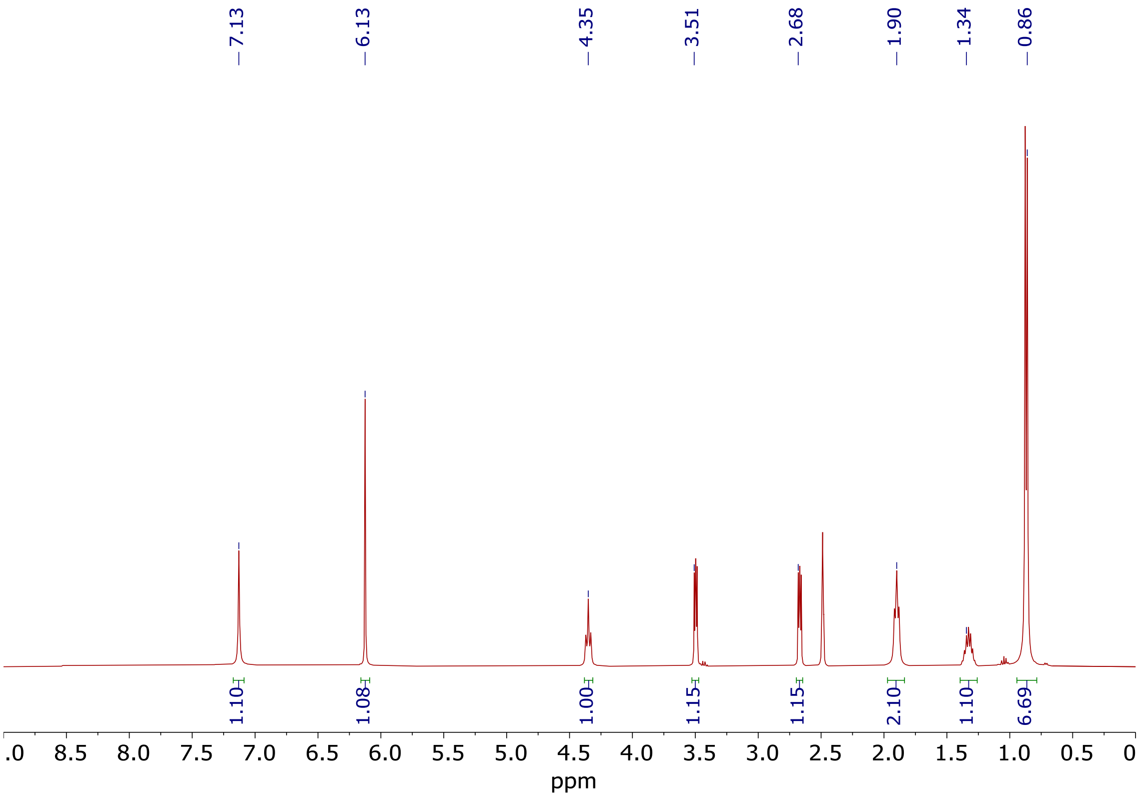

^{1}H NMR in $CDCl_3$

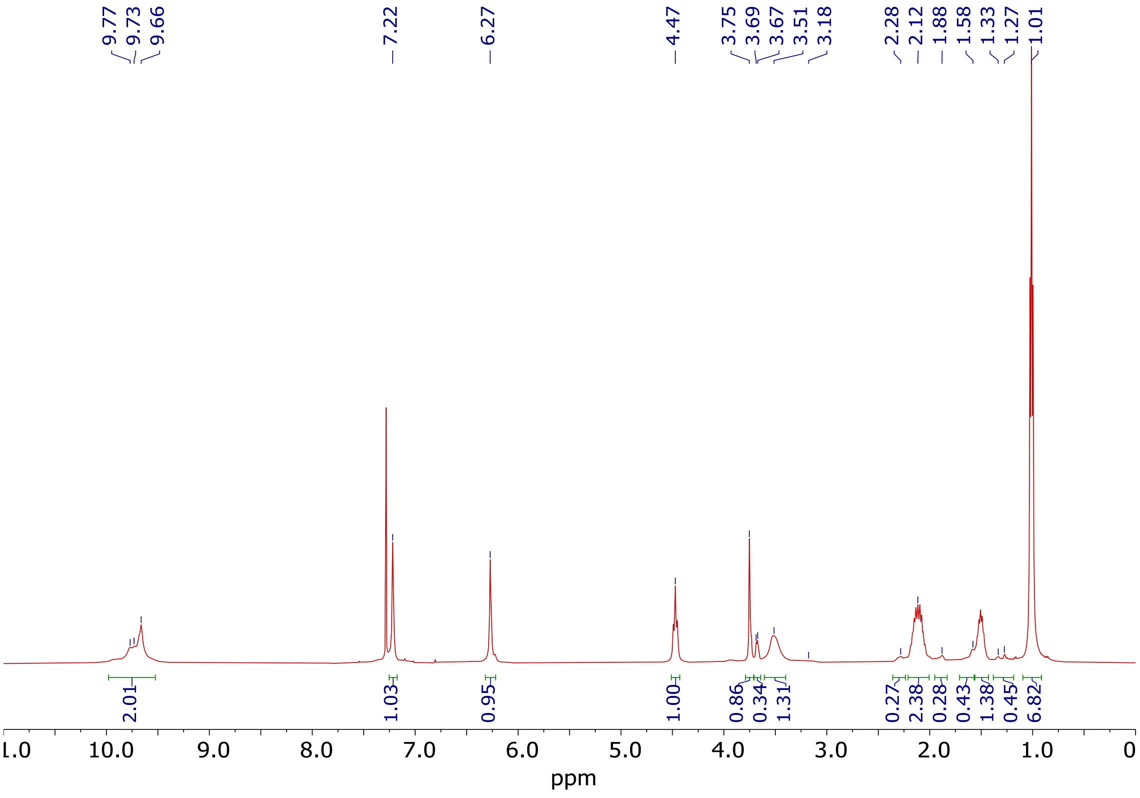

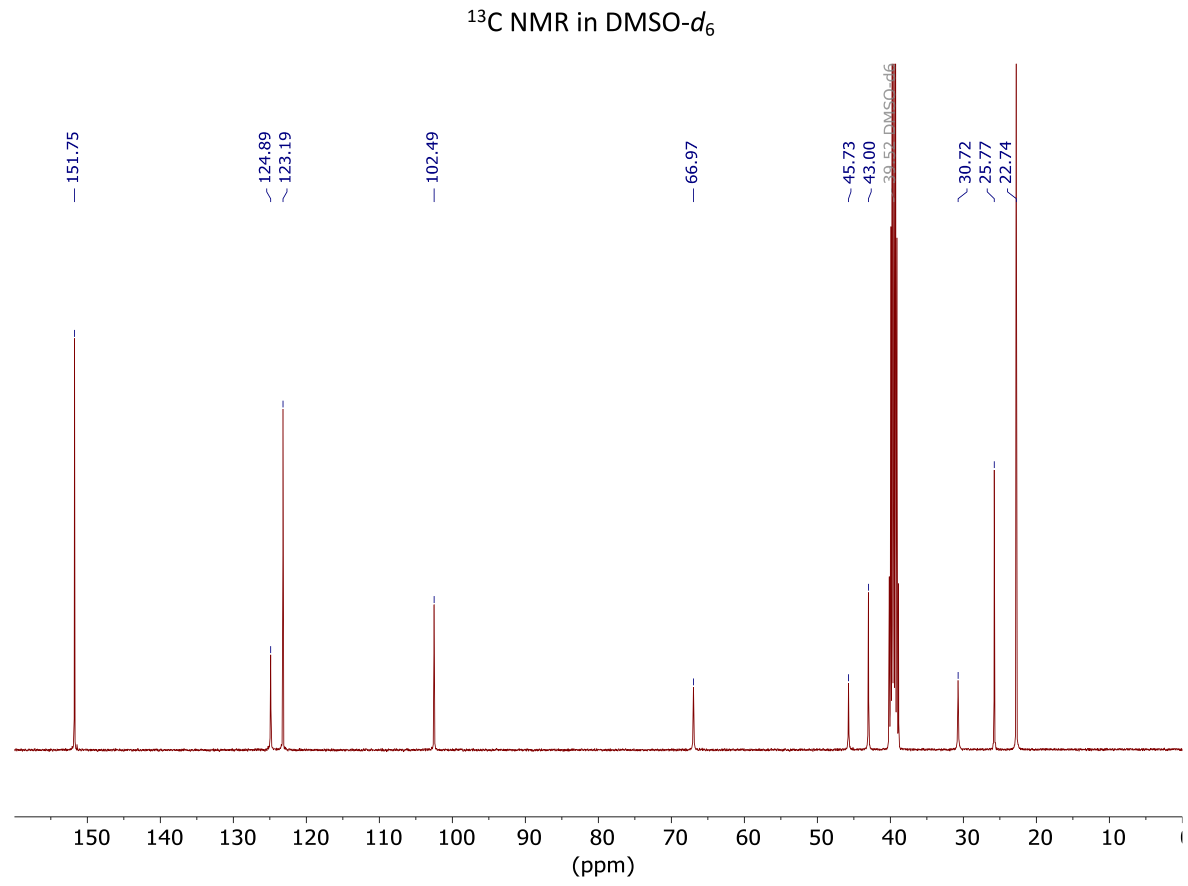
13C NMR in DMSO-d6
151.75
124.89
123.19
102.49
66.97
45.73
43.00
39.52 DMSO-d6
30.72
25.77
22.74
150 140 130 120 110 100 90 80 70 60 50 40 30 20 10
(ppm)

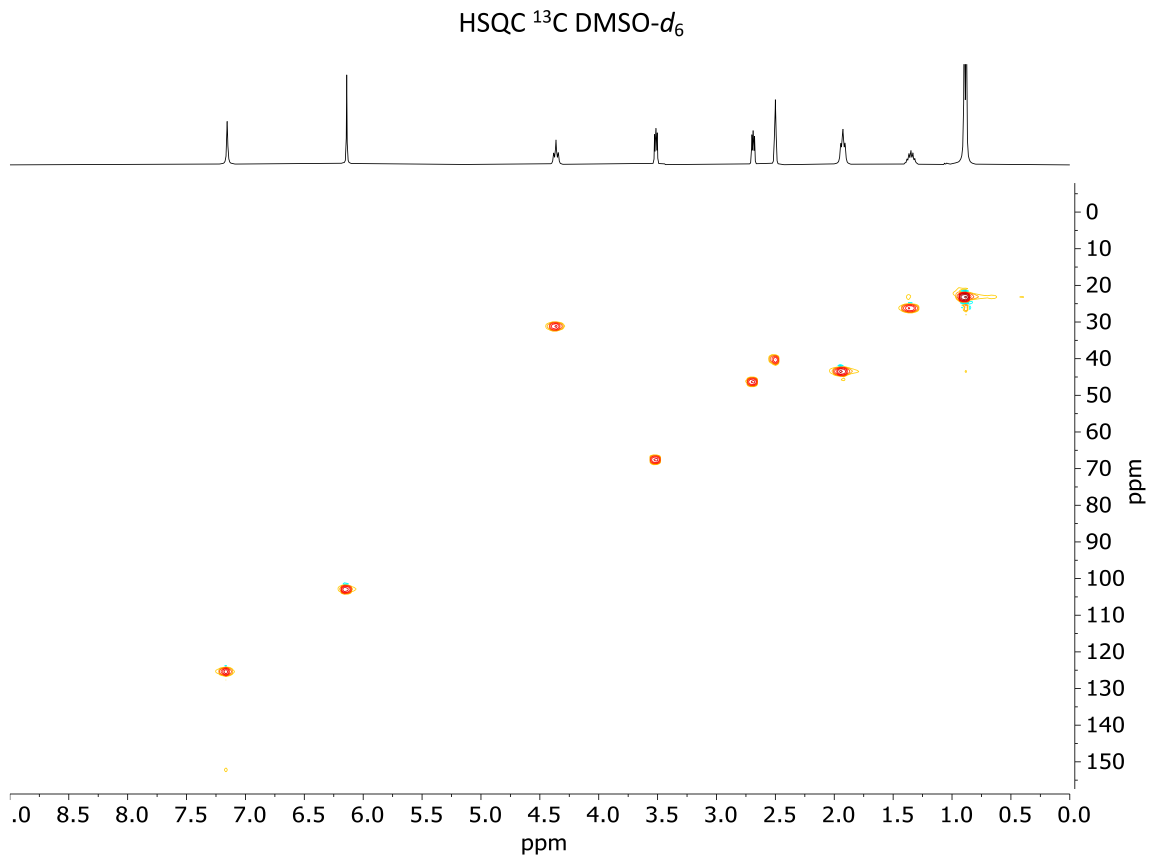
HSQC 13C DMSO-d6
0 10 20 30 40 50 60 70 80 90 100 110 120 130 140 150
ppm
8.5 8.0 7.5 7.0 6.5 6.0 5.5 5.0 4.5 4.0 3.5 3.0 2.5 2.0 1.5 1.0 0.5 0.0
ppm

R[4]A:1-methylpiperazine

^{1}H NMR in DMSO

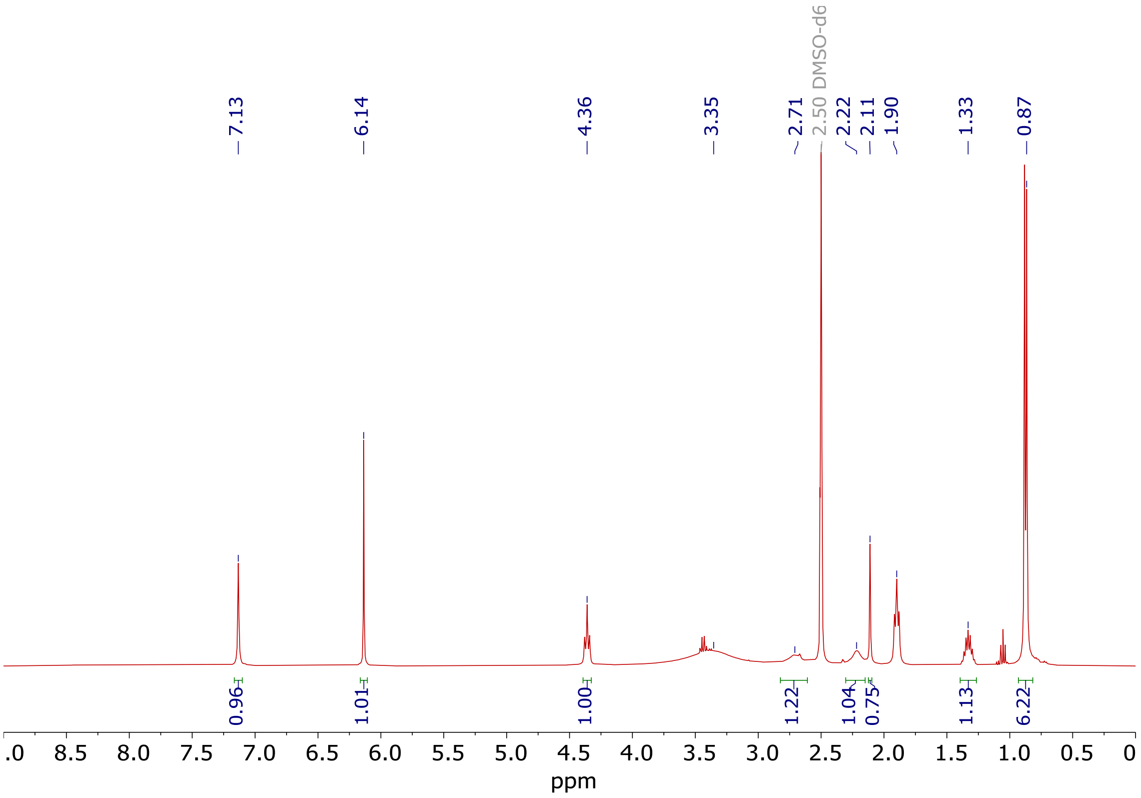

^{1}H NMR in $CDCl_3$

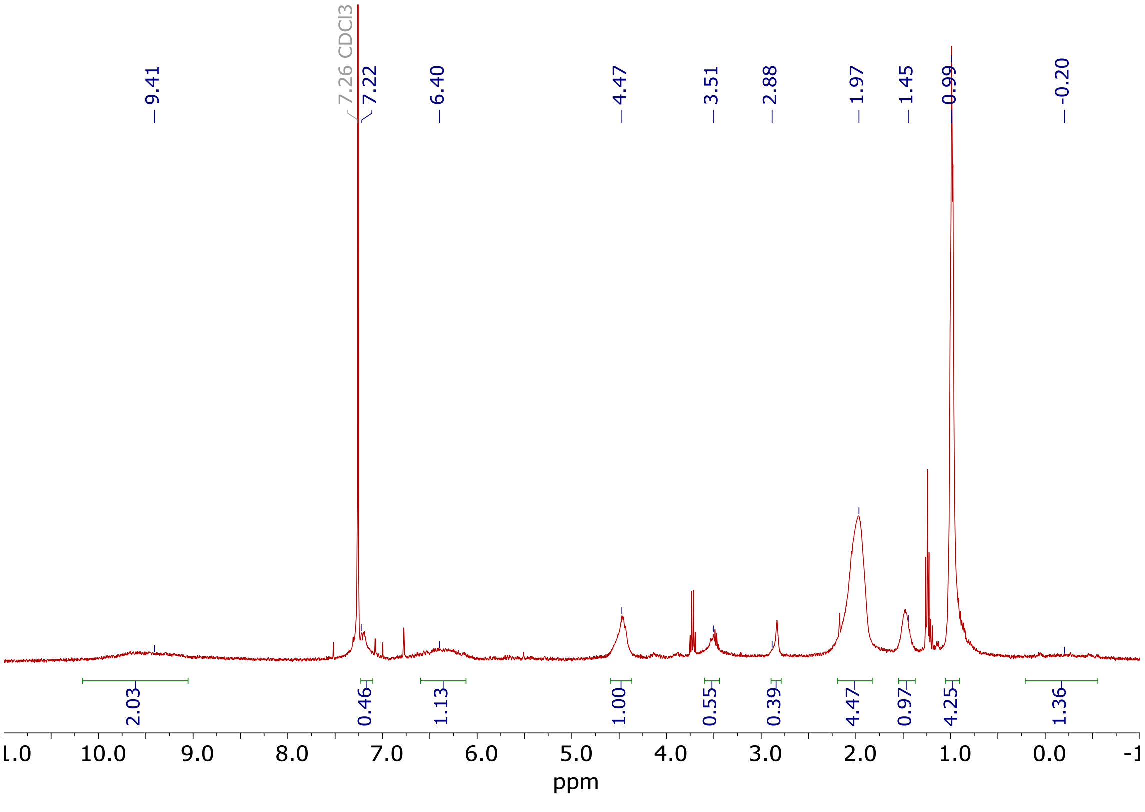

^{13}C NMR in DMSO

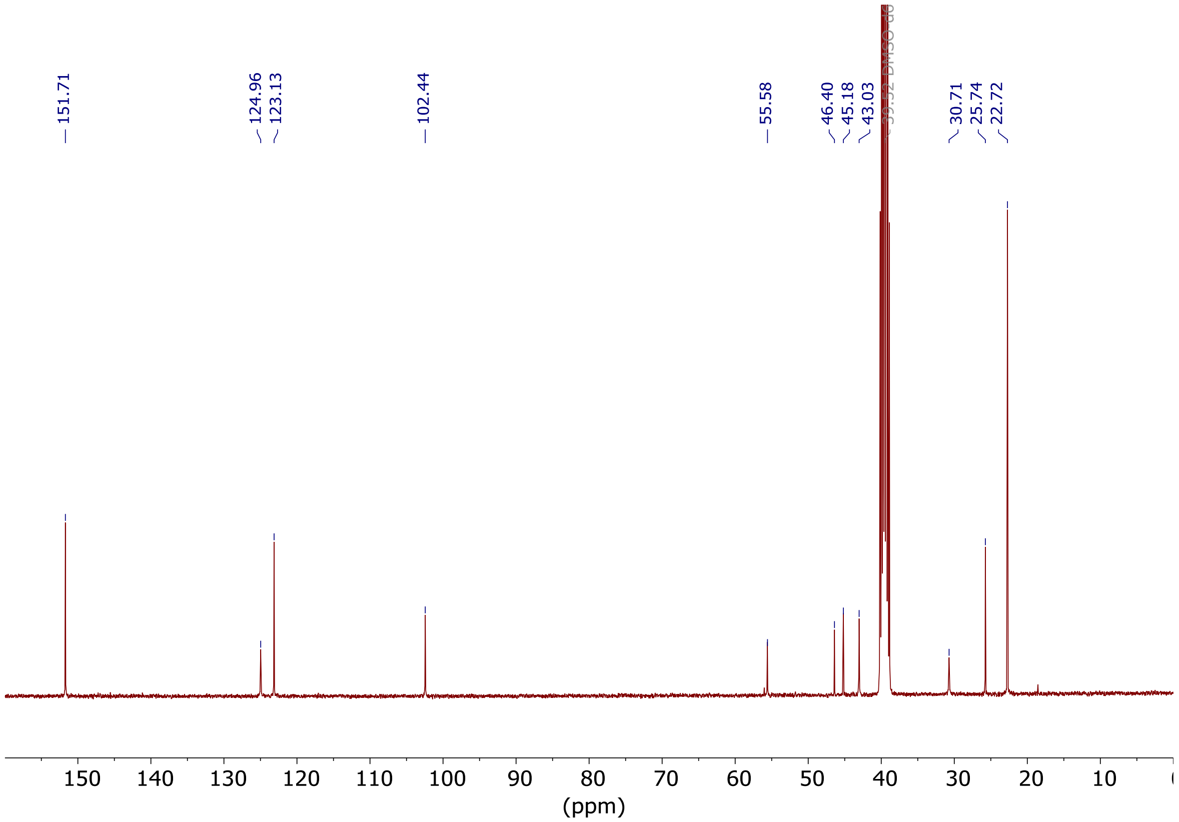

HSQC ^{13}C DMSO-d_6

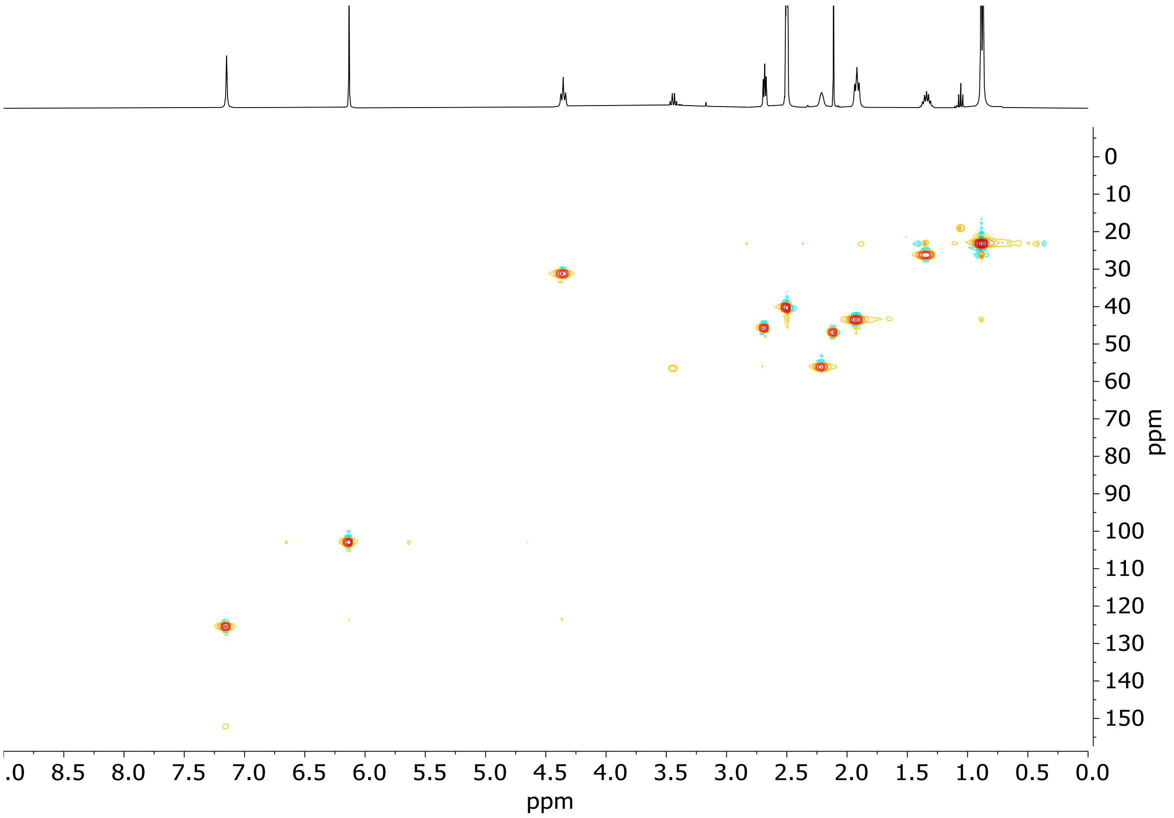

R[4]A:dipropylamine

^{1}H NMR in DMSO

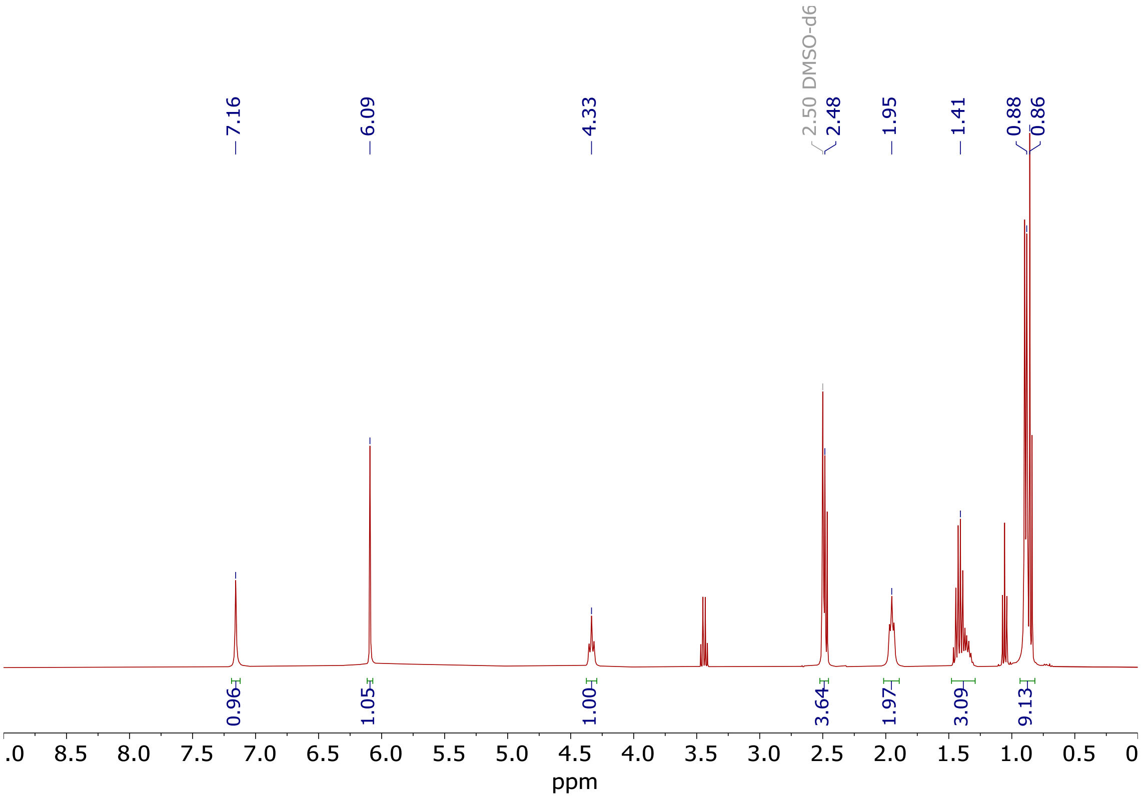

^{13}C NMR in DMSO

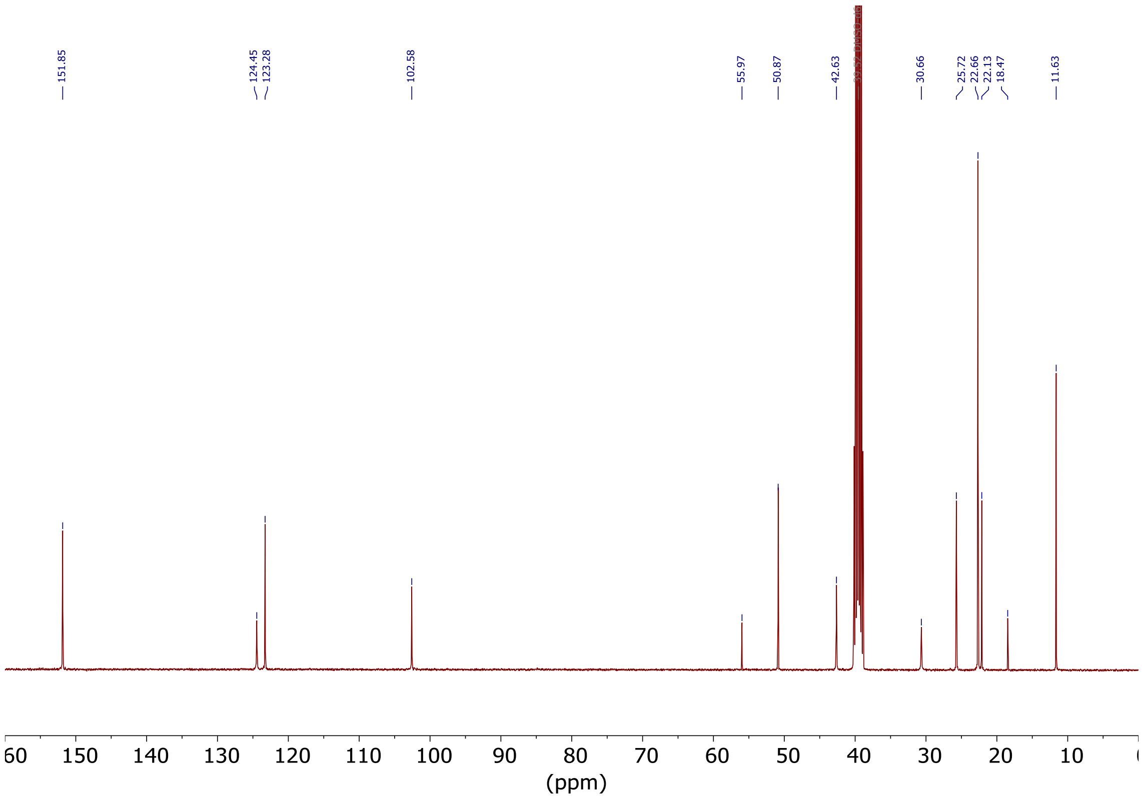

HSQC ^{13}C DMSO-d_6

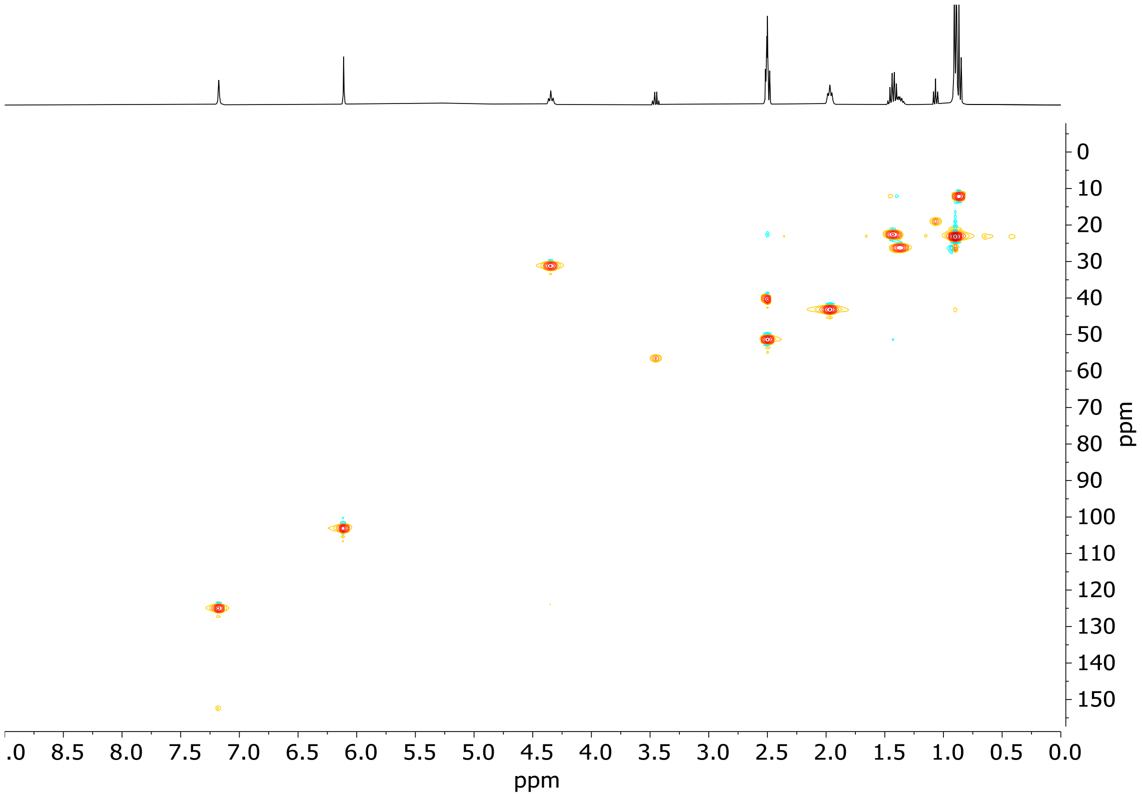

R[4]A:diispropylamine

^{1}H NMR in DMSO

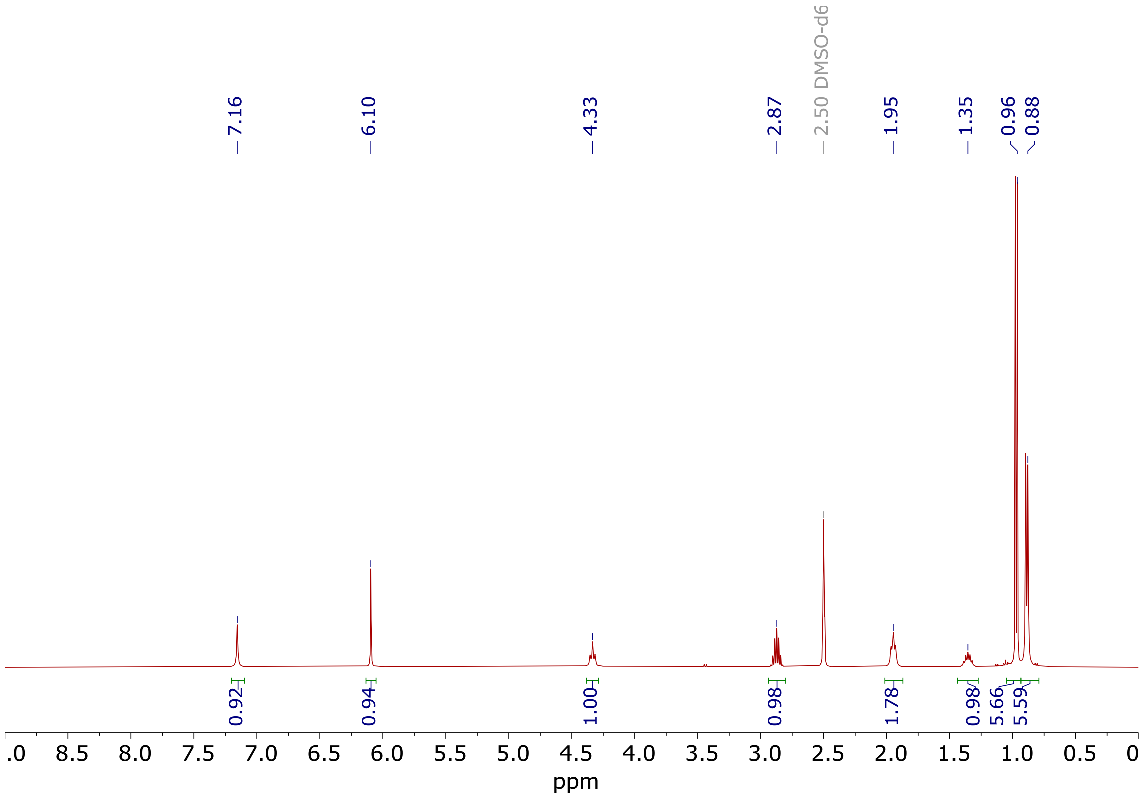

^{13}C NMR in DMSO

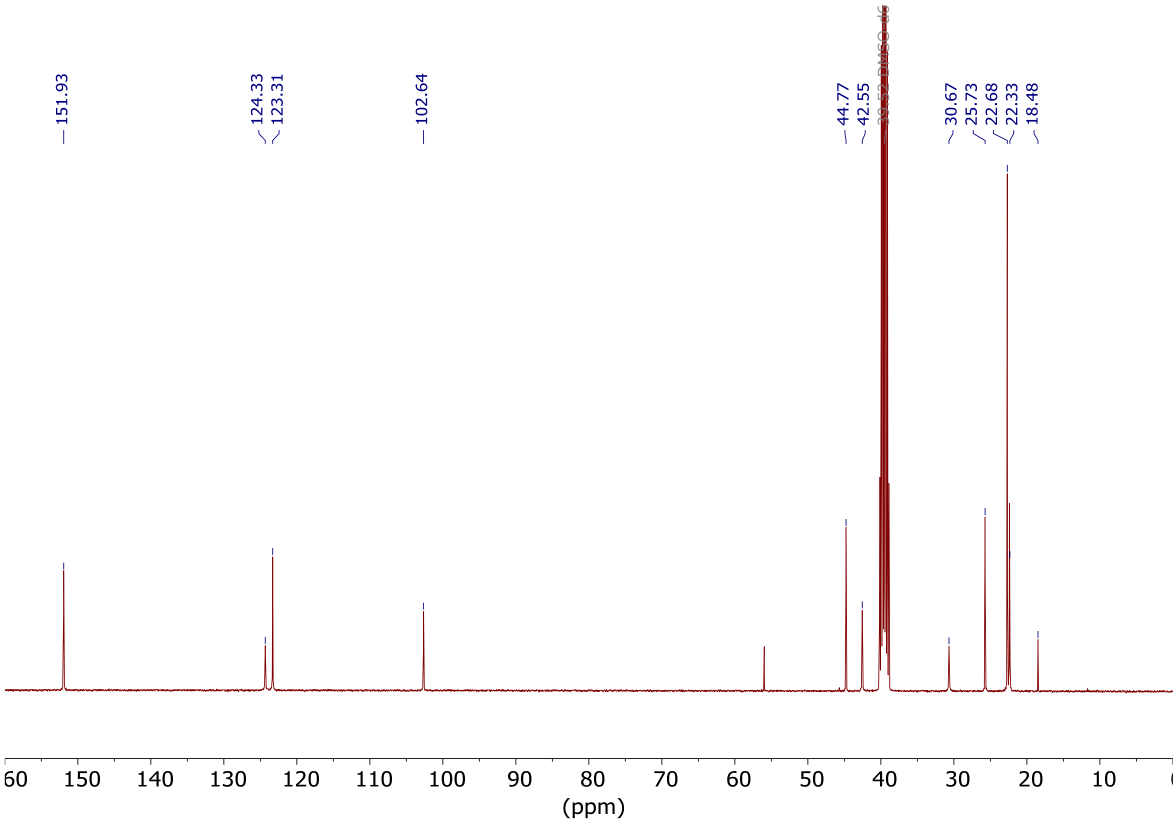

HSQC ^{13}C DMSO-d_6

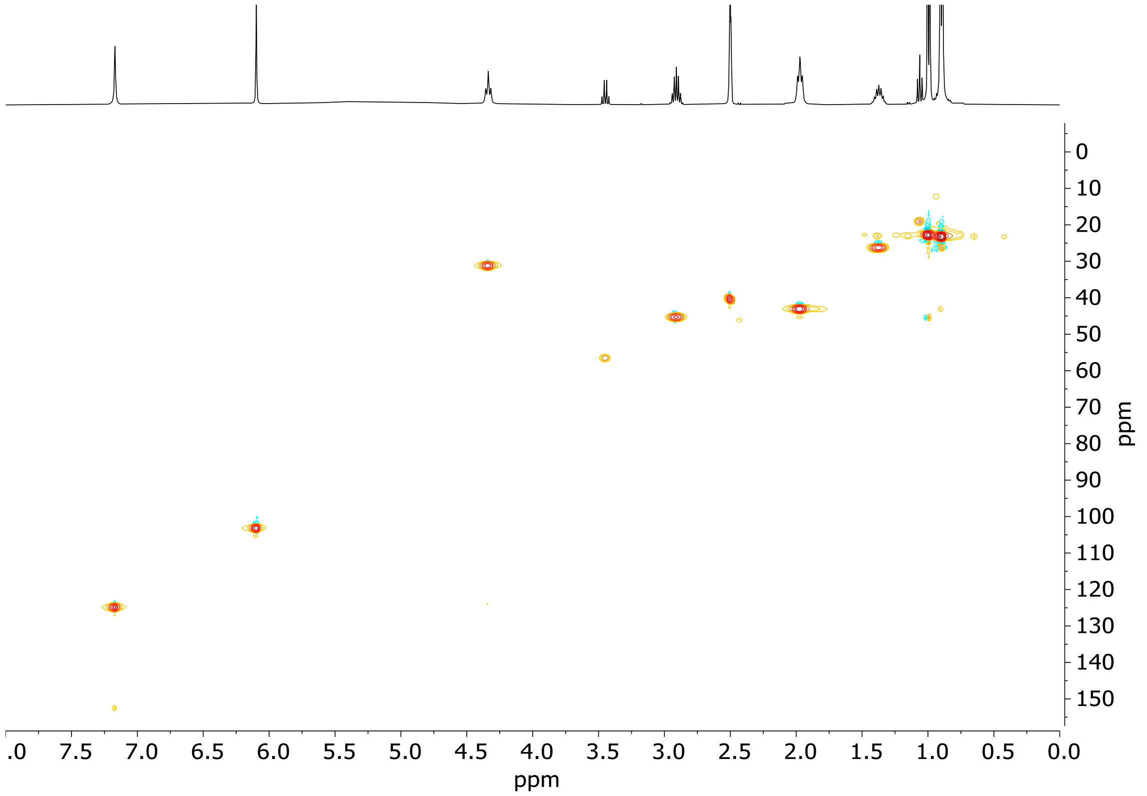

2. The xyz coordinates of the optimized complex structures calculated by the PBE0-D4/mTZVPP method in CHCl3 and DMSO

R[4]A:dimethylamine

$CHCl_3$				DMSO			
C	-2.87994689139466	-1.96761078395866	2.51130567321679	C	-0.33578104570439	-3.26241287341443	0.09857154007913
C	-3.33327358405858	-2.82926851771368	1.51636327078323	C	-1.40350241630552	-3.99560909176605	-0.41149632292248
C	-2.49929449591604	-3.77259179376594	0.94240062398050	C	-1.89885862785128	-3.76810435970710	-1.68464847428640
C	-1.17838308186158	-3.88519445759472	1.36297758861142	C	-1.32514125402258	-2.79399999979684	-2.50001922203530
C	-0.68795440218617	-3.05319735806441	2.37769630547510	C	-0.21439119239064	-2.07082623855414	-2.03813935170798
C	-1.55309840341986	-2.09893980876998	2.89697365415763	C	0.22333914526436	-2.30870525771995	-0.74221996206378
O	-4.64382564073060	-2.70875720595452	1.11913957823504	O	-1.95862875422764	-4.95898644041347	0.39772270365573
O	-0.41978756241288	-4.83472601636849	0.76359196602687	O	-1.85963961018018	-2.60636407558165	-3.72311944189635
C	0.73104541960034	-3.19987166233607	2.89481220719490	C	0.47230233500509	-1.05657575439021	-2.92845060500023
C	1.66490643779023	-2.24222463353148	2.18633285711712	C	-0.16784867484013	0.30747825089076	-2.77666573133251
C	2.27270518384290	-2.61079449930632	0.98258924276005	C	-1.26730771886120	0.65004406314297	-3.59145336203958
C	3.09015620984582	-1.71249916852219	0.30991505885961	C	-1.85359272265289	1.90836467039123	-3.42179315956524
C	3.31352154228917	-0.43622696311608	0.81365557701087	C	-1.38815631730413	2.80892819542797	-2.47105392060778
C	2.74660628993816	-0.04806505175259	2.03229862387539	C	-0.29445149293151	2.48623543139654	-1.66107256898858
C	1.92112951909823	-0.96753040299550	2.67016716204282	C	0.26859435019211	1.22467823256161	-1.83465913666821
O	2.05614856910418	-3.85612624725390	0.48270902603639	O	-1.74040536093806	-0.19629188559200	-4.50552705608141
C	3.02320135441616	1.31580712910084	2.63847938325711	C	0.25954426981118	3.45196378395765	-0.62926056559139
C	2.03369208816187	2.33323647529195	2.10383477969295	C	-0.42691221065590	3.23084654173533	0.70557676952527
C	2.36693865487050	3.16491762311636	1.03752376713843	C	-1.53451709072523	3.99278108077219	1.07334431359772
C	1.46267635485731	4.07310558387632	0.51730865800340	C	-2.18803945443996	3.78395373984032	2.27499741349036
C	0.19378332797785	4.18987753152245	1.07290798777434	C	-1.73286808388044	2.81160942987676	3.15898397872537
C	-0.16795174726520	3.40550770615965	2.17375434219598	C	-0.60536465118146	2.04461768715783	2.84424239251317
C	0.75787958932818	2.47512686925352	2.63099254126617	C	-0.00906766417586	2.26138734196938	1.60671750086672
O	3.63209272183076	3.05222130606735	0.51383709189536	O	-1.95723916960338	4.96402059169611	0.20058504899158
O	-0.63045640919136	5.11295013253116	0.51354697922026	O	-2.41796714520639	2.68922381473274	4.32586212678870
C	-1.50115825550111	3.58934430046066	2.87667345513825	C	-0.02289531044392	1.04352842062174	3.82732553419401
C	-2.52236575638016	2.60016010189015	2.35199003228959	C	-0.60112934522737	-0.33880361146862	3.59187142035020
C	-3.34720810110243	2.93464815775053	1.28032133604904	C	-1.73102086514281	-0.76308144594724	4.28918720867339
C	-4.26438652280345	2.03688180150826	0.76441400714871	C	-2.29441364631040	-2.00699422321690	4.06679081122171
C	-4.39600369560226	0.77174527053341	1.32671163601251	C	-1.72217303876941	-2.87733086833070	3.14515686468123
C	-3.61653822357364	0.40888679715193	2.43030087968991	C	-0.55965172898948	-2.51172690473678	2.45811581320938
C	-2.68045184898447	1.32945068908378	2.88607862976317	C	-0.05656753926756	-1.23455855391326	2.68195685218123
O	-3.22479465148645	4.19575761877145	0.75008306199708	O	-2.27010742517310	0.09640985093315	5.21385979308684
O	-5.32720723442919	-0.04260840320143	0.76867319087284	O	-2.33075726648539	-4.08192828744534	2.98863959208046
C	-3.79975442514137	-0.92920095608369	3.12153823155203	C	0.14474034998875	-3.47641744072064	1.51998558156696
C	0.79621123451164	-3.10460273313095	4.42105503249922	C	1.99035987284698	-1.03733532048271	-2.73658023184872
C	3.10278886812271	1.26345998310705	4.16998848019499	C	1.79250816993132	3.40531159421491	-0.56009153404604
C	-1.33394824468747	3.58728748545865	4.40309893167985	C	1.51175893864998	1.09366115486979	3.84406192320041
C	-3.68227370571005	-0.80443317201324	4.64767857009371	C	1.66925894593948	-3.42245807188964	1.69391050250809
C	2.17360497088000	-3.36262921918738	5.02932733178690	C	2.73346896675988	-0.25587974104299	-3.82120435706590
C	3.89816438785702	2.39180493401784	4.82758243411217	C	2.47004885798044	4.66379103160558	-0.01700112606815
C	-2.41193253657433	4.34236350106340	5.18126675123788	C	2.16730759689107	0.62676108331044	5.14400143158643
C	-4.39687308255135	-1.88938130842426	5.45368309384937	C	2.41692642890124	-4.69653425809917	1.30008444337056
O	4.12880328910239	0.36325913845715	0.07627498215747	O	-2.03442489027032	4.01036355989676	-2.40199499342924
C	2.09561219123070	-3.21260821842443	6.54407309418487	C	4.12536977695785	0.13494174003332	-3.34130263601453
C	2.72828332777968	-4.72996573634815	4.64997296597381	C	2.82180930827978	-1.05033686007069	-5.11945305160018
C	-4.34444050552486	-1.53057308916809	6.93498768432821	C	3.88632319637062	-4.55829032817476	1.68329592158703
C	-3.84265858162033	-3.29219526997720	5.23515104155530	C	2.29120130483431	-5.05652555246607	-0.17528402136152
C	-2.01840238341781	4.40747540039769	6.65277001688991	C	3.66720981369629	0.89422782921274	5.07600186412204
C	-3.81017132729050	3.75345322461101	5.03577597293486	C	1.91326289323941	-0.83855897596797	5.47737381363267
C	4.06159082760973	2.09070369792009	6.31360425121224	C	3.98217069766405	4.51368834944563	-0.14424222527671
C	3.28434401969439	3.77443583330424	4.63948380054797	C	2.10006998840641	4.99735492016271	1.42317121938980
H	-1.17750565253937	-1.43388334449725	3.66620377328599	H	1.06425533036118	-1.73138055942321	-0.37539907886701
H	-4.82513673897714	-3.30911122878060	0.38696544381769	H	-2.72539481641821	-5.35261796872617	-0.03559408681282
H	0.52505094078757	-4.54884789743816	0.76399327958873	H	-1.73700505049344	-1.64190594836768	-4.04370429797761
H	1.05486863377796	-4.21194149481848	2.63974093449740	H	0.28301475023084	-1.37235926021941	-3.95945538137626
H	1.46167606997207	-0.67607092734762	3.60802651989738	H	1.11187051206134	0.95765129979502	-1.20661044734917
H	2.06706113551170	-3.85286991440141	-0.56225673029744	H	-1.06985243386431	0.06088375898459	-5.77323094309105
H	4.02029733357752	1.62168243160946	2.30405594877753	H	0.00986111039036	4.46520862458633	-0.96098095604871
H	0.47687964657996	1.84863074568054	3.47091097008754	H	0.85455223737932	1.65893195467701	1.34688411125227
H	3.72442449128933	3.63084044567360	-0.25166546015354	H	-2.75366590892627	5.39304899831631	0.53622414447847
H	-1.56390382514126	4.86289800935188	0.65030256065385	H	-2.30935146200752	1.79352620558950	4.70148279946373
H	-1.85992580420495	4.59233708733905	2.62443038699378	H	-0.33555669960532	1.36098170621057	4.82764826045820
H	-2.06376077556751	1.04743957441442	3.73169053203486	H	0.83301974335976	-0.93759018893405	2.13748564961649
H	-3.81015731879940	4.29368521986148	-0.00937641837944	H	-3.07428150393168	-0.28001163077100	5.59153813614464
H	-5.13912267546311	-0.98059351937400	0.96145288606927	H	-2.13782706141619	-4.45536648601965	2.10627158861632
H	-4.82928291201431	-1.25011481953834	2.93281727877066	H	-0.14854342447512	-4.48782884687206	1.81963840846336

H	0.08566050140734	-3.83402932777990	4.83012338251824
H	0.44887673808557	-2.12432414085345	4.76357855905558
H	3.58447265591203	0.31710077022550	4.43882492547408
H	2.10236819356478	1.22919982697647	4.61661839110280
H	-0.37173197500281	4.06121584790928	4.62477304221715
H	-1.25816462225546	2.56577540514349	4.79204228370883
H	-4.11780259876595	0.15997996820172	4.93066863096848
H	-2.63260187542417	-0.76357915159987	4.95944395805740
H	2.86307216217576	-2.59944481042263	4.64788125589118
H	4.89747719132420	2.39879389373296	4.37064968716375
H	-2.43557315876393	5.36925272142253	4.79067771691238
H	-5.45044219137359	-1.88788350122417	5.14147110288576
H	4.01478712361768	1.30404509719680	0.30553899093040
H	1.71467466732741	-2.22595716367973	6.82544806573592
H	1.42504105153500	-3.96496249395155	6.97396040781825
H	3.07890466776693	-3.33933994217973	7.00605956501478
H	2.02912668448664	-5.52422505911872	4.93480641096769
H	2.91360047599817	-4.81380903118214	3.57592923615452
H	3.67617454884255	-4.91932701394529	5.16197619079015
H	-3.30787324067583	-1.51760136899405	7.28961617415610
H	-4.77456472136987	-0.54239574106693	7.12330003019045
H	-4.89306325333532	-2.25998232440122	7.53758182787529
H	-3.94932918363361	-3.62266457414763	4.20038494421033
H	-2.77706364305150	-3.33241193977013	5.48582634865982
H	-4.36520656136752	-4.01009517962877	5.87448994470006
H	-1.98352707834668	3.40165112731939	7.08590278927731
H	-1.03193321455596	4.86224039166491	6.78283062945403
H	-2.74045488754048	4.99282174359907	7.22913267704559
H	-4.16008104472193	3.77259129836180	4.00193434587368
H	-3.82913076950025	2.71071721077074	5.37078082473734
H	-4.52424752150086	4.31545503564424	5.64514844827914
H	3.08571156583893	2.07817312559336	6.81158261516001
H	4.53308725342390	1.11689031538319	6.47545023989500
H	4.67556371152313	2.85093406053641	6.80486371175597
H	3.24321793272753	4.07008101801677	3.58964882437961
H	2.26083589481013	3.79996569685234	5.02922747498444
H	3.86859428399450	4.52572554148359	5.17935192867922
H	-2.86094472229882	-4.42950988444746	0.15727339043780
H	3.57321118217118	-1.99854284791499	-0.61724714999057
H	1.72875637005416	4.70062936244786	-0.32765329887654
H	-4.88988385322525	2.30606405856608	-0.08110767922979
N	1.77053593570723	-3.90613797093559	-2.09198750197819
C	2.89113234338114	-3.61929549018655	-2.98141918665644
C	0.62916159722827	-3.02390096089563	-2.32537193455765
H	3.24447442545647	-2.60159009865442	-2.80119389673359
H	2.61167142637242	-3.70072392234098	-4.03930770570024
H	0.92141492362093	-1.99560389105642	-2.09965555780573
H	-0.18786143777288	-3.30358375323680	-1.65748782515978
H	1.47445526319606	-4.86624199670005	-2.24130916039383
H	3.70977547956113	-4.31166466444912	-2.77699566881905
H	0.27623767566079	-3.06630115546266	-3.36302715444211
H	2.35782801069892	-2.07099641874378	-2.72566163439496
H	2.24560040698016	-0.61890905518572	-1.75793745436258
H	2.16005446589251	3.24459373331474	-1.57931894585693
H	2.13509334344405	2.54109335651926	0.02000808861156
H	1.80648894095498	2.13572453239165	3.67998571561344
H	1.93428762608084	0.52757661312481	3.00680299786628
H	1.87284376289644	-3.23332885955502	2.75340365439308
H	2.09680491038525	-2.57291837857946	1.15039679523948
H	2.16987159784491	0.66560993097236	-4.01692040880453
H	2.15621925703061	5.50471270292720	-0.65094082330854
H	1.75023611711857	1.23653125017085	5.95755932088353
H	1.98897361051841	-5.52070554948596	1.88769394270820
H	-1.94042929241513	4.40422219648831	-1.51486850550542
H	4.07213809211746	0.76808671628130	-2.45012709039156
H	4.70912572881541	-0.75641763590101	-3.08541537880954
H	4.67373069824376	0.68224731423256	-4.11390801710793
H	3.44393736757453	-1.94096899873587	-4.97770979004591
H	1.83939070129494	-1.38617552388484	-5.46324842806266
H	3.26949470482052	-0.45259923512249	-5.91893751190505
H	4.35500427330149	-3.74531466264181	1.11766631189157
H	4.00202225705153	-4.33561162056417	2.74824139230991
H	4.43901864128299	-5.47705133619558	1.46660995719086
H	1.25520181063536	-5.23879528014851	-0.46786857295628
H	2.67533685512783	-4.24644212636898	-0.80483840222406
H	2.86996479957397	-5.95835822442787	-0.39708189726457
H	4.12544681266906	0.30523097562942	4.27369732837154
H	3.87670441433551	1.94979359404225	4.87890529345906
H	4.15887819535007	0.61939867673587	6.01360614424710
H	0.85260234957239	-1.05092765807293	5.62487534380125
H	2.27108725980994	-1.48686825750098	4.67005278969444
H	2.44421807766420	-1.11595941527099	6.39310492378779
H	4.33758811185756	3.68522864591911	0.47864306340853
H	4.27900535774443	4.30742395630195	-1.17703919714817
H	4.49750467366243	5.42132552048725	0.18297768393173
H	1.03542913601513	5.21003626694951	1.53795209014643
H	2.34378988657527	4.16172833284573	2.08841071017239
H	2.65854647306675	5.87443149581373	1.76419282274927
H	-2.74726208254155	-4.33643046105139	-2.05429911623247
H	-2.70384670059752	2.18365551343217	-4.03779376125384
H	-3.05864952386177	4.37572183581327	2.54117547940601
H	-3.18642772695562	-2.31393410994369	4.60426018235470
N	-0.56744485600014	0.25169812575444	-6.73953154900436
C	-1.15433638281576	1.44726824868314	-7.36870177112661
C	-0.67084648458717	-0.95952477585697	-7.57139821553222
H	-2.21580832450595	1.26568515724359	-7.53568992180870
H	-0.65907275071767	1.64658650198990	-8.31969010703280
H	-1.72581506247899	-1.17067592969183	-7.74513570526767
H	-0.21215785298511	-1.79179319161936	-7.03819170454322
H	0.41300772611376	0.43192825559467	-6.52678026349669
H	-1.02823071769740	2.29522832910899	-6.69653215710594
H	-0.16359751205919	-0.80016870199174	-8.52331493003369

R[4]A:diethylamine

$CHCl_3$				DMSO			
C	-2.67346074724655	-2.18645726997810	2.46062910490394	C	-0.17029818147080	-3.33434805529257	0.13344919705838
C	-3.11777839975245	-3.16803252026159	1.57950092029292	C	-1.18644006025595	-4.12326589237622	-0.39759889725719
C	-2.27493852485472	-4.16658349704245	1.12490180851704	C	-1.64564928119668	-3.94047146126942	-1.69148778446452
C	-0.95347463305956	-4.20770915045062	1.55334422665447	C	-1.08835698208510	-2.95518961408251	-2.50508743045993
C	-0.47628302372064	-3.26063249744402	2.46700202076469	C	-0.03134905783031	-2.16992555159032	-2.01743037204144
C	-1.34931457986129	-2.25872420543774	2.86821356144011	C	0.37145927234778	-2.36769099226871	-0.70377873329151
O	-4.42562809691485	-3.09871260104135	1.16197876384666	O	-1.72838850996354	-5.09341704734346	0.41263294586721
O	-0.16313013239669	-5.19011358971866	1.05272024395909	O	-1.58548351074687	-2.81271735323339	-3.74933475514357
C	0.93604261215575	-3.33452801844322	3.01234775238997	C	0.63371458628902	-1.13253265526870	-2.89955979550967
C	1.84168288945990	-2.33989495708357	2.31992278533894	C	-0.07473119578374	0.19954500284212	-2.78019834701748
C	2.37260907588807	-2.64091327519235	1.06348000511973	C	-1.18184862011498	0.47038620415137	-3.61176266363365
C	3.20101007558944	-1.73275647627316	0.42048130682298	C	-1.83520058338400	1.69886620917174	-3.46679401072829
C	3.48055822117007	-0.49508798998296	0.98534023596451	C	-1.42005664768560	2.64122595945093	-2.53421908715717
C	2.94960408800872	-0.15331003263125	2.23435931255065	C	-0.31481781574647	2.39253244327376	-1.71386905870267
C	2.14057521000524	-1.09623300728052	2.85996964482152	C	0.31088789523904	1.15717477445466	-1.85688433727011
O	2.05982784742819	-3.82010993361910	0.45746542028865	O	-1.59467206421256	-0.40849437206184	-4.51963732734018
C	3.22611749180166	1.20222532120043	2.85958999014187	C	0.18151589793961	3.40462922019430	-0.69806310118999
C	2.31594071507587	2.23879089186258	2.22642413998741	C	-0.51929403000682	3.18409654712977	0.62957113522157
C	2.77930935593488	3.10151456999793	1.23569471406507	C	-1.65515611817176	3.91739347137384	0.96797100705141
C	1.94011411883407	4.01131579906971	0.61764160848118	C	-2.32246112980159	3.70533590406598	2.16139265869358
C	0.60665684333688	4.09811762633789	0.99873035329678	C	-1.85380156482724	2.75902444216223	3.06623413386217
C	0.11302760866967	3.28814513771127	2.02668508549401	C	-0.69922359198308	2.02113865125016	2.78097810623161
C	0.98009810959273	2.35590580389725	2.58337667703963	C	-0.08833469062113	2.23914199379192	1.55078751206607
O	4.10373290931307	3.00645458405950	0.88116428730868	O	-2.09250572442721	4.86246212590104	0.07362943091400
O	-0.15906040303307	5.01378312085947	0.34786447910084	O	-2.55450303285252	2.63258102424373	4.22345961927446
C	-1.29908733053487	3.44722717343186	2.56029507395438	C	-0.10341391723209	1.05259312558017	3.78884181090528
C	-2.20982660776736	2.37678105120052	1.99474236953319	C	-0.62737746343454	-0.35283442214768	3.56040548997453
C	-2.83418663799980	2.56673616899253	0.76357964388768	C	-1.76307396566392	-0.80221088484812	4.23222886699356
C	-3.65687355215355	1.60137283871884	0.21128135646027	C	-2.28172655355538	-2.06624603189224	4.01447781148263
C	-3.89257599129212	0.41146240827736	0.89106218890074	C	-1.65600617247198	-2.93266313955036	3.12466332708624
C	-3.30816618939241	0.19024977175151	2.14268978839229	C	-0.48388324835649	-2.54290269987839	2.46861341115532
C	-2.46265204701584	1.17504084977999	2.64006628649573	C	-0.02709149802520	-1.24673739979153	2.68423366381388
O	-2.61334536537303	3.75348169952317	0.11128236405286	O	-2.35371208303902	0.05278387022077	5.12907974178666
O	-4.72412375021051	-0.47073163285154	0.28272200916032	O	-2.22391029348833	-4.15724223695619	2.96895192304114
C	-3.59383210884192	-1.07648880336103	2.92655009240699	C	0.27646399290316	-3.50333873585900	1.57145743648977
C	0.94518022370259	-3.22828320711275	4.53926446205231	C	2.14160102987441	-1.04553517683421	-2.65737819202171
C	3.16176835631337	1.17152842955696	4.39148887163440	C	1.71348072166739	3.42284294776105	-0.60227933333147
C	-1.29593421978102	3.55832054425117	4.09231401047816	C	1.42769810130584	1.15923149112402	3.83880695751296
C	-3.56882021480372	-0.84410235586271	4.44247076906663	C	1.79313697360824	-3.39757957193306	1.78594536165075
C	2.30033801730358	-3.46538124178962	5.20424881358999	C	2.89704655268392	-0.17717716847347	-3.66231044570631
C	3.89628052087347	2.30469686634890	5.10888036035925	C	2.32850451154416	4.71849923416741	-0.07309503182393
C	-2.47381851413855	4.32289436108765	4.69741547589285	C	2.06824149111868	0.74307947556679	5.16323352610910
C	-4.34301308802755	-1.86466542933817	5.27773794263117	C	2.59109723266858	-4.65253215284563	1.43196125120727
O	4.29718502201650	0.31507671557483	0.26309401426520	O	-2.12797671640792	3.80911995018739	-2.49415467391386
C	2.17280733393688	-3.25341951955125	6.70807640566181	C	4.31901871937951	0.06936802064475	-3.17278689530027
C	2.85568579106322	-4.85166033260676	4.90117167391062	C	2.91064429916239	-0.79579307573179	-5.05458968516658
C	-4.43577065384842	-1.36674997280823	6.71626785762787	C	4.04113563183845	-4.46826418099968	1.86607352701634
C	-3.74688024432680	-3.26740155018515	5.24813390895275	C	2.52972310164548	-5.02865951362842	-0.04370112370086
C	-2.25067443528553	4.49026838016270	6.19644821214627	C	3.55916695361337	1.06158436465043	5.12375513876621
C	-3.83227999662479	3.68362394146017	4.43677601325848	C	1.85545147899843	-0.72244064622262	5.52308844657845
C	3.94678240517645	2.00156600201734	6.60286597281062	C	3.84707023132115	4.63681956123615	-0.18604226718554
C	3.28705252447847	3.68299582276556	4.87790828205179	C	1.93076907079968	5.05520510208521	1.35899145980106
H	-0.97924611120418	-1.49807914011487	3.54761202780881	H	1.16991359395332	-1.74600438638566	-0.31553977338789
H	-4.59710530835747	-3.77987668965137	0.50159821357826	H	-2.46081204490567	-5.52981901219300	-0.03877127415298
H	0.74178538322996	-4.81339522374050	0.94090428105544	H	-1.51341086853236	-1.83957706777339	-4.06937232167523
H	1.31340934925697	-4.33127665734622	2.77195928983379	H	0.49027023813938	-1.46788015459395	-3.93119946693932
H	1.72343876780140	-0.84843842854449	3.82867019332990	H	1.16438974087075	0.94699759321721	-1.22106727858003
H	1.77234891124308	-3.63515909806789	-0.53395573574668	H	-1.07406331705383	-0.12016842019155	-5.89326670848506
H	4.25868239923690	1.47271406553154	2.61277892699941	H	-0.10347807183755	4.39873815522488	-1.05781686210518
H	0.59687901859600	1.70174646949141	3.36018064559794	H	0.79701829293584	1.65899512716603	1.31361190739846
H	4.28892089097875	3.59010846700935	0.13615746027138	H	-2.90754393089214	5.27132031865185	0.38917229758803
H	-1.07864808014245	4.69170630694547	0.30723867590074	H	-2.43120093678278	1.74270408079848	4.60840302274067
H	-1.67296943573945	4.41138657039213	2.20042713979336	H	-0.44961656132270	1.37075727211504	4.77783546662015
H	-2.00337054977103	1.00403123095373	3.60580645944271	H	0.86955858065584	-0.93249636705408	2.16183559549937
H	-3.07433898318055	3.75869321671851	-0.73497517251337	H	-3.15848979938338	-0.34165594116081	5.48649476439378
H	-4.67150399163845	-1.36070917873776	0.67732676027991	H	-1.98796479668857	-4.54365515878938	2.10288346308719

H	-4.62063611995896	-1.37708030106028	2.69336532126630	H	0.00722684815932	-4.51719715785873	1.88489343702992
H	0.23097623040402	-3.96630592474974	4.92571469307477	H	2.55714900308102	-2.06110717569858	-2.68153881585400
H	0.56406139236146	-2.25168139136436	4.85671360317896	H	2.34417169447671	-0.66407735485494	-1.65145428126475
H	3.61165290503878	0.22730648296340	4.71744657280420	H	2.10444055558620	3.25794162085308	-1.61220888146116
H	2.12203276084391	1.14746535416531	4.73791482900894	H	2.08117392588267	2.58482466953444	0.00067747131656
H	-0.37959476320739	4.08697987902576	4.37520840107961	H	1.68857782019068	2.20806276114451	3.66087410448452
H	-1.22367917438883	2.57155423695656	4.56292016550850	H	1.89062356980734	0.59353100018072	3.02286125794626
H	-4.01022389861765	0.14087303776344	4.62935298367150	H	1.96008784474718	-3.18656035796444	2.84770838939049
H	-2.53875222511858	-0.79071019648401	4.81364250582492	H	2.20819998153881	-2.54257135402651	1.24116066403170
H	3.00936097754590	-2.72376930346300	4.81664733682856	H	2.38642586246550	0.79243747793640	-3.71991529164733
H	4.92797176129107	2.31984910398816	4.73125156766369	H	1.98226217294893	5.53515556646637	-0.72170999399924
H	-2.47868466995866	5.32276951281250	4.24144411665395	H	1.61138307340307	1.35571870541497	5.95294854650486
H	-5.36230559421598	-1.91777378760914	4.87078212949156	H	2.16653052658866	-5.48380331797784	2.01194219781056
H	4.29892900371725	1.23218174949561	0.59128793768895	H	-2.04868390729310	4.23711425983474	-1.62170491272591
H	1.80075301196215	-2.24977764285369	6.93617107260593	H	4.32013342187834	0.56598151222856	-2.19752173165148
H	1.47376239378552	-3.97625955876422	7.14326500509539	H	4.86250827086929	-0.87632682431730	-3.06661024221019
H	3.13693513674315	-3.37806869322068	7.20933660364551	H	4.87650189520810	0.69806983381818	-3.87335901169774
H	2.14490178144048	-5.62786769586406	5.20610677140479	H	3.41262391715531	-1.76960728788086	-5.03477310152690
H	3.06552357832156	-4.98758930368810	3.83706999218926	H	1.90167322363588	-0.94626189788345	-5.44712389313750
H	3.79019967925466	-5.02173486681834	5.44349560049878	H	3.44633447771571	-0.15594769909885	-5.76186235243747
H	-3.43740784167137	-1.29143235236563	7.16124619203565	H	4.50641587696921	-3.64673137248723	1.31014183379311
H	-4.90075626042421	-0.37787851104988	6.76792685181273	H	4.11206137958941	-4.23363048211769	2.93238944769157
H	-5.02437000201185	-2.05127827562683	7.33358126660966	H	4.62704615608340	-5.37246171736053	1.67741488599353
H	-3.75844224938567	-3.70292879004447	4.24766586060363	H	1.51235124105245	-5.25227089536419	-0.37077755459212
H	-2.70562943463376	-3.25120398165308	5.58838203285712	H	2.90516071549601	-4.20961986050529	-0.66691434884972
H	-4.30701585248312	-3.93235934025882	5.91230036930663	H	3.14930067686056	-5.90969445625210	-0.23682495440343
H	-2.24398602492145	3.51431341559851	6.69434959355633	H	4.05667323057662	0.47160800715960	4.34593876204863
H	-1.29564887764798	4.98059733231068	6.40691653722280	H	3.73600308883215	2.11927730285619	4.90709446091893
H	-3.04626639750873	5.08886188727915	6.64911662075425	H	4.03788221897538	0.82558435501499	6.07850033126034
H	-4.06225382477989	3.62210209369226	3.37118994672070	H	0.79872135767084	-0.96737789295070	5.64687594218073
H	-3.86673889119943	2.66637261072652	4.84090439671735	H	2.25680627361332	-1.37568369626462	4.74058835637523
H	-4.62326788806295	4.26460458459891	4.92052901854604	H	2.36995953605664	-0.96171188129237	6.45874073669791
H	2.93515444535573	1.96872934549059	7.02255208881030	H	4.23540919531421	3.83320381890138	0.44963309361799
H	4.42191275584381	1.03600836177678	6.79978565995439	H	4.16165555680271	4.43239210977404	-1.21391212835020
H	4.50567661792787	2.77124705940106	7.14271556225196	H	4.31701357840555	5.57124233598606	0.13395608616750
H	3.32476251996219	3.98324059879454	3.82957908089602	H	0.85664682109454	5.22079621613723	1.46179024410306
H	2.23626810060336	3.69748199838075	5.18784453696106	H	2.20654730562345	4.24141687745392	2.03876808841589
H	3.82043485998934	4.43690942917327	5.46466909329305	H	2.44588235118455	5.96149983321998	1.69174882250811
H	-2.62525090037075	-4.91040226795470	0.41589545096512	H	-2.45439320043676	-4.55278088610545	-2.07943103272516
H	3.63611667534902	-1.97898394987593	-0.54083961153470	H	-2.69163624794655	1.91981031809257	-4.09601617609888
H	2.30680420183512	4.65713958453729	-0.17414898199046	H	-3.21489995961168	4.27391905299891	2.40477596729634
H	-4.13062068158454	1.76207225069527	-0.75232212884437	H	-3.17955192166764	-2.39163765590667	4.53098862558762
N	1.14197136417858	-3.42211797990762	-1.94699644124189	N	-0.80164652106561	0.13313837720220	-6.92275214022155
C	1.97534641558938	-2.64675468806057	-2.87106878232657	C	-0.56797223040823	1.59379173279129	-7.02726310166820
C	-0.23142556192535	-2.91036909855734	-1.81926375940097	C	-1.88328262634525	-0.35653572483322	-7.80943462031412
H	2.17775289595224	-1.67247832492966	-2.41971874449910	H	-1.47323329478226	2.08108114481571	-6.65907243323150
H	1.42998597566691	-2.46088431126791	-3.80762911945977	H	-0.45543168470214	1.83581780342985	-8.08664981046019
C	-0.31691625139282	-1.60461731783648	-1.05356485351715	H	-2.79146494760181	0.17520846148676	-7.51776467950086
H	-0.81064377357857	-3.67515565817213	-1.29608913177620	C	-2.05246899132622	-1.85307172812053	-7.67761117505129
H	1.07991163206812	-4.36872036901273	-2.31339491736657	H	0.06224574824702	-0.36232740025880	-7.14782299459490
C	3.27306598130524	-3.37261808192885	-3.15890034691380	C	0.64959053238122	2.01560594445052	-6.23729746694899
H	-0.67511196792622	-2.78701357335964	-2.81743605401004	H	-1.63201742933675	-0.07033042294474	-8.83324496579243
H	-1.36203393629528	-1.29131333814301	-0.98308651794722	H	-2.84805356050180	-2.18948023930351	-8.34507506371244
H	0.23759645809648	-0.80263266548850	-1.54660558129440	H	-2.32147544066849	-2.13323415831508	-6.65668111605030
H	0.06788680151673	-1.71327730572011	-0.03743393006768	H	-1.13413839930563	-2.37847477931612	-7.95369656096613
H	3.90008993786795	-2.78149989337872	-3.82983493241478	H	0.78242283093754	3.09532926648445	-6.32951738608578
H	3.08327672321471	-4.33786381794034	-3.63820019175079	H	1.55191480263702	1.52889722851055	-6.61626790278507
H	3.83634794905673	-3.55750207454186	-2.24034517656129	H	0.53985824299544	1.77345095974829	-5.17767601140957

R[4]A:pyrrolidine

CHCl$_3$				DMSO			
C	-2.59166158114510	-2.27789826633359	2.44035116025354	C	-0.25449646795462	-3.38441131038309	0.19115569526081
C	-3.03940903270993	-3.29420250559886	1.60164213879495	C	-1.26992349305569	-4.20415133003145	-0.29277650462334
C	-2.19450263621509	-4.30144779220796	1.17018309769371	C	-1.76373634525608	-4.06304222985262	-1.57906845630849
C	-0.86728020819863	-4.31301182023415	1.58020854268006	C	-1.24266016139193	-3.09140054558032	-2.43191988335294
C	-0.39210376282418	-3.33997780765922	2.46593784667285	C	-0.19030907361976	-2.27348892677257	-1.99124236017941
C	-1.26628729199584	-2.33152931096428	2.84565710023496	C	0.24699883884424	-2.42910395400017	-0.68299724579610
O	-4.34962488545320	-3.24220896377434	1.18966753027357	O	-1.77460005823279	-5.15990301297118	0.55744590658310
O	-0.06088093842561	-5.28474238660672	1.08122919858966	O	-1.76885184768410	-2.98866307016998	-3.66839717875172
C	1.02065578037709	-3.39483278505752	3.00859694054150	C	0.43922244484831	-1.24734705091901	-2.91246087828152
C	1.91700246539228	-2.37850936145881	2.33729998247060	C	-0.26931722734352	0.08499496885491	-2.78755664451598
C	2.43562458153414	-2.64234922739524	1.06817498998839	C	-1.42478927353122	0.33249816062195	-3.56081205260026
C	3.30800051800251	-1.74593045730904	0.47002445281254	C	-2.06681635622990	1.56692038961636	-3.41034608580477
C	3.61397567892658	-0.53509356624021	1.07665159400952	C	-1.60237562447961	2.53212212825617	-2.52520953257722
C	3.06295289907448	-0.21072876700530	2.32154328977533	C	-0.45753743646045	2.30186342319897	-1.75661284950553
C	2.23464800495358	-1.15724544647824	2.91651677395785	C	0.16143936476528	1.06381087467423	-1.90763035629974
O	2.06704930716528	-3.76902112166523	0.39553466789112	O	-1.90003525496981	-0.57678406281746	-4.40468373021393
C	3.32433994705275	1.14018105701510	2.96498770264386	C	0.08568208394805	3.33394085810474	-0.78549536413459
C	2.45248526875992	2.17680763271817	2.27841941212281	C	-0.56343899936498	3.15388179872303	0.57446898558184
C	2.96981986881848	3.03073677334264	1.30718533833842	C	-1.66919183940953	3.91712584782642	0.94495964992172
C	2.16221801231707	3.91465591606042	0.61391364875288	C	-2.28880898068842	3.74409658380789	2.17018038108442
C	0.80591197949223	3.98885000385571	0.90546258641891	C	-1.80034872503090	2.80731118912218	3.07441111965387
C	0.25902965849204	3.20012190172273	1.92251699488521	C	-0.67304871361177	2.04113208708505	2.75684629917187
C	1.09638180160653	2.28705623347082	2.55126680689195	C	-0.11130379263307	2.22008706591795	1.49720307002337
O	4.31577575176929	2.94936441805206	1.04310015465691	O	-2.12557205497370	4.85209455220591	0.04935461523780
O	0.06777432335131	4.87133049054452	0.18073558423525	O	-2.45213770733096	2.71695505143412	4.26363975724717
C	-1.18215338750094	3.36547372723221	2.37055299582563	C	-0.05366567401738	1.08240856166700	3.75991560985204
C	-2.05382495002030	2.26069542526826	1.80917496606919	C	-0.59453004354002	-0.32180816906023	3.56753788769568
C	-2.59942821754938	2.37938275449646	0.53276733487114	C	-1.71984533195861	-0.74878242167049	4.27087538749967
C	-3.39696835836170	1.38747020518411	-0.00891806825119	C	-2.25138413633306	-2.01328048689237	4.08855342774053
C	-3.68268431674580	0.24187364268497	0.72565791216709	C	-1.64991482343043	-2.90222040701493	3.20407970554385
C	-3.17112295634246	0.08945386509359	2.01856009748593	C	-0.49001971724561	-2.53324401343444	2.51474099948483
C	-2.35038702040144	1.09907734229882	2.50729650027797	C	-0.01984466826476	-1.23681195301512	2.69595201116371
O	-2.32991443418083	3.52384150002377	-0.17415805683378	O	-2.28538129995885	0.12825487469067	5.16249485874370
O	-4.48539515525010	-0.66798769326151	0.12051952874938	O	-2.22816978993072	-4.12609316624844	3.08703328892384
C	-3.50020841934314	-1.13651410974890	2.85089632426672	C	0.24101131362058	-3.51538630110870	1.61705924480629
C	1.02027849058854	-3.31577789935606	4.53757542808913	C	1.95434755656176	-1.15209875059029	-2.71351595961041
C	3.18298476893517	1.11180900288208	4.49088364369435	C	1.62008921676325	3.34225186929344	-0.74647860704972
C	-1.26168052234381	3.54889250168779	3.89400164002541	C	1.47891280390699	1.17843735511107	3.76118498377079
C	-3.49318201041156	-0.84426192213025	4.35629924156373	C	1.76421192373028	-3.40539129221633	1.77407929483914
C	2.37198670940678	-3.55161610864948	5.21028160520291	C	2.67753191637556	-0.31153211110345	-3.76563218282234
C	3.86861934606897	2.25325851305118	5.24313064678081	C	2.26342899502245	4.64352020517115	-0.26680928854143
C	-2.47309991906245	4.33459253858591	4.39801695046727	C	2.15740304589829	0.77423991794720	5.07048262723574
C	-4.27192530276399	-1.83084367916347	5.22745863982573	C	2.55209973872354	-4.66249181321242	1.40388609253489
O	4.46494896719162	0.27063362671481	0.39031892626091	O	-2.30934034027927	3.70027540391819	-2.47802565237964
C	2.22780184022961	-3.37586897904990	6.71755764154107	C	4.06424342075719	0.07972037161624	-3.27113475041565
C	2.94765359399662	-4.92350413305379	4.88014009451936	C	2.77344073650351	-1.04173716960178	-5.09940681082922
C	-4.38126837947714	-1.26937247406454	6.64177104543407	C	4.01384380841248	-4.47507046087607	1.79550019860219
C	-3.66859266761442	-3.23016002444468	5.26640221340876	C	2.45074028094154	-5.04707349923639	-0.06756231654999
C	-2.34607387634898	4.55012150828124	5.90229420547606	C	3.64973989722524	1.07409178041038	4.97823315234551
C	-3.81519613633455	3.69277484376279	4.06907186985498	C	1.93931569719848	-0.68275010495744	5.46058811400238
C	3.85409097233593	1.94723467385213	6.73770360662762	C	3.77604817453276	4.54790040768655	-0.43593167817522
C	3.25143628833629	3.62342750683288	4.98643712454163	C	1.92422728702811	5.01171250480704	1.17273202182539
H	-0.89349209392103	-1.54547325850094	3.49639389477843	H	1.04254080218839	-1.78243445693647	-0.33078177232991
H	-4.52350561623564	-3.94550798131389	0.55299019116632	H	-2.50798176717750	-5.62453202504740	0.13665978730207
H	0.82101725118757	-4.87410711844178	0.92024461004429	H	-1.74390610446955	-2.01487170911883	-3.98786243512450
H	1.41557845146867	-4.38268746781005	2.75366042899634	H	0.26460715756146	-1.59555183792940	-3.93588198256991
H	1.81459941045864	-0.92970903647423	3.88998059787237	H	1.04768242270497	0.86929978502350	-1.31345856033251
H	1.75815174404479	-3.47587839328318	-0.56300456640543	H	-1.91843396599627	-0.19262771224682	-5.83153172471124
H	4.37138619459904	1.40351904798013	2.77098306727173	H	-0.20525391712893	4.32151093184547	-1.15833513414973
H	0.66945755786204	1.64198760216855	3.31446181827436	H	0.75206011986339	1.61731115400406	1.23602309132268
H	4.54275835569326	3.52674861423966	0.30449685179689	H	-2.91777997932033	5.28441192612104	0.39077788820592
H	-0.83914466951629	4.52522303737458	0.08829719174955	H	-2.34009449028521	1.82639104288154	4.65005216849039
H	-1.54568811076595	4.30899871050145	1.94653291870783	H	-0.36661366606366	1.41656118466168	4.75458484388380
H	-1.94906407150525	0.98339896552476	3.50723441339490	H	0.86804389140202	-0.93920051524163	2.14957504556421
H	-2.73726609638686	3.48133491559191	-1.04686368297518	H	-3.08251172980303	-0.25375042833756	5.54939410640240

H	-4.50089776107174	-1.52310176569083	0.58829882716405
H	-4.53060989465622	-1.42567684857776	2.61174387363967
H	0.31062881645428	-4.06906953278189	4.90523480592566
H	0.62372919369382	-2.34885012186179	4.86922421696258
H	3.62610043632160	0.17185725944250	4.84108444599684
H	2.12711212940525	1.07739768261591	4.78768497509780
H	-0.36310167075920	4.09622658905149	4.20113392311318
H	-1.21007821669696	2.58516456941296	4.41410242255703
H	-3.93865607216956	0.14749421303537	4.49864085882272
H	-2.46635192346529	-0.77241746867601	4.73656205300040
H	3.07711108692724	-2.79226854106772	4.84761565609076
H	4.91757234305866	2.28318233835114	4.91367743676398
H	-2.44358418546620	5.32007698719068	3.90990369480199
H	-5.28808050373022	-1.90725848487266	4.81340452847080
H	4.49491143010207	1.17680837542864	0.74620161453049
H	1.84061104346576	-2.38199629135447	6.96537396210979
H	1.53401050370105	-4.11738658100669	7.13072801335127
H	3.18926116187813	-3.49953317592092	7.22541521257619
H	2.24330379881616	-5.71648054969723	5.15790871276588
H	3.17148986462172	-5.03134203682154	3.81507154456389
H	3.87868798842321	-5.09591865155487	5.42885559469725
H	-3.38740835479222	-1.16663287831540	7.09288351263910
H	-4.85322762519119	-0.28180153191194	6.64562538536436
H	-4.97127721325377	-1.92965829290153	7.28463170861391
H	-3.67643785247228	-3.71435686578025	4.28795218565631
H	-2.62717439520085	-3.19260533955474	5.60641873278455
H	-4.22607050006943	-3.86587188466207	5.96175418749583
H	-2.37914971718771	3.59061257337273	6.43145106434748
H	-1.40187892463719	5.04105311797893	6.15858966124193
H	-3.16411137678798	5.16854363629895	6.28410148676499
H	-3.97604901931200	3.60131343511465	2.99237307138305
H	-3.88235722989501	2.68683559979567	4.49803902706359
H	-4.63338717075051	4.29066562043749	4.48339288309578
H	2.82420370358546	1.89657547626428	7.11000941728266
H	4.33501668781904	0.98834647487155	6.95544694503768
H	4.37493326287932	2.72434306024336	7.30539154426578
H	3.33893285697027	3.93050586519144	3.94244329495631
H	2.18511804052781	3.62072300280295	5.24074220386885
H	3.74159097313937	4.38311972335399	5.60379072860691
H	-2.54510704023692	-5.06447400596535	0.48078376413271
H	3.74319168870292	-1.97645650865473	-0.49650490353527
H	2.57346203902843	4.54633574686906	-0.16864230882778
H	-3.81304857778137	1.49336236957566	-1.00704878557626
N	1.10322548546460	-3.03659359766753	-1.91449280202491
C	1.36424081255665	-1.65909584687309	-2.36486146977493
C	-0.35733652119281	-3.07840125290443	-1.73509950966319
C	0.46335449521557	-0.80585160928238	-1.46947976527445
H	1.07366135983534	-1.54661356890354	-3.41860790674649
C	-0.66048111493010	-1.76383395729678	-1.01824104553299
H	-0.64790748793604	-3.96150279094318	-1.16303852593317
H	1.40286923794839	-3.70823381324873	-2.61530542881587
H	-0.86136613979180	-3.11220127139945	-2.71093084257603
H	2.42459750928574	-1.41518693731201	-2.27498921179228
H	0.08172755447182	0.06142406676046	-2.01292840921025
H	1.02229214274527	-0.43625471895353	-0.60630339799901
H	-0.63104168651158	-1.90560882499171	0.06444438371642
H	-1.65684823523620	-1.39396086941389	-1.27036657623898
H	-2.00256956031058	-4.54073170592323	2.23159156809816
H	-0.01627060174941	-4.52104200958042	1.96479686030011
H	2.37203117507393	-2.16684586178727	-2.71787166886573
H	2.17987369659397	-0.74307402998716	-1.72379863089941
H	1.97320840375123	3.15406327395924	-1.76614866747239
H	2.00315380785190	2.51432336012197	-0.13931641706599
H	1.74136776837327	2.22306218762762	3.56212459102779
H	1.91224634290120	0.59977532765159	2.93822130729695
H	1.96886969747168	-3.18421720644888	2.82726074859877
H	2.15839798928988	-2.55502003994854	1.20676372727094
H	2.09925656818642	0.60886615411944	-3.91503141908608
H	1.89822194495313	5.45004395512629	-0.91771819373289
H	1.73312739671671	1.40400045554969	5.86490322246182
H	2.14449736135786	-5.49084075184673	1.99993395497580
H	-2.17607338103071	4.15836259165193	-1.62792099951620
H	4.00333726342405	0.66356478910970	-2.34734141562786
H	4.66679077290097	-0.81224830817469	-3.06584933171355
H	4.59734667887929	0.67829547068272	-4.01582494601280
H	3.39930098057641	-1.93536226190567	-4.99882488271349
H	1.79305059793640	-1.36124532417575	-5.46108055371035
H	3.21913134307360	-0.40340722248616	-5.86797503661899
H	4.46158502250092	-3.65496815524629	1.22325981253867
H	4.11533059979464	-4.23653194250288	2.85843566373541
H	4.59528981885161	-5.37917534964204	1.59307089290932
H	1.42550173485071	-5.27473194339669	-0.36553302680113
H	2.80736526033048	-4.23081841156468	-0.70537400027365
H	3.06700194125066	-5.92776934555085	-0.27253381197445
H	4.11521441390689	0.46523514744793	4.19515123509306
H	3.83158197676307	2.12577761882017	4.73776808661431
H	4.15631100462254	0.84867495274014	5.92111322725827
H	0.88433064012133	-0.91267284268856	5.62254633520313
H	2.30740034745211	-1.35375358012126	4.67678773790518
H	2.48086462218566	-0.91320117352951	6.38312841711968
H	4.18251640670710	3.75375810752002	0.20032494867935
H	4.05019799397463	4.32119811623938	-1.47070171684077
H	4.26432484055749	5.48461167951707	-0.15201220855235
H	0.85623456378850	5.18770489083163	1.31416071448897
H	2.22021194523403	4.20951823973917	1.85781195229158
H	2.45830226875517	5.92041165304427	1.46698180002327
H	-2.57129982096891	-4.69837842628473	-1.93080057855475
H	-2.96568298238560	1.77149397467672	-3.98324229327227
H	-3.15894600187431	4.33550992925007	2.43856859497352
H	-3.14010953123511	-2.32262857468548	4.63009593881243
N	-1.83677403859680	0.07880984649609	-6.89320996779231
C	-0.41303478467386	-0.03343133010642	-7.29158854514022
C	-2.19593058360914	1.50990676785037	-7.08940191996848
C	0.19367211693937	1.20399035730551	-6.65462265673676
H	-0.35613325186306	-0.00346821255244	-8.38209414151419
C	-0.89939786158530	2.27945383101224	-6.79672464101537
H	-2.44062323121307	-0.54144920290352	-7.42867490481492
H	-2.51965986053574	1.63749891703783	-8.12293909140184
H	-0.00748576183465	-0.97803802550717	-6.93045334377588
H	-3.02231318690686	1.76039598511359	-6.42547384906349
H	1.13162439993608	1.48514180680701	-7.13388406096341
H	0.39846398277887	1.00793734539999	-5.59939649303676
H	-0.98918565975521	2.86803780190818	-5.88300765865296
H	-0.68048765135776	2.96332874647821	-7.61793360350534

R[4]A:piperidine

CHCl3				DMSO			
C	-2.62448618974627	-2.30765905695827	2.49832872274665	C	-0.15954688763853	-3.28040519670638	0.07180722396059
C	-3.07806509102139	-3.32614293680433	1.66572651324060	C	-1.15619032103645	-4.09697858744736	-0.45498031828789
C	-2.23061498144560	-4.32152395362887	1.21267638149195	C	-1.64761003874121	-3.90739301016159	-1.73600164657399
C	-0.89522501153268	-4.32100816212824	1.59516073306069	C	-1.14545061697816	-2.88481805829406	-2.53886014006924
C	-0.41369230011915	-3.34896571247801	2.47919687904492	C	-0.11327668314778	-2.06605142080435	-2.05485952394851
C	-1.29129378685922	-2.35105969102327	2.87857645241908	C	0.32480511405628	-2.27482264797509	-0.75427857858965
O	-4.39716601219364	-3.28650815545359	1.27942870991391	O	-1.64601450743978	-5.10101867491916	0.34702386338074
O	-0.08900434708702	-5.28183860210056	1.07498640858873	O	-1.66960927719448	-2.73546274246815	-3.77227952960057
C	1.00426504740297	-3.40037666708446	3.00672658681069	C	0.49413297571714	-0.98381361522298	-2.92352611159838
C	1.89944664124697	-2.38035351277249	2.33872275159689	C	-0.24015648734710	0.32700924522786	-2.73585168364368
C	2.41227345319127	-2.63839427908446	1.06561996042563	C	-1.39447341091108	0.58807452871326	-3.50568290191409
C	3.30948629669376	-1.75763667236109	0.48248834225507	C	-2.06206623843930	1.80245878174278	-3.30521728855878
C	3.63123095206686	-0.55453772415514	1.09651411116841	C	-1.62301392927637	2.73449832224746	-2.37267655933042
C	3.06832110241781	-0.22336951345283	2.33415326210548	C	-0.48145815975475	2.48927335598029	-1.60326290060761
C	2.22965832210983	-1.16501626537629	2.92374812426660	C	0.16377520727865	1.27193116649472	-1.80745659328491
O	2.00637437938550	-3.73686908465073	0.36712428904245	O	-1.84140710851051	-0.28803293279444	-4.39591304242914
C	3.31246175711577	1.13658001038446	2.96443444020294	C	0.02900583638096	3.48214448942835	-0.57455686462264
C	2.42576023075536	2.14853411134447	2.25891382254388	C	-0.63533660764400	3.21936332190134	0.76433611723174
C	2.92920287769809	2.98386208800173	1.26413212543460	C	-1.77039057409547	3.92894380831034	1.15193610479535
C	2.10702226519594	3.83321836691593	0.54515050437551	C	-2.40766091587554	3.67385602865677	2.35356611570425
C	0.75020324037653	3.89872722647106	0.83712554791901	C	-1.90719902868872	2.70629211459356	3.21804258404310
C	0.21768311880254	3.13782508926907	1.88326491700947	C	-0.74793305329422	1.99552861842295	2.88625390839765
C	1.06885231702043	2.25179130583132	2.53268868682292	C	-0.16988015929021	2.25566233520232	1.64849929963298
O	4.27756074583595	2.92208444787864	1.00507245739419	O	-2.24082301655692	4.89458097122600	0.29732483192083
O	-0.00007643224212	4.74786339165621	0.08665026628599	O	-2.58191022555525	2.53033932161737	4.38419848912377
C	-1.21809819364644	3.31581159478642	2.34261537898671	C	-0.11183354560890	1.01087822778176	3.85297862632971
C	-2.10817169829394	2.21103029992659	1.81239625627171	C	-0.60832933210884	-0.39829749087930	3.59169664587194
C	-2.69950769011336	2.32800045984658	0.55649813623679	C	-1.71961944288153	-0.89376191471935	4.27141745449211
C	-3.52614987136611	1.34109825982904	0.05043731887951	C	-2.21250370426781	-2.16324897677500	4.02731374604660
C	-3.79105150904410	0.19942685523753	0.79824756197244	C	-1.58391251128887	-2.98844535189032	3.10107094243425
C	-3.23214942936891	0.04823770550300	2.07186640577394	C	-0.43553915474657	-2.55112598072136	2.43295697666111
C	-2.38690242955588	1.05370693019332	2.52494707610303	C	-0.00533887732302	-1.25106759350842	2.67761151170412
O	-2.44368229058056	3.46592446724610	-0.16681071206098	O	-2.31223152766583	-0.07880213511883	5.20320321566602
O	-4.61903404206736	-0.71005135913134	0.22665422307942	O	-2.12519650325894	-4.22207272273854	2.92347337363517
C	-3.53460066538946	-1.17235633090635	2.92092374937843	C	0.32989829889846	-3.46599644194293	1.49362368651750
C	1.01512238234876	-3.33118934189761	4.53621768156970	C	2.00716380216838	-0.86361773771683	-2.72829282543164
C	3.16518499234446	1.12375293196952	4.48942053555725	C	1.56241521646481	3.51799427220660	-0.51397624400800
C	-1.28164959057442	3.52410708329507	3.86385581517502	C	1.41727505202618	1.15099971538646	3.87456461147517
C	-3.49680844495070	-0.86537358122408	4.42283805056731	C	1.84778764940969	-3.31553230244919	1.66971753921091
C	2.37077840380695	-3.57382120169432	5.19850314624758	C	2.69693507638900	0.00897528648066	-3.77753457366873
C	3.83819310016489	2.27985564613561	5.23023278553492	C	2.17441508358549	4.80341809540443	0.04368035075937
C	-2.48588213601337	4.32137203865313	4.36676315341828	C	2.09726512761675	0.70728463626806	5.17014109640616
C	-4.25868457717386	-1.84415641974065	5.31679245194429	C	2.67690236088260	-4.53361826019468	1.26255681493875
O	4.49602156876915	0.24201545927280	0.41697995361564	O	-2.35276481664452	3.88536788243503	-2.27875036847649
C	2.23667284632001	-3.40174944207672	6.70689971130193	C	4.06562941601638	0.45847715898466	-3.28316604542545
C	2.93793346459822	-4.94735453327217	4.86159922968722	C	2.82213369699518	-0.71791746438089	-5.11068621013142
C	-4.34024529804897	-1.27170202454088	6.72821949455407	C	4.13013667581132	-4.31179748581146	1.66762384924317
C	-3.65380398655212	-3.24277528619220	5.35427634146821	C	2.59301956332205	-4.87105891393959	-0.22141623912261
C	-2.33407198432695	4.57529002286044	5.86264825607206	C	3.57907008705558	1.06254057459223	5.10754523988971
C	-3.83183227197501	3.66770747780827	4.07921025661904	C	1.92668283478165	-0.77375662774126	5.48627080586134
C	3.81287879869793	1.99616434652564	6.72879375497774	C	3.68908641522293	4.75423853735541	-0.12776852227849
C	3.21429459123243	3.64190223169717	4.94844428657512	C	1.82745987393681	5.07609732855014	1.50273647881727
H	-0.91443419513724	-1.56510855700966	3.52513107877469	H	1.10572887903931	-1.62887213170097	-0.36947188923331
H	-4.57680612042322	-3.99374342558159	0.64920086240391	H	-2.37176063831932	-5.55526669188776	-0.09757571471328
H	0.78595138213976	-4.86105181268255	0.90274085755840	H	-1.66180240741095	-1.75020433040285	-4.04711420950175
H	1.39881768714580	-4.38433686957068	2.74162810131694	H	0.32419168172215	-1.28632523964335	-3.96209516911698
H	1.80903087805089	-0.93591441178481	3.89551551108933	H	1.04834933152883	1.06665974937064	-1.21429328217634
H	1.63767658835511	-3.39960383945453	-0.55563402877481	H	-0.27642115203768	4.48140186337864	-0.90266375737229
H	4.35442739448976	1.41293187017246	2.76950057739596	H	0.71707056182422	1.69444147520217	1.37432931095068
H	0.65343675013815	1.62661210932110	3.31672924234057	H	-3.05479760935032	5.27900121602345	0.64451282556850
H	4.49428474723795	3.48735779597976	0.25450411429334	H	-2.43096958503121	1.63272947919145	4.73942061806590
H	-0.91138560892232	4.40549844915885	0.02689192710790	H	-0.44473461117208	1.29089227657073	4.85789327142047
H	-1.57898647248130	4.25367284286372	1.90756228424207	H	0.87278476529864	-0.90005921159175	2.14733600510470
H	-1.95018721325834	0.93931851470002	3.50901473878339	H	-3.09946524892612	-0.50346918942850	5.56483274278103

H	-2.87966082424141	3.42022794610530	-1.02509123127525	H	-1.89477812355514	-4.58309025452880	2.04493822001078
H	-4.60116664373345	-1.56932588425598	0.68640156615996	H	0.10174088179143	-4.49483475130885	1.79038480351337
H	-4.56591954728889	-1.47163357924871	2.70647385066081	H	2.44628646754906	-1.86884748470409	-2.75341225222493
H	0.30722961772533	-4.08452739206524	4.90422168785329	H	2.23179664636149	-0.46678033206760	-1.73329978248869
H	0.62342007302185	-2.36585464196635	4.87521326319713	H	1.93133976450340	3.39090544158697	-1.53755948148005
H	3.61482956515832	0.19260778477590	4.85166586594501	H	1.95377365890730	2.66574731579182	0.05290368169130
H	2.10944745693233	1.08394935971872	4.78236949728076	H	1.65083192979565	2.21066394154187	3.72502287406054
H	-0.37941654585176	4.07325597171385	4.15285759387271	H	1.87407271316029	0.62288377720294	3.03050896843218
H	-1.22909180113155	2.56910529124069	4.39783763657565	H	2.03688815898552	-3.12618299506332	2.73186100694594
H	-3.93867188105226	0.12709661432216	4.56444007175990	H	2.22017024304395	-2.43379621824087	1.13677144279008
H	-2.46340405369146	-0.79145772648683	4.78130129339353	H	2.07897128872688	0.90357145036630	-3.92768217655203
H	3.07558677420749	-2.81725911482949	4.83312610772079	H	1.79033558876251	5.63871943570364	-0.55822892673131
H	4.88837804443121	2.31127765336995	4.90889046899565	H	1.64496339726348	1.28308823554914	5.98963894109186
H	-2.46671417975457	5.29285548817146	3.85342458900068	H	2.29350435626642	-5.39370469839280	1.82892840486383
H	-5.28146765528967	-1.92366347042219	4.92324948185555	H	-2.24409114219750	4.29983577468295	-1.40276060876957
H	4.50733840968690	1.15623066049604	0.75290638268153	H	3.97922330060021	1.03789101825498	-2.35858623881649
H	1.85531596142248	-2.40744956136691	6.95933576211129	H	4.70612339507690	-0.40689997443549	-3.07896935708969
H	1.54246062081466	-4.14134330864895	7.12114831885213	H	4.57263563838636	1.08039662359631	-4.02695170255723
H	3.20024737057910	-3.53109426076929	7.20804133960607	H	3.47314467259419	-1.59286179864719	-5.00557657447626
H	2.23334319498052	-5.73703848292390	5.14559559280949	H	1.85325311719298	-1.06436087610521	-5.47996588077399
H	3.15045887350508	-5.05436815144230	3.79473683808659	H	3.25440494250135	-0.06713692716832	-5.87637138493794
H	3.87312724173422	-5.12336353042832	5.40087919439703	H	4.55432586989600	-3.46216829265692	1.12112112555264
H	-3.33783079698588	-1.16438281726430	7.15726437091517	H	4.21887436112786	-4.09992979278748	2.73735983481711
H	-4.81307741647058	-0.28522495174530	6.73318063784045	H	4.74089417561429	-5.19104987973112	1.44326612902644
H	-4.91597858551640	-1.92736917456000	7.38756120395624	H	1.57629515981270	-5.11989489176692	-0.53177529507965
H	-3.67358558604192	-3.73016321849368	4.37817458396559	H	2.92585238128456	-4.02216142177412	-0.82867780026657
H	-2.60870610938293	-3.20208111857374	5.68049269970204	H	3.23712060488086	-5.72455491491732	-0.45378128399629
H	-4.20153490018060	-3.87530722508457	6.05921460303544	H	4.07224655132666	0.50930173604922	4.30053710567577
H	-2.35423538192033	3.62950695818487	6.41529056194532	H	3.72614569252456	2.13042756640152	4.92058448243614
H	-1.38836739940271	5.07610228053248	6.08990435241456	H	4.08522704854456	0.80866225116459	6.04338860632069
H	-3.14821148705739	5.19940857702402	6.24185416532605	H	0.87884491287280	-1.04800882667537	5.62345142156750
H	-4.01024757449403	3.54341077986823	3.00930090759595	H	2.32669062769060	-1.39150057408714	4.67477230515956
H	-3.88798437287728	2.67584865883708	4.54000502151833	H	2.46646708081181	-1.03034872595558	6.40290942668822
H	-4.64358838470158	4.27596024266328	4.48943621422334	H	4.11440120064886	3.93516148411762	0.46292713781120
H	2.78050018763040	1.94753927466671	7.09252316650266	H	3.96905091154580	4.59391390915280	-1.17330747176261
H	4.29518331044955	1.04284374153654	6.96422367188524	H	4.15420409305367	5.68478452266455	0.21014412302222
H	4.32617377644410	2.78338447891921	7.28823772856461	H	0.75566959463790	5.21642794486326	1.65504461363257
H	3.30291858500836	3.93101990171622	3.89993746364334	H	2.14524877132058	4.24146036259054	2.13738066442525
H	2.14787093558022	3.63687717557916	5.19976596102146	H	2.33963825874307	5.97815730882099	1.85123239671605
H	3.69835485672256	4.41402055769592	5.55395618737169	H	-2.43967209011740	-4.54317006341218	-2.12065351792186
H	-2.58565491720634	-5.08487500242501	0.52718989226189	H	-2.96144967788947	2.01558900315154	-3.87509430270586
H	3.73311848377980	-1.98297160745220	-0.48990234879836	H	-3.30150175015186	4.22329469672091	2.63309628167096
H	2.50758355859312	4.44732825126363	-0.25560838808846	H	-3.09147820953314	-2.52514844730638	4.55185428246858
H	-3.97457702618240	1.44393891950113	-0.93295037183054	H	-2.09203390231151	0.31414253426774	-5.71852358912867
N	0.96150390613514	-2.98086541300381	-1.90660079529493	N	-2.22456912978159	0.75406303110901	-6.71964764382469
C	1.43675653521517	-1.75612528480644	-2.56940795106408	C	-1.97446680520592	-0.28785488804678	-7.74755853706529
C	-0.50041458112696	-2.96725380832634	-1.74507976622053	C	-1.33274033429766	1.93271881473460	-6.86944954022849
C	1.04006985823445	-0.51707493736619	-1.78517642856619	C	-0.52951192840319	-0.74248070728778	-7.68797495058134
H	1.01386325586987	-1.69571980854400	-3.58371914317114	H	-2.21041253181444	0.14554978759071	-8.72382924569528
C	-0.93307693436012	-1.77279922534855	-0.91482167184920	C	0.11767700290978	1.50090306218124	-6.79400931072548
H	-0.79726175629993	-3.90145891653715	-1.26272178062625	H	-3.19466696255182	1.05842087358501	-6.77998812150658
H	1.20740888377790	-3.77076718333350	-2.49616293526766	H	-1.55376778312436	2.39468449226689	-7.83616374223231
H	-0.98784220068000	-2.92608571728943	-2.73110670026922	H	-2.66788261104647	-1.10787358188463	-7.55321188238590
H	2.52371462920309	-1.82118060923238	-2.66776922209191	H	-1.58962211108517	2.63670732204240	-6.07689159885190
C	-0.46334671080158	-0.47157236284113	-1.54988660519038	H	-0.34774423264335	-1.24614816085489	-6.73150071596450
H	1.37904343998973	0.36980787169939	-2.32943873926986	H	0.74726499314531	2.38320459634366	-6.93633407770536
H	1.55824314303062	-0.52161150071528	-0.82130608632484	C	0.42077666837683	0.43790712216268	-7.84193164797280
H	-0.51969225634259	-1.86672828708604	0.09524078851258	H	-0.36857131850796	-1.48156540792716	-8.47718223674882
H	-2.02139091089469	-1.78536988040324	-0.81081663371820	H	0.33147159694285	1.11237128992398	-5.79171096841654
H	-0.72333444849610	0.37799404568246	-0.91112042333281	H	1.45710810912660	0.10332790172885	-7.75315960332530
H	-0.98309613205096	-0.32306433446989	-2.50544992497278	H	0.30591214793888	0.87032716135194	-8.84339437372665

R[4]A:morpholine

CHCl3				DMSO			
C	-2.08650670860101	-2.67036083025067	-0.46050715023743	C	-0.14568064886627	-3.28520851099019	0.04333245631971
C	-2.42733258258929	-3.69477204376864	-1.33864617023124	C	-1.11966698230231	-4.12264399376326	-0.49328171421141
C	-1.49299496103417	-4.62080241337261	-1.76668259248368	C	-1.60097327188771	-3.94395972375400	-1.77965855254750
C	-0.17780444973488	-4.52882432275869	-1.32962823254564	C	-1.11045125940287	-2.91195752843518	-2.57674398433729
C	0.19990351365795	-3.53172892298816	-0.42393483451034	C	-0.09966443693056	-2.07273519221769	-2.08415339226869
C	-0.76735262611027	-2.61507787358333	-0.03517018869261	C	0.32782608760768	-2.27093184704101	-0.77817160302942
O	-3.72516676061581	-3.73495410906723	-1.78723455256487	O	-1.59906761140926	-5.13375594115602	0.30573292076656
O	0.71695848458419	-5.42424011658044	-1.82681267430546	O	-1.62735442380956	-2.76929016643846	-3.81559024788223
C	1.60770979966890	-3.46329884046433	0.13016325482392	C	0.49603321092766	-0.97884068541399	-2.94702399616876
C	2.39798942711226	-2.34044218369217	-0.50564045604826	C	-0.24433549711130	0.32560500396386	-2.73937328013936
C	2.90640501104311	-2.49898158793771	-1.79640933367567	C	-1.41779169245406	0.58106321628712	-3.47892638872902
C	3.67224626278127	-1.49885889658854	-2.37791110557911	C	-2.08706251389603	1.79113288774395	-3.26641199783438
C	3.88290232396074	-0.29228837822918	-1.72433738396580	C	-1.63036179511536	2.72805781642029	-2.34736756388739
C	3.34020090947451	-0.07573783948484	-0.45262275424501	C	-0.47156362416268	2.48748660993846	-1.60227904225759
C	2.62016930463762	-1.12008649717651	0.11936704779316	C	0.17505248842773	1.27365541644745	-1.82043903797205
O	2.63021531870734	-3.63150636042584	-2.50602514016135	O	-1.88248995210955	-0.29808726890445	-4.36151005614817
C	3.49439661305363	1.26751074212169	0.23739698952585	C	0.05345483316799	3.48251898379956	-0.58280567938318
C	2.52073331542030	2.24926258473829	-0.39027650892445	C	-0.61173787122237	3.23496535503841	0.75878739881622
C	2.94901662139397	3.20618835905803	-1.30742342125497	C	-1.73013431009833	3.96808368308464	1.15101086348475
C	2.05733739088158	4.06132646288677	-1.93021093252061	C	-2.37159856734184	3.72228338365339	2.35227176111506
C	0.70326341558466	3.99343835754955	-1.62724607946409	C	-1.89033373804851	2.74161404348404	3.21256931142848
C	0.23691241773814	3.08465477262247	-0.67135820059325	C	-0.74516652201120	2.00973107653397	2.87804403976440
C	1.16203279807783	2.21367329814625	-0.10820617706041	C	-0.16385051864774	2.26033493428657	1.63994825565060
O	4.29334395751085	3.25689922036489	-1.58785921954848	O	-2.18032559969348	4.94639065934378	0.29975711094113
O	-0.11710458806616	4.85991040122769	-2.28001569358551	O	-2.56792282651505	2.57257775316168	4.37836543384165
C	-1.21215675348161	3.07968492177351	-0.21871232879919	C	-0.12593277484684	1.01225877560250	3.84216265251418
C	-1.96982924400840	1.93021086852398	-0.85093029134266	C	-0.62676854152952	-0.39138988595873	3.56130737169417
C	-2.51067887032440	2.06858978664701	-2.12778510375324	C	-1.75766199152047	-0.88047740817518	4.21277192467464
C	-3.19961143352204	1.03567520053883	-2.73856859641217	C	-2.25345458362961	-2.14571780507862	3.95323255951730
C	-3.38206264261677	-0.17242089522640	-2.07432037899985	C	-1.60978737142190	-2.97266133536756	3.03883195573470
C	-2.87739358778116	-0.34610753252525	-0.78111753287403	C	-0.44403351959830	-2.54094150890125	2.39751342305924
C	-2.16262047770339	0.70788858669411	-0.22402920123308	C	-0.00979913407158	-1.24524372317070	2.65765085615485
O	-2.34608321464878	3.27269455745470	-2.76429132075920	O	-2.36644332855850	-0.06446576060232	5.13300004109526
O	-4.08145219663572	-1.12142353028526	-2.74372965769192	O	-2.15633642144049	-4.20131874651190	2.84711485452815
C	-3.10557034295604	-1.63887157275586	-0.02113873205173	C	0.33343045886946	-3.45829862811660	1.47045584302844
C	1.58652879104794	-3.42925382661015	1.66030309127762	C	2.00968251263122	-0.85422976410658	-2.75662024771886
C	3.38788950475065	1.17443206043841	1.76324046925839	C	1.58697383135731	3.50053840300936	-0.52412025336664
C	-1.30542792611647	3.16448658777815	1.31211341653638	C	1.40335932565797	1.14647181473993	3.88359958605203
C	-3.15610962927965	-1.42707696900435	1.49672411970796	C	1.84928495595438	-3.29945873586001	1.65578568248448
C	2.94653529861292	-3.57779033954322	2.34187922860978	C	2.69420665714282	0.04716475327044	-3.78533011310525
C	3.98310681038577	2.34891431044343	2.54111877260482	C	2.21533309648082	4.78193462633844	0.02424434085172
C	-2.58860078815973	3.79233102012907	1.85802369339332	C	2.06488103112390	0.68875555661656	5.18390862330661
C	-3.87218651896520	-2.51713007223103	2.29498123751923	C	2.68854221891093	-4.51285114421704	1.25566320053135
O	4.63684504077885	0.61899286471226	-2.39153146033924	O	-2.36151283405359	3.87580019608418	-2.24338893626908
C	2.78371937405014	-3.39914499410297	3.84656457860683	C	4.05618095251662	0.49915709288953	-3.27466820160885
C	3.60151599148968	-4.91766459284712	2.02813316411962	C	2.83277474440808	-0.64658271225720	-5.13505842949537
C	-4.05099442502894	-2.04406325654598	3.73409306958095	C	4.13499796217283	-4.28668115505549	1.68238957572754
C	-3.16519340645347	-3.86738255170038	2.27291607904575	C	2.62779353593082	-4.84396383055646	-0.23093995679061
C	-2.47335882458547	3.94631954408522	3.37040680191949	C	3.54858690884883	1.03938392555010	5.14469238001969
C	-3.85926219362016	3.03073494348141	1.50206947365590	C	1.88512241063521	-0.79436536294180	5.48493883156759
C	4.04468853932369	1.98905333326801	4.02202922407407	C	3.72967120332034	4.70848142241055	-0.14108105907398
C	3.22680575669032	3.65967139485515	2.35626764763927	C	1.86683816206789	5.07245637585981	1.47940146383194
H	-0.47808766370895	-1.81788292302740	0.64227439567192	H	1.08919933698273	-1.60751413172044	-0.38369017451952
H	-3.82020160973994	-4.42214599052484	-2.45701544698005	H	-2.31257914151354	-5.60155369114278	-0.14471711084338
H	1.54776433400717	-4.92673515849401	-1.99854238642610	H	-1.65235502579148	-1.78339573607875	-4.06775081523554
H	2.10198112771582	-4.39689735705441	-0.15041760541473	H	0.32288373310519	-1.27142852398721	-3.98805759809391
H	2.21047537070111	-0.96852639779818	1.11086609026187	H	1.07446689830316	1.07364802643034	-1.24851621259980
H	2.34941636927479	-3.33506073034064	-3.45287673771396	H	-0.24152945948507	4.48280498470147	-0.91737202058901
H	4.50812147737436	1.62923031874378	0.03416819859622	H	0.71055530082067	1.68108215819052	1.36234534107542
H	0.80661718833456	1.48033304552377	0.60945859968128	H	-2.98592268208552	5.34699643336052	0.64839889011403
H	4.46055501255223	3.89801032305693	-2.28856433933664	H	-2.44621085258832	1.66396704946761	4.71634992422192
H	-0.98486190784462	4.43507492886070	-2.40787641066364	H	-0.46953512831183	1.28337137515605	4.84581629726316
H	-1.66750337837952	4.00446855204779	-0.58736939238827	H	0.88249618299022	-0.89878806403864	2.14885668866628
H	-1.76660457704293	0.57531690849789	0.77479314828274	H	-3.16490418550881	-0.48453649472928	5.47473186709179

H	-2.73997005296455	3.24140368175260	-3.64319130624016	H	-1.89475122028429	-4.57585070528993	1.98312430184688
H	-4.01271115045991	-1.99958611216780	-2.32683992127755	H	0.10806039590846	-4.48610734267627	1.77299980792244
H	-4.09753773416864	-2.00893164499107	-0.30080549292851	H	2.45297342477484	-1.85666869354265	-2.80574213511362
H	0.93685462922681	-4.24602848246041	1.99888622658899	H	2.23498743850107	-0.48054282904392	-1.75290311601818
H	1.10962758684896	-2.50753973156507	2.01076184313243	H	1.95412934255971	3.36193457823291	-1.54687229613362
H	3.92077916945750	0.26815678969394	2.07226166998699	H	1.96827804219549	2.64765525415637	0.04872395537519
H	2.34581746892133	1.03578849414719	2.07515014918924	H	1.64190863694048	2.20678314189994	3.74696297395063
H	-0.46751126004105	3.78323400959085	1.65031155734148	H	1.86945528011505	0.62551748388640	3.04016491501096
H	-1.15033628278505	2.18342262908925	1.77484937024889	H	2.03011257439341	-3.10807066025561	2.71899234804214
H	-3.68461966902768	-0.48468947082762	1.67727299250876	H	2.22027213682202	-2.41629726708606	1.12404994420347
H	-2.14737425897531	-1.29409584450429	1.90523280071525	H	2.06870751004884	0.93912077674193	-3.91805926667195
H	3.60727466535610	-2.78238551710698	1.97641468862074	H	1.84573538233649	5.61732116349273	-0.58649370547691
H	5.01209042864967	2.49420038215120	2.18437623079728	H	1.60349228040922	1.25890107341718	6.00231203927463
H	-2.66591328063173	4.79768627086172	1.42083939740421	H	2.30100066423106	-5.37686308527924	1.81315702866091
H	-4.87043923190847	-2.64690603758354	1.85465513765777	H	-2.22202341207636	4.30932271659873	-1.38095830636782
H	4.60606470174040	1.50259166348596	-1.98424535413783	H	3.95886111906037	1.05907107202121	-2.33917780700531
H	2.34879073428360	-2.42346087027643	4.08450976893760	H	4.70349567307161	-0.36427850351938	-3.08428155767842
H	2.12332428768395	-4.17005010185846	4.25904781959990	H	4.56125576718003	1.14069438651310	-4.00289839656588
H	3.74609059893574	-3.47447520654062	4.36087962139384	H	3.48942145278204	-1.51921351830297	-5.04776437424984
H	2.94596309328163	-5.74538229953844	2.32091719547765	H	1.86919458782507	-0.99063139764136	-5.51987686163610
H	3.82743126249486	-5.02785202945054	0.96442305767382	H	3.26519905807733	0.02652687392054	-5.88117871157276
H	4.54221191929565	-5.02609457263174	2.57547958058346	H	4.56340769729945	-3.43271177500159	1.14604188751808
H	-3.07693715812813	-1.88521822135257	4.20996234070795	H	4.20760866246523	-4.07947449668296	2.75425452567829
H	-4.60258383509521	-1.10036989962625	3.77895008596010	H	4.75257978673719	-5.16230833264883	1.46261783503557
H	-4.59511726819691	-2.78379596605711	4.32805727956260	H	1.61733033272457	-5.09743401592950	-0.55739494773508
H	-3.11366929219069	-4.29281115761373	1.26954208174147	H	2.96427787926827	-3.99005198945454	-0.82915945604303
H	-2.13840602216805	-3.77251527075667	2.64323670673995	H	3.28047988760241	-5.69235004525468	-0.45798126224059
H	-3.68950051409373	-4.58112536439539	2.91531502108433	H	4.05083376718855	0.49229883917634	4.33908187019439
H	-2.40308603930123	2.96470930326505	3.85200260420410	H	3.70185194190428	2.10852875036785	4.97036201122544
H	-1.58413958211308	4.52029735399397	3.64741048927573	H	4.04092856014714	0.77441969263061	6.08480996264263
H	-3.34835504822746	4.45679885354386	3.78266383276524	H	0.83459982223737	-1.06649721848953	5.60483635684652
H	-4.01345193080029	2.96681094852684	0.42309277837323	H	2.29470537341340	-1.40641190069442	4.67395311862922
H	-3.82162580024465	2.00785703229199	1.89116094805235	H	2.41115682764607	-1.06028165336840	6.40689166949375
H	-4.73154170438577	3.52504338489533	1.94014570306521	H	4.14021149342513	3.88824139465544	0.45839720597758
H	3.03678354654063	1.82551911105701	4.41933427731633	H	4.01111700033823	4.53457758676188	-1.18407334706361
H	4.62221249962892	1.07486948309423	4.18795570742024	H	4.20760250011015	5.63479870987352	0.19046736740928
H	4.50562793180193	2.79201905098716	4.60419689969751	H	0.79698886212834	5.23273705074134	1.62549946208918
H	3.25071243977036	4.01150371600402	1.32395705499733	H	2.16704459637866	4.23770894503622	2.12241797776615
H	2.17516563878435	3.54114746366901	2.63996693039069	H	2.39287268342794	5.96845139324750	1.82284298749993
H	3.65935468704285	4.44008350004934	2.98950172116394	H	-2.37748919039319	-4.59423619841979	-2.17161323459418
H	-1.76488307943582	-5.39408540168732	-2.47805781557181	H	-2.99997889205492	1.99904769476028	-3.81635415917020
H	4.10330897458235	-1.64528985306338	-3.36198064780485	H	-3.25425350262905	4.28846140195838	2.63390516221402
H	2.39893260348236	4.78099901494836	-2.66760782855310	H	-3.14623583657361	-2.50334597772949	4.45699754989843
H	-3.61073513297802	1.15672833220371	-3.73616833267592	H	-2.15428236984918	0.29638481086997	-5.62231994738757
C	1.01282904382304	-1.48702234833770	-4.61199617345696	N	-2.27949028903520	0.73851193546321	-6.64811771215190
N	1.84222159713529	-2.67634747601815	-4.85294197413790	C	-1.98525841273801	-0.30513901163212	-7.65803816253649
C	1.12184477753591	-3.61955607934119	-5.71415901565728	C	-1.37378520583147	1.90155629392309	-6.80812973364698
C	-0.19494917919852	-3.97984504384539	-5.06000404033894	C	-0.52357813323278	-0.68477488113249	-7.55907714622049
O	-0.98642721463766	-2.82844874762399	-4.81802668083737	H	-2.21690391293286	0.09479177143053	-8.64825179050493
C	-0.29583692425255	-1.91089364094473	-3.98366678884003	C	0.05901731244712	1.42144359840355	-6.74488555465582
H	0.81038997285072	-0.95803208229135	-5.55390050655867	H	-3.24771578279693	1.04122133571486	-6.73605401980267
H	1.54458939236538	-0.81045009956884	-3.93916330162292	H	-1.58152879408140	2.37155101928842	-7.77265027695827
H	2.69706779613208	-2.39076692312184	-5.31969281520689	H	-2.62453304296008	-1.16677131756404	-7.45758638974829
H	1.72689136022342	-4.52069853394909	-5.84566117745714	H	-1.58034277317524	2.61096645034991	-6.00571849866481
H	0.93265254255100	-3.18000502401514	-6.70359313641778	H	-0.31983315029457	-1.14922811951350	-6.58418923131030
H	-0.00724160015550	-4.50704850973103	-4.11543923526255	H	0.73406449082850	2.25760297810421	-6.93217015760431
H	-0.77667655031409	-4.63177040415340	-5.71469669978422	O	0.31258904323313	0.44461632589700	-7.73995002201776
H	-0.11378257562276	-2.36568432148456	-3.00047189304806	H	-0.27700999021017	-1.39957524502388	-8.34531542415367
H	-0.95119456094812	-1.04779052810413	-3.85115749936850	H	0.28035722159441	1.01145089546566	-5.74956331653304

R[4]A:*N*-methylpiperazine

CHCl$_3$				DMSO			
C	-2.68212810696802	-2.23883652070157	2.45047872264987	C	-0.18906366240389	-3.31806672876471	0.14879467131873
C	-3.11894624877130	-3.21487344252896	1.55922699863926	C	-1.18592541257472	-4.15429930429338	-0.34854681482190
C	-2.27190609689501	-4.21038545887636	1.10570197363280	C	-1.68390763659346	-4.00384065757604	-1.63153161126692
C	-0.95321740494680	-4.25170860717849	1.54264428370126	C	-1.18293586063838	-3.00806132698438	-2.46442374466738
C	-0.48213010280800	-3.30612345363639	2.46007187831730	C	-0.15723766711468	-2.16751776693749	-2.01378814803400
C	-1.35942792241121	-2.30886401708951	2.86293465533992	C	0.28538627735160	-2.33433242266568	-0.70732583846457
O	-4.42324401583216	-3.14465334283887	1.13239876471813	O	-1.66393785514182	-5.13732768296273	0.48258560187857
O	-0.15852219531265	-5.23225399027711	1.04428176902225	O	-1.71224301013567	-2.92288047288805	-3.70972332594182
C	0.92461798118818	-3.38794700334267	3.01420291583988	C	0.46035541620969	-1.12174271838157	-2.91911924787180
C	1.86369736199840	-2.41485600387079	2.33513247917215	C	-0.24038588871012	0.21258943909093	-2.76110460040780
C	2.46634755361933	-2.75380530829415	1.12245373001815	C	-1.34422323520440	0.52595101738349	-3.56182027800903
C	3.39606532896200	-1.90687914892557	0.54169275751610	C	-2.00126863366294	1.73955997196469	-3.40262574030789
C	3.66629554346160	-0.65979041088877	1.09020264434195	C	-1.56694855408475	2.66721731244088	-2.46338472971718
C	3.02352888278457	-0.25428231808462	2.26460280711981	C	-0.45621778896294	2.39124556067868	-1.65896615663924
C	2.14983294993821	-1.16075378852872	2.85786361835071	C	0.15699582368691	1.15346440833783	-1.82167811778360
O	2.11315460722800	-3.90177972492341	0.47213393323065	O	-1.78003597269465	-0.37619373363631	-4.47445764166912
C	3.24145292286199	1.13139760885734	2.84649538471990	C	0.06401754132508	3.39076012534860	-0.64194826178485
C	2.31186671678126	2.10875694114832	2.14736526325358	C	-0.61051839882365	3.16006197064714	0.69747847138020
C	2.75262745081103	2.87802061816306	1.07243924426598	C	-1.75100978748061	3.87641305542344	1.05631643831142
C	1.89548610765405	3.71204824340823	0.37773609497064	C	-2.39612200051388	3.65249073675521	2.25968255493545
C	0.56678161785301	3.82869070871291	0.76806700994778	C	-1.89779758991910	2.71248411760751	3.15550152566578
C	0.09908335338931	3.12998280145144	1.88582657184073	C	-0.73253647092767	1.99912384377286	2.85296790578575
C	0.98138662679571	2.25863134947763	2.51441339865637	C	-0.14668928082521	2.22531469578402	1.61257655238559
O	4.07695943541357	2.77863831046283	0.71832259774892	O	-2.21548135366058	4.81867620045773	0.17290364950975
O	-0.20975985386286	4.66691488745863	0.03465669517167	O	-2.58063152424657	2.56659183883264	4.32060421019837
C	-1.30088769278264	3.35197031506231	2.43076327082203	C	-0.10207533695477	1.04246757056265	3.84998312710796
C	-2.26315131683336	2.30215116553347	1.91381211469193	C	-0.60149224218949	-0.37090064896236	3.62182282865771
C	-2.98463180181570	2.51632196953779	0.74193535156318	C	-1.70845671031852	-0.85160136151192	4.31864835705031
C	-3.85849739585980	1.56672202237555	0.24358739454821	C	-2.20163391592356	-2.12636696508508	4.10437891701957
C	-4.04457700130280	0.36651904716421	0.92008244441545	C	-1.58002756024473	-2.96970802132235	3.18968130543696
C	-3.36685413024049	0.12483338585925	2.11959270576930	C	-0.43976464378042	-2.54355189562516	2.50020867083499
C	-2.47394652066752	1.09335473591578	2.56056065872357	C	-0.00710721259269	-1.23972444330928	2.71766313318803
O	-2.80090656432074	3.70945155243904	0.08858343556613	O	-2.29522417956608	-0.01592332556219	5.23537982787411
O	-4.92197808970790	-0.50770104690734	0.36714441590331	O	-2.11990991663465	-4.20798158858075	3.04481377929843
C	-3.61182667323875	-1.13926692633126	2.92010539690813	C	0.31759381187003	-3.47298400146768	1.56874764375215
C	0.92554512246771	-3.27586685425551	4.54136338729745	C	1.97826674914588	-1.02282314500889	-2.74437498640718
C	3.14280575753284	1.14928519340850	4.37660142772088	C	1.59767427430086	3.40247923132039	-0.57886539346421
C	-1.28231046477596	3.50523917278946	3.95929439891593	C	1.42715881864085	1.17877117682246	3.87582801611392
C	-3.56355759560044	-0.88263710429739	4.43227959605895	C	1.83712622618968	-3.31507834829859	1.72344182785400
C	2.27544061660437	-3.51952188397959	5.21450366511764	C	2.66703002351678	-0.25351175195523	-3.87365906527103
C	3.85428189643814	2.31256451048248	5.06816599640658	C	2.22529734420609	4.69783730523159	-0.06386312669214
C	-2.43889994459832	4.31024514434020	4.55281054978772	C	2.09849699380926	0.76292389251563	5.18501874707545
C	-4.31627580288563	-1.89655076667012	5.29444665887635	C	2.66628041814417	-4.53568827454295	1.32353681071237
O	4.56689776719828	0.10316402352860	0.41754556277468	O	-2.26413402192910	3.83475474144835	-2.40021023294390
C	2.13430289948644	-3.32833227104592	6.71991579257292	C	4.00205610665756	0.31226651248059	-3.40796067598966
C	2.83761345818777	-4.89972396486055	4.89693846035392	C	2.85892404694641	-1.13463788887415	-5.10251943968771
C	-4.37978232729923	-1.38444720364053	6.72944501040685	C	4.12283094579986	-4.30253648727245	1.70992092176484
C	-3.71783508582828	-3.29814030414702	5.26438985571263	C	2.56837350133177	-4.89400203106231	-0.15466478692100
C	-2.18515616224370	4.53420884557513	6.03972462609460	C	3.58062601959844	1.11705086945725	5.12498277556093
C	-3.80932779596756	3.67666134951604	4.34496759068525	C	1.92552874916584	-0.71137794964907	5.53012939903257
C	3.85503050956706	2.07741732371099	6.57502804864156	C	3.74273110766827	4.59970381295694	-0.17726804783324
C	3.25731682206292	3.67895433348236	4.75242222329790	C	1.83090683060176	5.05525568660243	1.36401271259706
H	-0.99424268839416	-1.55332677966390	3.55014755801414	H	1.06677510537621	-1.67455910580751	-0.34770933852507
H	-4.59105500901223	-3.82452723947722	0.46984560288907	H	-2.38335702047338	-5.61635348148014	0.05371226073080
H	0.74978665094590	-4.86229595839733	0.94096821589631	H	-1.65700334397401	-1.99729456827397	-4.05070049236897
H	1.29125956004206	-4.39093197841647	2.78295178245787	H	0.28402450080598	-1.44625103686299	-3.94933453519487
H	1.66614942823384	-0.87129363850810	3.78365591427371	H	1.01050971189175	0.92649676798046	-1.19294367067462
H	1.85846100216021	-3.63700993090047	-0.49920566553901	H	-0.22571222762320	4.38829870236665	-0.98834713155326
H	4.26945469381410	1.42841078095866	2.61303122812465	H	0.74516669707788	1.66116499443601	1.36244824260033
H	0.61588185162962	1.68838580137400	3.36188078111928	H	-3.03106217809794	5.21526947129330	0.50257242212968
H	4.24769808424950	3.30005295397267	-0.07473495992252	H	-2.42406475864869	1.68308351875667	4.70726914622750
H	-1.14749625724501	4.40386903581780	0.09779701374585	H	-0.43910170294323	1.35060943463751	4.84514045497413
H	-1.64260655780143	4.31911405183484	2.04709554632801	H	0.86606337622969	-0.89806502879419	2.17313764213789
H	-1.93891823066352	0.90473221888432	3.48374945086012	H	-3.07954828002868	-0.43248055603746	5.61256805257944

H	-3.31351608738507	3.72378678036211	-0.72755953827518
H	-4.79933831926002	-1.41133124163824	0.71311338818357
H	-4.63715048207545	-1.46058549782266	2.71070185217547
H	0.20406187814385	-4.00788377250004	4.92572521677994
H	0.55024368783378	-2.29535388301460	4.85353667649807
H	3.59299816262153	0.22034569936633	4.74310429598999
H	2.09704201833912	1.12685334328858	4.70353709338919
H	-0.35162128578750	4.01953926039931	4.22137448838512
H	-1.23213165418850	2.52957991941952	4.45526718735730
H	-4.00965713531435	0.10184890720661	4.61042159129877
H	-2.52858994818774	-0.81441385600223	4.78643680133843
H	2.98571301595916	-2.77058632855863	4.84358608123957
H	4.89804861728249	2.30935179235835	4.72477321087522
H	-2.44045697274608	5.29102205444955	4.05713783652230
H	-5.34381111623957	-1.95594328839082	4.90960718595131
H	4.46720701867513	1.05138768576132	0.62398197214110
H	1.75321194612100	-2.33046718687033	6.95818687075543
H	1.43733689032847	-4.06183787114926	7.14025489319028
H	3.09547799487642	-3.45253115172328	7.22689312535609
H	2.12452074838765	-5.68240798966658	5.17910938968699
H	3.06310287777630	-5.01764836029132	3.83383895904105
H	3.76477004956206	-5.07659815887901	5.44959121121377
H	-3.37230138559513	-1.30005351231099	7.15165031237448
H	-4.84783580902641	-0.39702877545761	6.78102290195794
H	-4.95177480397375	-2.06487124982080	7.36657162685978
H	-3.74516904581621	-3.74001664477207	4.26684742070037
H	-2.67114838926025	-3.27814170596559	5.58724279306641
H	-4.26567125639423	-3.95981888271866	5.94193384234446
H	-2.17536487315778	3.57827351754178	6.57490136384635
H	-1.22279177902964	5.02501405331908	6.21213788425782
H	-2.96760770497182	5.15596283769322	6.48393435764192
H	-4.06570304198907	3.58176357207148	3.28805880052611
H	-3.84272966156734	2.67384521381993	4.78422041776344
H	-4.58327911924511	4.28102601371127	4.82761515059887
H	2.83038304839303	2.06501719599629	6.96292365508033
H	4.32047405079310	1.12086753961460	6.83045478280967
H	4.39892750360384	2.86948023134804	7.09753315636736
H	3.32366634640965	3.92562047678065	3.69122518862642
H	2.19856646470509	3.71220765412992	5.03244576836465
H	3.77809076051508	4.46031349706307	5.31401984120414
H	-2.61641040828488	-4.95064452367040	0.39010971782773
H	3.88341558992765	-2.19298364810780	-0.38370987617927
H	2.24638167794589	4.28068374764125	-0.47795166941702
H	-4.40224603715513	1.74339424197682	-0.67937187382481
N	1.24165444812184	-3.27022801934175	-1.93751127704579
C	1.74781997771648	-2.10658736322062	-2.67613470360064
C	-0.21519499697041	-3.16584440022876	-1.79056531389438
C	1.35652575190071	-0.81871180673887	-1.98087437289896
H	1.34563548501211	-2.10393799636888	-3.69888482326681
C	-0.55980215811311	-1.89020552784022	-1.05421167509589
H	-0.57446870510877	-4.02977051781516	-1.22883483459060
H	1.45441064094053	-4.10630924281798	-2.47342905372659
H	-0.70411777200145	-3.16063943338534	-2.77481724981261
H	2.83708671496568	-2.17207227917043	-2.73760818248524
N	-0.08325810304711	-0.72772076480859	-1.78828753762544
H	1.68332484986784	0.02703839429929	-2.59384003144912
H	1.88127415109811	-0.75019849496586	-1.01433147417036
H	-0.13015868909584	-1.92472688213042	-0.03739512124031
H	-1.64566608142505	-1.81727818658303	-0.94319390147468
C	-0.40813866346446	0.48821472165797	-1.06857423732799
H	0.07530355762802	0.52667811393112	-0.07617405342547
H	-1.48850049236987	0.55809594976767	-0.92231614907203
H	-0.07647145167211	1.35922201855253	-1.64029005975517
H	-1.89506575414203	-4.58920766122262	2.17390293697278
H	0.09642783702632	-4.49703191947537	1.88639027571957
H	2.39346018867097	-2.03671610186301	-2.69615854595588
H	2.22270754732505	-0.55589670293455	-1.78558209172096
H	1.96917997265892	3.23512655582666	-1.59556542211014
H	1.97417958895608	2.56449363813980	0.01818591922417
H	1.66455357414273	2.23404794224797	3.70290062599321
H	1.88659808378378	0.62901911509230	3.04709390876171
H	2.03738657521261	-3.11107139287276	2.78082920758024
H	2.20111073058217	-2.43903150472000	1.17533240174356
H	2.02115381798549	0.59028301901391	-4.15195742100624
H	1.88626005575155	5.51006396114898	-0.72172259376851
H	1.64060926761355	1.35554238877826	5.98926713616141
H	2.29227366925127	-5.38939260730083	1.90555408788325
H	-2.18389387818110	4.24551645210702	-1.51795206709878
H	3.86745260981938	0.99988170299643	-2.56707357690765
H	4.66983047873670	-0.49204598137198	-3.07945991865951
H	4.50547455470051	0.85518329688927	-4.21347432381117
H	3.55045472528598	-1.95391690484113	-4.87723072347689
H	1.91685612736111	-1.57546126507082	-5.44017536489591
H	3.27378205234588	-0.56318164582885	-5.93770687512486
H	4.53759034741834	-3.45891614253754	1.14711963362985
H	4.22209891513060	-4.07546299672579	2.77558375507240
H	4.73469929780002	-5.18237581813312	1.49112514216650
H	1.55042285942000	-5.15522485263379	-0.45043991540096
H	2.88823549657610	-4.05145171881672	-0.77756740854486
H	3.21621145794369	-5.74594560294943	-0.38217097826617
H	4.07906074156979	0.54802339605367	4.33231828011831
H	3.72877734076658	2.18104425441297	4.91783301010729
H	4.08078678481037	0.88196952618019	6.06892304406885
H	0.87686737663249	-0.98233914181169	5.66746104950154
H	2.32936829965160	-1.34562814684717	4.73333049942667
H	2.46036334057772	-0.94994826604938	6.45449815995733
H	4.12405765767578	3.80263653427365	0.47072401406015
H	4.05428520974839	4.37601182501139	-1.20207485139245
H	4.22164283763430	5.53462761576397	0.12749941993430
H	0.75847793406298	5.23376305654929	1.46398061639637
H	2.09750397023831	4.24719507558452	2.05400257349846
H	2.35506313558555	5.96037108135862	1.68556332268241
H	-2.47272299697823	-4.65458386489516	-1.99662269765710
H	-2.87483506998317	1.96747332671124	-4.00350179374387
H	-3.29400216843910	4.20598100473677	2.51727507766020
H	-3.07571367981011	-2.47884955275654	4.64329756847561
H	-2.04283331915774	0.07221171846502	-5.39819600601825
N	-2.32693971624437	0.56411217573265	-6.80490710774818
C	-1.51092756769385	-0.25564076743401	-7.70856083427022
C	-2.06713321240312	1.98906029004017	-7.03766086366363
C	-0.04675445549237	0.06055581518305	-7.48991001151767
H	-1.78038120754590	-0.06204550407025	-8.75509396442320
C	-0.59662608518423	2.28174097775350	-6.83375978292714
H	-3.30948564994601	0.37326807017023	-6.97577704976751
H	-2.35664172838805	2.26783447691579	-8.05963181896019
H	-1.70051169141220	-1.30987864023435	-7.49259507686868
H	-2.66673759628408	2.57651850980733	-6.33933006417525
H	0.23786292846176	-0.22832546626092	-6.46187585824221
H	-0.40704361590047	3.33852122730617	-7.04212715496927
N	0.21678664159698	1.47139430137649	-7.72693877449460
H	0.55953961953489	-0.53390859728249	-8.17909032013805
H	-0.32615808252658	2.09719405647792	-5.77720659270895
C	1.62324446346404	1.77151756094140	-7.55056239522980
H	1.96694655733993	1.57659215032935	-6.51991134038768
H	1.80732851305891	2.82445876694932	-7.77907889256488
H	2.22003071049791	1.15893063765740	-8.23103461094546

R[4]A:dipropylamine

DMSO			
C	-0.54894533414278	-3.83697226838031	0.45577138441533
C	-0.75463221619290	-4.60350145729316	-0.68720678772490
C	0.27637887266291	-5.31635401444718	-1.27688634803883
C	1.55614673569532	-5.28969132080194	-0.72579108294016
C	1.79352235479081	-4.55691259242647	0.44812272677284
C	0.73583736133708	-3.83354955577959	0.98342821631841
O	-2.02163019888835	-4.62295846203446	-1.22000696300858
O	2.51911733975550	-6.00112987022362	-1.34745667972981
C	3.16047276286229	-4.56506387542456	1.10658144793110
C	4.03302285954120	-3.45014983548159	0.56817753943854
C	4.79695395007653	-3.67477645726177	-0.59719823000116
C	5.57612036465789	-2.62293114256076	-1.09400205914966
C	5.60945697994659	-1.38299213769916	-0.46726167557398
C	4.87928198566691	-1.15388302042371	0.70201637084461
C	4.09176614381346	-2.20338776161852	1.16928917545512
O	4.77102536744348	-4.85037597217965	-1.21320594098026
C	4.92328178015810	0.17887875967805	1.42769005581398
C	3.82645664053607	1.08609497712735	0.90239601403651
C	4.08355572477249	2.01585046092249	-0.10119648454923
C	3.07991496898563	2.81105232281552	-0.62893975640012
C	1.77425023391266	2.70506029753844	-0.15287276741161
C	1.48501808122944	1.80741107491848	0.88566081120315
C	2.51696423510639	1.00765810390929	1.35977505932514
O	5.37650190811709	2.11614268607897	-0.55944361975945
O	0.83676811594468	3.50129836404659	-0.70592653460793
C	0.09205940413750	1.72919101993184	1.48429200773710
C	-0.74403178674989	0.69615499558413	0.75555548260902
C	-1.45863179405044	1.08747312331824	-0.39842734711060
C	-2.23283397780175	0.12823196112671	-1.06062289906466
C	-2.29683453890661	-1.18709155118217	-0.61622783775956
C	-1.59900188855834	-1.58890949084961	0.52607958634150
C	-0.82041677850315	-0.62443180125099	1.16339874505251
O	-1.39599912611168	2.33354015548978	-0.84954118869850
O	-3.08434294489430	-2.03718788537061	-1.34245932792968
C	-1.67114301126848	-3.01099361995754	1.05452976538472
C	3.06520624911298	-4.57889341031669	2.63345584352509
C	4.92405883273517	0.00668025040421	2.95347642857660
C	0.14170254761361	1.58389223755861	3.01103967374735
C	-1.73787631811826	-3.05288571305676	2.58742115530145
C	4.39418988130090	-4.80538285442969	3.35272424819678
C	5.53638358506351	1.16406008648841	3.74281492060938
C	-1.09701134418740	2.08006358470297	3.76014078624009
C	-2.36099713283221	-4.31441357016393	3.18614970666972
O	6.40127173240170	-0.43069513747337	-1.04429424857389
C	4.23052401000203	-4.54742456366536	4.84575533557621
C	4.94157303883677	-6.20653550302310	3.10981067827696
C	-2.51365262053866	-4.13803998816534	4.69329245202792
C	-1.58761172444015	-5.59262445136390	2.88482045233825
C	-0.78571673071265	2.18026513277263	5.24996393812063
C	-2.33520710607985	1.21901798034477	3.54372354413280
C	5.65607178467845	0.76834298794473	5.21086248395617
C	4.76902562157024	2.47430415879035	3.61355171902503
H	0.91685300618689	-3.24588734367694	1.87737288050428
H	-2.03127091804016	-5.14037650470962	-2.03426144756408
H	3.43342666539711	-5.56324885931804	-1.22334265455067
H	3.63967454014612	-5.50314401382690	0.80624421420751
H	3.50801858852827	-2.03643276688580	2.07006939610136
H	5.88377799098591	0.65003238712280	1.18790797739417
H	2.29045053747013	0.29916665349786	2.15092924504614
H	5.41893771915246	2.73716143499224	-1.29674668172445
H	-0.07774097824132	3.04225453652706	-0.69138203391848
H	-0.38368311503562	2.69902521539487	1.29018196522265
H	-0.27064225683497	-0.92474421241782	2.05067359098526
H	-2.78245650345595	-2.95862866119513	-1.23961844815514
H	-2.61800417858326	-3.44194530894122	0.70872474665517

R[4]A:diisopropylamine

DMSO			
C	0.24680831404755	-3.28813363794180	-0.19395472925174
C	-0.85971055596321	-4.06973279831546	0.12356839544253
C	-1.44201038793705	-4.02534331290871	1.37988922834128
C	-0.92268973818218	-3.19077344395668	2.36841978700290
C	0.21306415378043	-2.41185982715558	2.09396935510054
C	0.74320019492683	-2.46911336116035	0.81211207929671
O	-1.36397465844066	-4.88709427589395	-0.86117855692010
O	-1.53417837761500	-3.18372623304008	3.56968529217181
C	0.83204555759930	-1.53795058168802	3.16924434297727
C	0.22071054049069	-0.15223015058456	3.16067432744450
C	-0.96814957824520	0.08103800676264	3.88760127762487
C	-1.53003746013274	1.36339651952003	3.84399771417659
C	-0.94361384332213	2.39236296169507	3.11663583444469
C	0.24485153364993	2.18209962618212	2.41190024558803
C	0.77786102307150	0.89548763357717	2.44669128952419
O	-1.53880774083693	-0.88550429364258	4.59078026344940
C	0.92165004628295	3.29045220525175	1.62362962828567
C	0.39558888056427	3.29886658902716	0.20049945063012
C	-0.65065505619102	4.13681728810707	-0.17404465349194
C	-1.17428868391018	4.11398733992790	-1.45606439840830
C	-0.65041260933629	3.25047940366966	-2.41684389155676
C	0.43228398149420	2.42069028910074	-2.08739162275537
C	0.89917632174190	2.45288298968226	-0.77994106156573
O	-1.15574453053746	4.98994001406697	0.77993829822556
O	-1.20461277742542	3.27107112248994	-3.64601247953120
C	1.07928057227783	1.52750559773057	-3.13201734256508
C	0.40734824695667	0.16986194245340	-3.15351350557429
C	-0.73515792909419	-0.01011463827440	-3.96588128560722
C	-1.35873499510598	-1.26400448786964	-3.96330688211989
C	-0.87602885867704	-2.31563321993219	-3.19299421151957
C	0.26692604786617	-2.15932647649946	-2.40581678468624
C	0.85793734180807	-0.89701748942790	-2.39659128501433
O	-1.20312456542621	0.98241129905800	-4.70704330884632
O	-1.56677300630153	-3.49309887848642	-3.27153031384819
C	0.84247632973763	-3.30241371997103	-1.58809073014004
C	2.36044154313218	-1.53138500018391	3.10165978658560
C	2.45117573842004	3.22084037846120	1.72994941562861
C	2.60683187306017	1.50142798973380	-2.99432935166826
C	2.37715737672784	-3.31352711584692	-1.61829531721519
C	3.04779194908675	-0.84791885775637	4.28327559811865
C	3.19783619075963	4.52425808333178	1.44384551388107
C	3.37258440611241	1.18215891363984	-4.27979412924295
C	3.03822478898899	-4.65894693213729	-1.31731862649218
O	-1.57854189885780	3.60205910996590	3.15375888755193
C	4.53634730375775	-0.68780908011264	3.99719255706440
C	2.83183004967314	-1.60603426828711	5.58771019041458
C	4.53913462057502	-4.54900563532463	-1.56621087933352
C	2.77936116214942	-5.17750945002017	0.09256821216348
C	4.86499661426105	1.39711509748442	-4.05146362425693
C	3.12312694953593	-0.22029740648676	-4.82136732043403
C	4.67575020246553	4.33964771069855	1.77285799316651
C	3.04178562008661	5.03146581931887	0.01466169141754
H	1.60637195011905	-1.85153915265771	0.58623420038668
H	-2.16845163534829	-5.32200449726434	-0.55360936116235
H	-1.46661556027399	-2.25568112441291	4.00666353974385
H	0.55529935858285	-1.99065219993063	4.12764411525323
H	1.69662724135856	0.71429728481327	1.89636339441866
H	0.63841761680288	4.24337761865202	2.08616700405125
H	1.72047842443997	1.79353909295997	-0.51576378879968
H	-1.91896843114085	5.46599382988777	0.43065472387592
H	-1.13898750611528	2.34978760400908	-4.09046975637582
H	0.87141191724331	1.98977314505227	-4.10537977822743
H	1.74231268238516	-0.75917680767765	-1.78189119707380
H	-1.43167030956165	-4.02782965577221	-2.46723564204538
H	0.53348679902868	-4.23763921644855	-2.06941150177893

H	2.36340864910844	-5.37080643394623	2.92858787331157
H	2.62726933478900	-3.64296147129165	2.99762438014415
H	5.50579674321528	-0.89479886257174	3.17994195805155
H	3.91245610088928	-0.18699868607662	3.33061211033368
H	0.99683012980183	2.17356506924707	3.36326070022193
H	0.35104225231876	0.54994481562523	3.31233694136063
H	-2.34337964191010	-2.19779261230409	2.91040548201361
H	-0.74620989250226	-2.89882266671905	3.03029774482899
H	5.12026818029145	-4.07996470125239	2.96098799352403
H	6.55172928569283	1.32590110818425	3.35128055757256
H	-1.31853281876023	3.09290199042962	3.39139012211552
H	-3.36738676640717	-4.41878053439045	2.75394653820387
H	6.09870523232214	0.46517006904219	-0.80482096700828
H	3.88568223426689	-3.52553782858657	5.03518558846738
H	3.49464634994474	-5.23455799167634	5.28005148804684
H	5.17539934407286	-4.69007970829075	5.37965966944823
H	4.23926679736957	-6.96168874367935	3.48214533582843
H	5.11418920394546	-6.40425591612267	2.04848373784409
H	5.89464071870052	-6.35024295172911	3.62856811583124
H	-1.53123161051112	-4.04201008466066	5.17034333237771
H	-3.09213231558211	-3.24039845528094	4.93442217976776
H	-3.01892179926329	-4.99841773895545	5.14298567900532
H	-1.54438869728077	-5.80610064835759	1.81454469772159
H	-0.55587422622727	-5.51620291003357	3.24694040324727
H	-2.05716593830989	-6.44747383886114	3.38245898858269
H	-0.54160123873237	1.19277960172423	5.65919597894588
H	0.06798799368585	2.83959334256729	5.43651994335019
H	-1.64320095844769	2.56865354208559	5.80848633837419
H	-2.61732865673903	1.16449082906124	2.49100055140184
H	-2.15514341474843	0.19382629872203	3.88797247859203
H	-3.18442030125874	1.61983639325430	4.10704348940054
H	4.66444426378594	0.58818871525535	5.64234855352383
H	6.24410270559043	-0.14716343977302	5.33119200180530
H	6.13555391674169	1.55913928626261	5.79616541851231
H	4.73899636704629	2.83505775810961	2.58306888641672
H	3.73284755677964	2.35154985130958	3.94989229171838
H	5.23312251278834	3.25034736295517	4.23103955347359
H	0.09956657715835	-5.89419772411695	-2.18047474694136
H	6.14806514574243	-2.76395607505031	-2.00671468102053
H	3.29971425176590	3.51755635520582	-1.42528406543771
H	-2.77821734898857	0.41108824245985	-1.95704674540196
N	-3.51379723771508	3.59986717075679	-1.53885147824531
C	-4.73863666495156	3.06329420040280	-0.89909332402465
C	-3.54769326766767	3.63773002574058	-3.01858602816186
C	-4.53228738249058	2.79643885494364	0.57993483156048
H	-4.98831975049207	2.13896562355565	-1.42648925857152
C	-4.57083161029307	4.60435348580221	-3.57950590000755
H	-3.34026788199450	4.54437364552866	-1.19225951892006
H	-2.54095038453523	3.91744852966588	-3.34298876530386
H	-5.55148005367893	3.77738323319190	-1.05447245929576
H	-3.73708916450134	2.61490086629436	-3.35788635867106
H	-5.47587333259602	2.39129490578734	0.96048835541967
C	-4.47795065107833	4.67675065187813	-5.09616100855693
C	-4.13562282417867	4.02393315673511	1.38685236830901
H	-4.40007304689924	5.59707506835129	-3.14480819625206
H	-3.78477313105204	2.00570400598796	0.70288697562615
H	-5.58031303830944	4.29743912603875	-3.28737479792136
H	-4.66400279668323	3.69797881792647	-5.54920055722928
H	-3.48614636236290	5.01144106189675	-5.41571024896516
H	-5.21447933261350	5.37671124845226	-5.49807664902845
H	-4.08727355503681	3.78220018502484	2.45158573899698
H	-4.85896815831128	4.83559324129088	1.25763765569992
H	-3.14821806297840	4.40001667175555	1.10000553192411
N	7.01350512665358	-5.82181937675496	-1.99032030138438
C	8.10767102137763	-5.33501745185295	-1.11777459812594
C	7.17179373020594	-5.50884728534330	-3.42943250271878
C	7.82248486929455	-5.61749148987507	0.34550831762501
H	8.19744825786209	-4.26095959366419	-1.29904647388770
C	8.32243221353877	-6.24026066741692	-4.09100382427564
H	6.92234227624984	-6.83341500699549	-1.88440049409400
H	6.22518203682692	-5.77670625843524	-3.90806795871232
H	2.70803801248697	-2.57175155934787	3.04314567579151
H	2.70090730580758	-1.05024211461843	2.17800521405409
H	2.69122322929986	2.91948379239834	2.75673211425421
H	2.85669654111362	2.43036193537056	1.08667352320826
H	2.92402555419885	2.49781691271174	-2.66308291109609
H	2.92693099461014	0.81202804645944	-2.20350749812838
H	2.68388552297861	-3.01171012804362	-2.62692221494737
H	2.79129887658461	-2.55460736667338	-0.94340685285380
H	2.61382074467852	0.15532089163456	4.39354799561198
H	2.79229192791805	5.29033862729025	2.12136962897636
H	3.03974188315832	1.90236098296761	-5.04218514981480
H	2.62913621596713	-5.39202083222253	-2.02840374677779
H	-1.36233916853527	4.13340508325846	2.36466696183148
H	4.70363684231487	-0.09645134819761	3.09088107589429
H	5.01041861368749	-1.66558153737600	3.85075356000511
H	5.04929699156349	-0.18899657233455	4.82553600303229
H	3.24081463577745	-2.62069457136154	5.51465244082334
H	1.77258902938623	-1.68896655732761	5.84580418571961
H	3.33378302503255	-1.10165237104600	6.41948719383709
H	4.99009496176866	-3.82361468308934	-0.87874106765603
H	4.75179325806155	-4.21784933867516	-2.58773962339067
H	5.03849355529574	-5.51038305684954	-1.41030004877108
H	1.71932927865717	-5.36831347144832	0.27393867323843
H	3.11515405501972	-4.45021457191508	0.84069432439948
H	3.32570500824290	-6.11129583632909	0.26153109873919
H	5.24144548813732	0.70445020420125	-3.28953936854639
H	5.07430680655367	2.41571014282877	-3.70920517937244
H	5.43525409596975	1.22384928227829	-4.96951390548420
H	2.07274727388197	-0.38312185310747	-5.07176152412254
H	3.40358998616310	-0.97722456653921	-4.07990104866636
H	3.72084798760082	-0.39301001228674	-5.72253982352808
H	5.12266225723628	3.58091390306553	1.11968847605189
H	4.81770932469450	4.01453519212481	2.80855859528051
H	5.23216105510741	5.27125039126218	1.63002303546288
H	2.00505289386934	5.27571858367658	-0.22686592889833
H	3.37756291031178	4.27500602683434	-0.70406901550601
H	3.64647584024884	5.93165595182521	-0.13693080529778
H	-2.31684774088892	-4.63095848361944	1.60295410627343
H	-2.45755023152686	1.55756690867212	4.37534388268371
H	-2.00454435157063	4.76278905527847	-1.72346081249801
H	-2.25435336659401	-1.41938788123912	-4.55784639883514
N	-2.36528203661099	0.58526405028060	-6.98734920925885
C	-1.73245687072874	-0.61115931054889	-7.62408175765814
C	-3.86370452120151	0.56839355248363	-6.88742148582399
C	-0.22556813059729	-0.43918319629900	-7.57111424127878
H	-2.02726764778404	-1.45561872543226	-6.99225082815165
C	-4.47756588426602	1.39476698102094	-8.00221690043343
H	-2.07898309288762	1.41922085642943	-7.50535915589369
C	-4.29907912549375	1.07381927791523	-5.52409401814264
C	-2.22226601612728	-0.84076602563330	-9.03963096238394
H	-4.15729559120715	-0.48063342888255	-6.99204872536063
H	-4.19434593091807	2.44711376134034	-7.89269943311420
H	0.12337996640104	-0.28920948474618	-6.54765642074092
H	-4.16755283518861	1.05382970261321	-8.99155169138207
N	-2.29725632050607	-0.38647698920692	7.03251053607481
C	-1.53125531872860	0.82227732660224	7.47001762969811
C	-3.79268877031719	-0.30655666438815	7.16222377420074
C	-0.05264287238448	0.55573853797762	7.25680728698484
H	-1.86268170323342	1.62271422087828	6.80073324882086
C	-4.25148108470349	-1.05326638310291	8.40108089795645
H	-1.96521445427542	-1.19006083786125	7.57074434235615
C	-4.45491649280993	-0.85093271312632	5.91005216510134
C	-1.82839410760231	1.20093798998183	8.90696137008237
H	-4.02942208626812	0.75750399413949	7.25522175992023
H	0.51866669655388	1.44573469086779	7.52977655185475
H	-4.01828771766725	-2.11932754344316	8.30797103604908
H	0.16812345205488	0.31090375781882	6.21618043015840
H	-3.78214486866937	-0.67423656906924	9.31095213406345
H	-2.01729437589328	-0.59444868031465	5.99648670581910
H	-1.91396479360224	0.70098067290854	-5.99206021144543
H	-5.56669316510410	1.33144653468189	-7.94543901435494

H	9.03995817246947	-5.81596150126296	-1.42486053458846	H	-3.89003012144694	0.46303511998125	-4.71741402164333
H	7.29193839551315	-4.42455213223395	-3.50973637428720	H	-3.97674552811205	2.10823461600714	-5.37116115850952
H	8.63256005639878	-5.15608294699231	0.91977297617823	H	-5.38981022087935	1.04338406801691	-5.46590534673139
C	8.35587895678120	-5.96020011472240	-5.58583444935390	H	0.28492344339582	-0.27078444389614	7.89120758012246
C	7.74134975552005	-7.10134307366924	0.67459226834809	H	-5.33400249830725	-0.95323284186705	8.50818182929171
H	8.21198756061264	-7.31696250900141	-3.91246793258082	H	-4.14579128765386	-0.29979141213905	5.02031068345741
H	6.89944844793076	-5.10819205937636	0.64533576909335	H	-4.20734826858607	-1.90696141272107	5.76479414245538
H	9.27285361336835	-5.93821482758001	-3.63958237269694	H	-5.53900076333300	-0.76583439825310	6.01645334623264
H	8.48267536527860	-4.89123748648884	-5.78344694356100	H	-2.87844163632915	1.45474408358108	9.06536727987475
H	7.42847515038531	-6.28281797337689	-6.06917382807483	H	-1.55359318059703	0.39077701930273	9.59001533606950
H	9.18487075685194	-6.48913738415657	-6.06217688770017	H	-1.23288982644969	2.07920742646991	9.16757317064571
H	7.64419904611838	-7.25200383680930	1.75224015091398	H	0.25588173002024	-1.33290482610711	-7.97400262412531
H	8.64056538208559	-7.62805794670112	0.33898583790498	H	0.08759841632478	0.41742411427416	-8.17745580604026
H	6.87428989212698	-7.58032722690980	0.20788850576590	H	-3.29455317662860	-1.04187154565853	-9.08367264045726
H	6.05343091700318	-5.38559534905938	-1.64993422669895	H	-1.99803821331632	0.02223232029904	-9.67474151612199
H	-2.63149551134250	3.00467112014663	-1.23568965561710	H	-1.70426106367473	-1.70930572290860	-9.45324722515573
